# Results From the WAGR Syndrome Patient Registry: Characterization of WAGR Spectrum and Recommendations for Care Management

**DOI:** 10.3389/fped.2021.733018

**Published:** 2021-12-14

**Authors:** Kelly A. Duffy, Kelly L. Trout, Jennifer M. Gunckle, Shari McCullen Krantz, John Morris, Jennifer M. Kalish

**Affiliations:** ^1^Division of Human Genetics and Center for Childhood Cancer Research, Children's Hospital of Philadelphia, Philadelphia, PA, United States; ^2^International WAGR Syndrome Association, Montgomery Village, MD, United States; ^3^Department of Genetics and Pediatrics, Perelman School of Medicine, University of Pennsylvania, Philadelphia, PA, United States

**Keywords:** WAGR syndrome (Wilms tumor, Aniridia, Genitourinary anomalies, and range of developmental delays), 11p13 deletion, rare disease registry

## Abstract

WAGR syndrome is a rare genetic disorder characterized by Wilms tumor, Aniridia, Genitourinary anomalies, and Range of developmental delays. In addition to the classic features, patients affected by WAGR syndrome can develop obesity and kidney failure, and a wide variety of non-classical manifestations have also been described. This suggests that a broader phenotypic spectrum beyond the classic syndrome exists and here we demonstrate that spectrum using data from the WAGR Syndrome Patient Registry. In the present study, we collected information from 91 individuals enrolled in the registry to explore self-reported health issues in this patient population. A wide variety of common clinical issues not classically associated with the disorder were found, prompting the redefinition from WAGR syndrome to WAGR spectrum disorder to incorporate the phenotypic variations that occur. A comprehensive care management approach is needed to address the wide range of clinical issues and we propose a care model for patients affected by WAGR spectrum disorder. Further research is needed to solidify the breath of the phenotype and confirm the observations in this study to advance individualized patient care in this population.

## Introduction

WAGR syndrome is a rare genetic disorder defined by the acronym of the features classically associated with the syndrome: Wilms tumor (WT), Aniridia, Genitourinary (GU) anomalies, and Range of developmental delays. The disorder is caused by a deletion in chromosome 11p13 (1). Deleted genes in the region including *WT1* and *PAX6* are thought to cause the phenotypic features and clinical issues in patients; however, much is still unknown about the specific molecular role of these genes in patients with WAGR syndrome. More recently, deletions in *BDNF* have been found in ~50% of patients with WAGR and the *BDNF* gene has been associated with obesity (2). Other candidate genes in the 11p13 region have also recently been implicated in regard to WT risk and behavioral/cognitive functioning.

Information and data regarding the full phenotypic spectrum of patients affected by WAGR syndrome are not currently complete. Aniridia, characterized by complete or partial absence of the iris, is the most common feature in patients and is often the presenting sign of WAGR syndrome. Approximately 50% of patients develop WT and tumor screening is recommended at time of first suspected diagnosis to allow for early detection. Current tumor screening guidelines include renal (kidney) ultrasounds every 3 months until the age of 7–8 years (1, 3). In addition to the classic features of the disorder, patients have also presented with obesity, kidney failure, and additional ocular issues (1, 2, 4). The majority of previous cohort studies have focused on a specific feature of WAGR syndrome, most commonly WT, cognitive/behavioral issues, or obesity. A variety of case reports and series have been reported, which provide information on additional issues that are not typically associated with WAGR syndrome or described in large cohorts.

A previous study from 2005 reported on the health status of 54 patients with WAGR syndrome (1). The results of this study broadened the WAGR phenotype and showed a variety of “non-classical manifestations” in addition to the classic features of the syndrome (1). Guidelines for health supervision in children with WAGR syndrome were also proposed, with some changes in clinical management recommendations based on the study results. The main changes suggested included consideration for screening patients for early signs of kidney failure as well as the potential for lifelong WT screening within the WAGR patient population (1). Since 2005, limited evidence has been published on the overall health status of patients with WAGR syndrome or on additional guidance for care management. Care management is a major concern among families in the WAGR patient community, highlighting the need to perform additional studies.

In the present study, we utilized data collected through the WAGR Syndrome Patient Registry to evaluate the rates of self-reported health issues in a large patient population, the WAGR Discovery Cohort, which consisted of 91 individuals. The main objective was to determine the frequency of specific health issues to identify those potentially associated with the disorder. Secondary objectives included identification of commonly affected health categories to determine care management recommendations, and investigation into the phenotypic characteristics.

## Materials and Methods

### Participants and Data Collection

Participants and data were collected through the WAGR Syndrome Patient Registry, an online specific rare disease registry platform managed through the Coordination of Rare Diseases at Sanford (CoRDS) in partnership with the International WAGR Syndrome Association (IWSA). Researcher access to the data utilized in this study was obtained through CoRDS. This research was not considered human subjects research as the provided dataset was de-identified.

#### CoRDS Registry

The CoRDS Rare Disease Registry is an online database in which patients affected by rare diseases can enroll and provide health information. At time of enrollment in the CoRDS registry, the individual or parent/legal guardian provide consent and basic information about demographics and rare disease information.

#### IWSA Questionnaire

The IWSA questionnaire is a disease-specific questionnaire created for patients in the WAGR Syndrome Patient Registry through CoRDS. Participants can provide information on a variety of health issues, with questions organized into health categories (i.e., Endocrine, Cardiology, Kidney Issues, etc.), with additional questions about specific health issues within each category. In addition to health information, the questionnaire gathers information on genetics, birth, and psychosocial data. Two primary response methods are utilized in the questionnaire, depending on the question type: checkbox selections or multiple-choice options. For health status questions, the multiple-choice options were: “Currently a problem;” “Not a problem today, but was in the past;” “Never a problem;” or “Unsure.” All questions are optional, and participants can choose not to respond to particular questions or sections. Participants have the opportunity to update their information at any time and are encouraged to update their information annually with longitudinal data collection.

### Data Analysis

CoRDS data were provided in the form of an Excel database with two spreadsheets: CoRDS enrollment information (de-identified) and the IWSA Questionnaire. For all questions or sections that were not completed by the participant, the data input was “NULL” for relevant columns in the provided dataset.

#### WAGR Discovery Cohort

The dataset provided by CoRDS was reviewed to determine eligibility for the study. Eligible participants included those with a self-reported diagnosis of WAGR syndrome in the CoRDS enrollment and/or IWSA Questionnaire. Participants in the initial dataset who reported a diagnosis other than WAGR syndrome (such as isolated aniridia or other chromosome 11p abnormalities) were excluded.

#### Discovery Cohort Database

Participants who updated their information at least once had multiple rows in the database, with one row respective to each entry. For these participants, the data from each individual entry were reviewed and summarized to create a single composite row for each participant. Participants who completed the questionnaire a single time had a single row of data provided. Individual spreadsheets were created to evaluate the questions.

For each specific health issue question, participant responses were grouped as: Affected (response of “Currently a problem” or “Not a problem today, but was in the past” or checkbox selection) or Not Affected (“Never a problem” response). Participants were also grouped to evaluate health categories as: Affected (one or more issues in the category reported or selected); or Not Affected (all issues in category reported as “Never a problem” or the “None” checkbox was selected).

#### Descriptive Statistics

The Excel database was used to perform descriptive statistics and data were summarized as frequencies. The evaluated frequencies were considered the proportion of affected participants compared to the total number of participants who provided a valid response for the question evaluated (affected and not affected). Participants who did not complete a specific question or category in the questionnaire (“NULL” response) and those who responded as “Unsure” were not included in the total count of valid participants for each question and excluded as applicable to the relevant questions.

## Results

### WAGR Discovery Cohort

A total of 91 participants who selected a diagnosis of WAGR syndrome were identified through the CoRDS registry, with enrollment completed between August 2014 and May 2020. Participants were enrolled and surveys were completed by a parent of a child under the age of 18 years (*n* = 66) or the legally authorized representative if the participant was an adult (*n* = 24); one participant was an adult who self-enrolled.

#### Cohort Description

**Figure 1:**
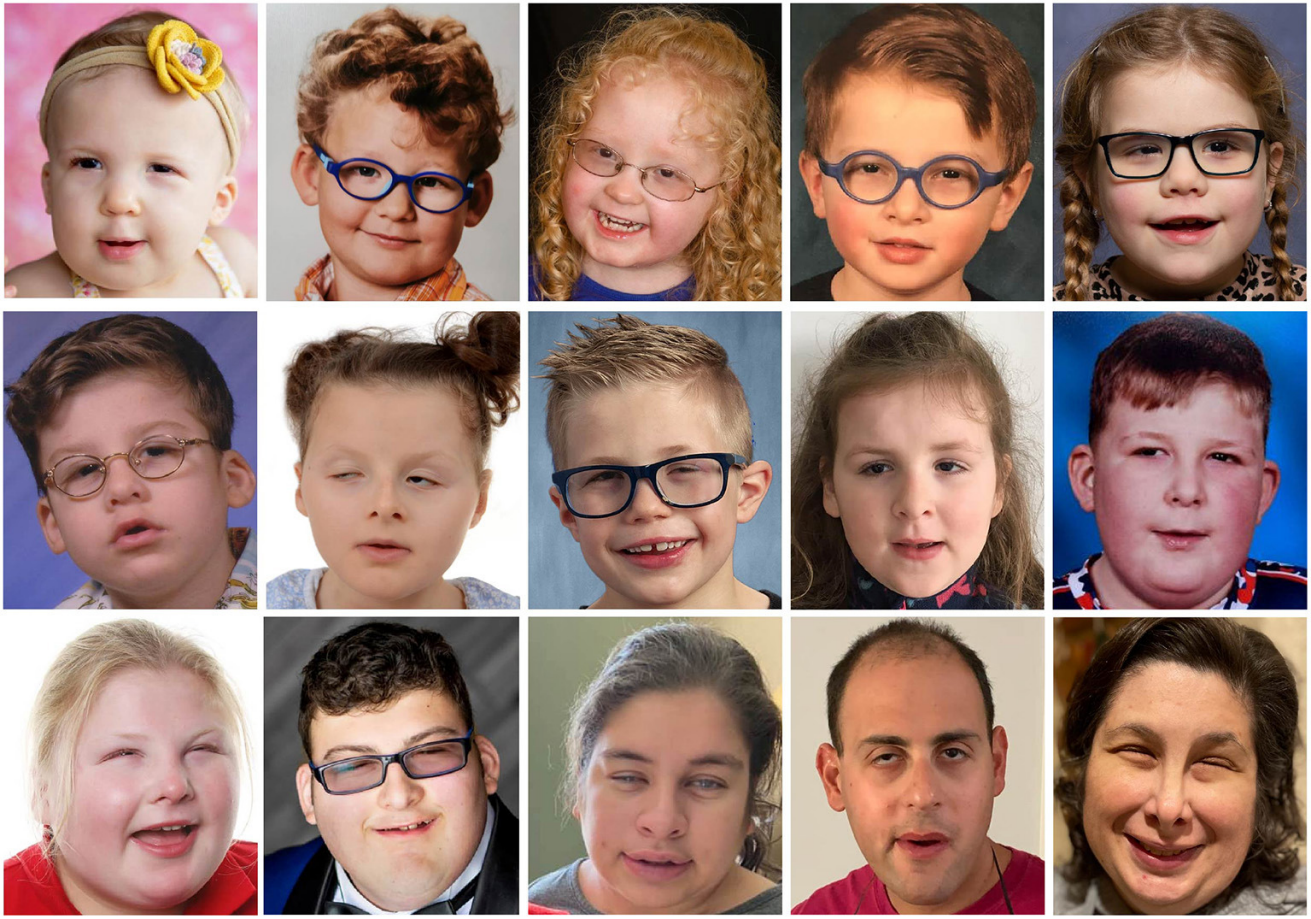
Facial images of individuals with WAGR spectrum. A variety of craniofacial appearances can present in patients, however similar appearances can be appreciated in some. Commonly observed dysmorphic features include low-set ears with abnormal shape; down-slant to palpebral fissures; and prognathism. Strabismus and amblyopia are common in addition to aniridia; and patients frequently wear glasses. Consent for publication was provided by parent or legal representative through the International WAGR Syndrome Association (IWSA).

Facial images of individuals with WAGR syndrome are shown in Figure 1. The cohort consisted of 52 females, 37 males, and two not reported. Birth country of the participants was provided by 85 individuals, with 16 countries/regions represented. The most common were the United States of America (*n* = 52) and United Kingdom (*n* = 10). At least one individual from every continent (except Antarctica) participated. The majority of participants were reported as white (*n* = 79); other races reported included Asian (*n* = 4); Black/African American (*n* = 1); and Mixed African-European (*n* = 1). Ethnicity was reported by 48 participants, with 10 identified as Hispanic or Latin American.

#### Molecular Abnormalities Reported

Almost all participants selected “genetic laboratory” as the source for their diagnosis; three reported “physical exam” indicating a clinical rather than molecular diagnosis. Participants reported specific genetic abnormalities by selecting checkboxes and a variety of genetic abnormalities were reported (Supplementary Table S1). Approximately 21% of participants selected the “Don't Know” checkbox. The “BDNF deletion” checkbox was selected by 27 of 54 valid participants, which represented a 50% frequency in the cohort. The “mosaic” checkbox was selected by three participants and six reported additional abnormalities beyond *WT1, PAX6*, and *BDNF* deletions (Supplementary Table S1). A deletion affecting the ELP4 gene was reported by two participants.

### Classic WAGR Features

The frequency of classic WAGR syndrome features in the cohort are shown in Table 1. The most common feature among the cohort was aniridia, present in all but two participants. The two participants not affected by aniridia reported amblyopia without other eye issues. One participant reported aniridia fibrosis syndrome.

**Table 1:** Frequency of classic WAGR features in the participants of the WAGR discovery cohort.

**Classic WAGR features**	**Total**	**Males**	**Females**
W **ilms tumor (WT) and/or nephrogenic rest(s) (NR)**			
**Total affected**	42/77 (54.5%)	18/30 (60.0%)	24/47 (51.0%)
Wilms tumor development	36/77 (46.8%)	13/30 (43.3%)	23/47 (48.9%)
Nephrogenic rest development only	6/77 (7.8%)	5/30 (16.7%)	1/47 (2.1%)
A **niridia**	81/83 (97.6%)	31/33 (93.9%)	50/50 (100%)
Aniridia fibrosis syndrome	1/57 (1.8%)	0/21	1/36 (2.8%)
G **enitourinary (GU) anomalies**			
**Total affected**	45/76 (59.2%)	28/33 (84.8%)	17/43 (39.5%)
Ambiguous genitalia	8/74 (10.8%)	7/31 (22.6%)	1/43 (2.3%)
Difficulty emptying bladder	7/70 (10.0%)	5/29 (17.2%)	2/41 (4.9%)
Ureteral duplication	4/66 (6.1%)	3/27 (11.1%)	1/39 (2.6%)
Large bladder	2/68 (2.9%)	1/28 (3.6%)	1/40 (2.5%)
Small bladder	1/67 (1.5%)	0/28	1/39 (2.6%)
**Congenital anomaly of the kidney and/or urinary tract (CAKUT)**	30/78 (38.5%)	17/32 (53.1%)	13/46 (28.3%)
**Recurrent urinary tract infections (UTIs)**	15/73 (20.5%)	5/31 (16.1%)	10/42 (23.8%)
**Male-related issues**			
Cryptorchidism	–	21/31 (67.7%)	–
Hypospadias	–	11/31 (35.5%)	–
Micropenis	–	7/28 (25.0%)	–
**Female-related issues**			
Streak ovaries	–	–	11/31 (35.5%)
Bicornuate uterus	–	–	5/34 (14.7%)
Polycystic ovarian syndrome (PCOS)	–	–	3/39 (7.7%)
Small uterus	–	–	2/32 (6.3%)
R **ange of cognitive, behavioral, and emotional issues**			
Cognitive and learning problems	69/78 (88.5%)	27/29 (93.1%)	42/49 (85.7%)
Behavioral problems	69/83 (83.1%)	29/35 (82.9%)	40/48 (83.3%)
Abnormal physical behaviors	50/82 (61.0%)	22/34 (64.7%)	28/48 (58.3%)
Behavioral/emotional conditions	37/76 (48.7%)	18/31 (58.1%)	19/45 (42.2%)
Psychiatric conditions	38/72 (58.2%)	16/30 (53.3%)	22/42 (52.4%)

*NR, nephrogenic rest(s); WT, Wilms tumor*.

#### Wilms Tumor Development

Approximately half of participants reported development of Wilms tumor (WT) and/or nephrogenic rests (NR) (Table 1). Six participants reported development of NR, but did not report WT (NR only). The majority of the cohort reported an early diagnosis of first WT or NR that developed: more than half were diagnosed by 18 months, more than three-quarters diagnosed before age 3 years, and 95% were diagnosed by age 5 years (Table 2). One participant reported an initial diagnosis of NR only with development of WT ~2.5 years later (Table 2, Highlighted Patient 1).

**Table 2:** Age at first development of Wilms tumor and/or nephrogenic rests in WAGR discovery cohort.

**Age at diagnosis of first tumor**	**Total diagnosed with tumor**	**Age at development of first tumor**		
		**Wilms tumor (WT)**	**Nephrogenic rest (NR) only**	**Cumulative incidence**
	**(*n* = 42)**	**(*n* = 36)**	**(*n* = 6)**	**(for WT or NR)**
Birth−1 year	13 (31.0%)	10	3	
0–6 months	4 (9.5%)	2	2	4 (9.5%)
7–12 months	9 (21.4%)	8^†^	1	13 (31.0%)
1 year−2 years	13 (31.0%)	12	1	
13–18 months	8 (19.1%)	7^†^	1	21 (50.0%)
19–24 months	5 (11.9%)	5^†^	–	26 (61.9%)
2 years−3 years	9 (21.4%)	7	2	35 (83.3%)
3 years−4 years	2 (4.8%)	2	–	37 (88.1%)
4 years−5 years	3 (7.1%)	3^‡^	–	40 (95.2%)
>5 years	2 (4.8%)	2	–	
5 years, 6 months		1	–	41 (97.6%)
7 years, 4 months		1	–	42 (100%)
	**Additional WT or relapse characteristics reported by participants**			
	**First diagnosis history reported**		**Age at additional detection**	
Highlighted patient 1	2 years old *(NR only, no WT)*		4 years, 5 months old *(WT, Stage III)*	
Highlighted patient 2	9 months old *(WT only, Stage IV)*		1 year, 8 months old	
Highlighted patient 3	11 months old *(WT + NR, Stage II)*		3 years old	
Highlighted patient 4	22 months old *(WT + NR, Stage V)*		19 years, 7 months old	

† *Relapse or additional tumor development at later age reported by one participant in each row*.

‡ *History of NR development at 2 years of age before the development of WT reported by one participant*.

*NR, nephrogenic rest(s); WT, Wilms tumor*.

Relapse was reported by three participants: one at 11 months after diagnosis (Table 2, Highlighted Patient 2); one at 25 months after diagnosis (Table 2, Highlighted Patient 3); and one more than 17 years after the first diagnosis (Table 2, Highlighted Patient 4). The latter participant reported a Stage 5 diagnosis at 22 months, with relapse/new tumor occurring at 19 years and 7 months and unfortunately died soon after. All other participants with WT and/or NR development were reported as alive at time of last survey completion. Other outcome data such as age since diagnosis were not available.

Among the 36 participants with WT, 41.7% also reported NR (*n* = 15). Tumor stage and histology information were provided by 32 participants. The majority of tumors were reported as Stage I (*n* = 18) or Stage II (*n* = 8); others included Stage III (*n* = 4), Stage IV (*n* = 1), Stage V (*n* = 1). All tumors were reported as favorable histology. The questionnaire did not ask participants whether tumors were diagnosed through routine surveillance.

#### Range of Cognitive and Behavioral/Emotional Issues

Participants were frequently affected by issues in the behavioral/emotional problems, cognitive/learning problems, and psychiatric conditions categories in the survey (Table 1), with a spectrum of characteristics and specific diagnoses reported by participants (Table 3). Approximately 77% of all participants reported cognitive impairment and/or global developmental delay and some participants did not report any severe/significant issues within the categories. These results confirm that a “Range of cognitive and behavioral/emotional issues” occur in this population.

**Table 3:** Frequency of issues in the “R” phenotype category reported by WAGR discovery cohort.

	**Total**	**Males**	**Females**
**Behavioral, emotional, and psychiatric characteristics**			
**Behavior problems**	**69/83 (83.1%)**	**29/35 (82.9%)**	**40/48 (83.3%)**
Temper tantrums	57/80 (71.3%)	25/33 (75.8%)	32/47 (68.1%)
Distractibility	50/73 (68.5%)	24/32 (75.0%)	26/41 (63.4%)
Impulsivity	44/73 (60.3%)	21/30 (70.0%)	23/43 (53.5%)
Hyperactivity	42/77 (54.5%)	21/32 (65.6%)	21/45 (46.7%)
Aggressiveness	39/74 (52.7%)	17/31 (54.8%)	22/43 (51.2%)
Food refusal	23/79 (29.1%)	8/34 (23.5%)	15/45 (33.3%)
**Physical behaviors**	**50/82 (61.0%)**	**22/34 (64.7%)**	**28/48 (58.3%)**
Spins, twirls, or paces	35/76 (46.1%)	15/31 (48.4%)	20/45 (44.4%)
Rocks body back and forth	32/78 (41.0%)	17/33 (51.5%)	15/45 (33.3%)
Need to line up items or make symmetrical	13/77 (16.9%)	9/32 (28.1%)	4/45 (8.9%)
**Conditions and co-morbidities**			
Behavior **AND** psychiatric disorder	26/63 (57.1%)	13/27 (48.1%)	13/36 (36.1%)
**NO** behavior or psych disorder	21/63 (33.3%)	7/27 (25.9%)	14/36 (38.9%)
Behavior **OR** psychiatric disorder	16/63 (23.9%)	7/27 (25.9%)	9/36 (25.0%)
**Conditions/disorders—behavioral**	**37/76 (48.7%)**	**18/31 (58.1%)**	**19/45 (42.2%)**
OCD *(obsessive compulsive disorder)*	20/67 (29.9%)	11/30 (37.6%)	9/37 (24.3%)
ASD *(autism spectrum disorder)*	19/76 (25.0%)	10/31 (32.3%)	9/45 (20.0%)
ADD/ADHD *(attention-deficit/hyperactivity)*	18/76 (23.7%)	8/31 (25.8%)	10/45 (22.2%)
Conduct disorder	12/63 (19.0%)	7/26 (26.9%)	5/37 (13.5%)
ODD *(oppositional defiance disorder)*	8/66 (12.1%)	5/29 (17.2%)	3/37 (8.1%)
**Conditions—emotional or psychiatric**	**38/72 (52.8%)**	**16/30 (53.3%)**	**22/42 (52.4%)**
Anxiety disorder	30/68 (44.1%)	11/28 (39.3%)	19/40 (47.5%)
Depression	8/66 (12.1%)	3/29 (10.3%)	5/37 (13.5%)
Panic attacks	7/65 (10.8%)	3/28 (10.7%)	4/37 (10.8%)
Bipolar/manic depression	2/65 (3.1%)	2/28 (7.1%)	0/37
**Delays, cognitive issues, and learning problems**			
**Cognitive and/or learning problems**	**69/78 (88.5%)**	**27/29 (93.0%)**	**42/49 (85.7%)**
Cognitive impairment	45/78 (57.7%)	20/29 (69.0%)	25/49 (51.0%)
Global developmental delay	44/78 (56.4%)	19/29 (65.5%)	25/49 (51.0%)
Learning disability in reading	2/785 (32.1%)	9/29 (31.0%)	16/49 (32.7%)
Learning disability in math	23/78 (29.5%)	9/29 (31.0%)	14/49 (26.8%)
Sensory integration disorder	17/78 (21.8%)	10/29 (34.5%)	7/49 (14.3%)
Pervasive developmental delay (PDD)	9/76 (11.8%)	7/31 (22.6%)	2/45 (4.4%)
Visual-perceptive disorder	1/78 (1.3%)	1/29 (3.4%)	0/49
Visual motor deficit	1/78 (1.3%)	1/29 (3.4%)	0/49
**Communication and/or speech issues**	**53/78 (67.9%)**	**22/29 (75.9%)**	**31/49 (63.3%)**
Speech (expressive) delay	49/78 (62.8%)	19/29 (65.5%)	30/49 (61.2%)
Language (receptive) delay	25/78 (32.1%)	13/29 (44.8%)	12/49 (24.5%)
Auditory processing disorder	17/78 (21.8%)	8/29 (27.6%)	9/49 (18.4%)
Social communication disorder	7/76 (9.2%)	3/31 (9.7%)	4/45 (8.9%)
Non-verbal learning disability	6/78 (7.7%)	4/29 (13.8%)	2/49 (4.1%)
Mutism	1/78 (1.3%)	0/29	1/49 (2.0%)

##### Behavior Problems, Physical Behaviors, and Learning Problems

Problems with behavior and learning were frequent in both males and females, and males were more commonly affected by some issues in the behavior and cognitive/learning categories (Table 3). A high rate of expressive speech and/or receptive language delays were observed in the population, and equal rates of reading and math learning problems were observed between males and females (Table 3). Temper tantrums and distractibility were among the most common behavioral problems, and males were more commonly affected by impulsivity and hyperactivity; equal rates of aggressiveness were observed, which was reported by approximately half of the cohort (Table 3).

##### Behavioral, Emotional, and Psychiatric Conditions

Close to half of participants reported at least one behavioral, emotional, or psychiatric diagnosis by a healthcare provider. Participants frequently reported comorbidities with at least one condition in both the behavior and the psychiatric conditions categories; ~25–33% of the cohort reported “no” or “none” for all conditions listed in the behavior and psychiatric diagnosis portion of the questionnaire (Table 3). All participants were commonly affected by attention-deficit/hyperactivity disorder (ADD/ADHD), panic attacks, and/or social communication disorder; with equal rates observed between males and females (Table 3).

Female participants were more commonly affected by anxiety disorder and depression; females were also more commonly affected by a psychiatric condition without a behavior condition (Table 3).

Male participants were more commonly affected by behavior conditions such as obsessive-compulsive disorder (OCD), autism spectrum disorder (ASD), and issues such as pervasive developmental disorder (PDD), conduct disorder, and oppositional defiance disorder (ODD). Males were also more commonly affected by a behavior condition without a psychiatric condition (Table 3).

##### Speech and Communication Issues

A spectrum of speech and communication disorders were reported by participants in addition to behavioral, emotional, and/or learning issues, with between 65 and 75% of the cohort affected by at least one communication issue (Table 3). The most common issues involved speech (expressive) and/or language (receptive) delays, and approximately one-fifth of the cohort was affected by auditory processing disorder (Table 3). Additional less common, but significant issues reported that could affect communication or speech included social communication disorder, non-verbal learning disability, and/or mutism/inability to speak (Table 3). These results suggest that a spectrum of communication issues and/or speech disorders can present within the “Range of cognitive and behavioral/emotional issues” in WAGR.

##### Comparison to Previous WAGR Studies

Similar rates of cognitive and behavioral issues were observed in our cohort compared to rates reported by previous WAGR studies (Table 4). Between 25 and 29% of patients are estimated to be affected by ADD/ADHD, anxiety, autism, and/or OCD (Table 4). The most common issues observed between three cohorts included cognitive delay, speech disorders, and developmental delays; speech disorders appear to be more prevalent within our cohort (Table 4).

**Table 4:** Range of behavioral, developmental, and emotional health issues in the WAGR patient population.

**Behavioral, developmental, and emotional issues**	**Frequency in WAGR population**				**Sex-specific frequencies**			
	**Total estimated frequency**	**Study frequencies**			**Male patients**		**Female patients**	
		**Fischbach et al.^a^**	**Xu et al.^b^**	**This study**	**Xu et al.^b^**	**This study**	**Xu et al.^b^**	**This study**
		**(*n* = 54)**	**(*n* = 31)**	**(*n* = 91)**	**(*n* = 14)**	**(*n* = 37)**	**(*n* = 17)**	**(*n* = 52)**
Cognitive impairment	68.4%	70%	77.4%	57.7%	92.9%	69.0%	64.7%	51.0%
Speech disorders	52.4%	NR	41.9%	62.8%	28.6%	65.5%	52.9%	61.2%
Developmental delay (DD)	45.9%	NR	35.5%	56.4%	28.6%	65.5%	41.2%	51.0%
Anxiety	29.0%	7.4%	35.5%	44.1%	21.4%	39.3%	47.1%	47.5%
Autism (ASD)	28.5%	18.5%	41.9%	25.0%	57.1%	32.3%	29.4%	20.0%
OCD	27.0%	9.3%	41.9%	29.9%	35.7%	36.7%	47.1%	24.3%
ADD/ADHD	25.6%	24.1%	29.0%	23.7%	21.4%	25.8%	35.3%	22.2%
Pervasive DD (PDD)	8.8%	5.6%	6.5%	14.3%	14.3%	22.6%	0%	4.4%
Depression	8.1%	5.6%	6.5%	12.1%	0%	10.4%	11.8%	13.5%

*Total Estimated Frequency is the average of the percentages observed for each study, as applicable. Percentages listed for this study are based on the number of participants affected compared to the number who answered relevant question; for literature series, percentages are based on the number of affected patients compared to the total number in cohort*.

*ADD/ADHD, attention-deficit/hyperactivity disorder; ASD, autism spectrum disorder; DD, developmental delay (for the present study, defined as global developmental delay); NR, not reported; OCD, obsessive compulsive disorder; PDD, pervasive developmental disorder*.

a *Fischbach et al. (1)*.

b *Xu et al. (5)*.

#### Genitourinary and Kidney Anomalies

More than half of the cohort was affected by a GU anomaly, with males affected at twice the rate of females (Table 1). Disorders of sexual differentiation *(“ambiguous genitalia” as survey variable)* were reported more often by males. Difficulty emptying the bladder was the most common issue affecting both sexes. Female participants commonly reported streak ovaries and males frequently reported cryptorchidism (Table 1). Internal GU anomalies were reported by 34.1% of females (*n* = 14/41).

More than a third of participants reported an issue that was consistent with a congenital anomaly of the kidney and/or urinary tract (CAKUT) (Table 1). In addition to genital and urinary tract anomalies, approximately half of the cohort reported being affected by at least one issue within the “renal conditions” (kidney) category, with the most common listed in Table 5 and a variety of additional issues listed in Supplementary Table S2. Recurrent infections of the urinary tract (UTIs) were common in the cohort, and females tended to be affected slightly more often than males (Table 1). A correlation between CAKUT and UTIs appeared to exist in the cohort: among the 15 with recurrent UTIs, 9 (60.0%) had reported an issue consistent with CAKUT. Recurrent infections were reported by 5/16 males and by 4/10 females with CAKUT, representing recurrent UTI rates associated with CAKUT in 31.3% of male and 40.0% of female participants.

**Table 5:** Common health issues affecting participants in the WAGR discovery cohort.

**Health category/issue**	**Participants affected**	**Frequency (%)**
**Cardiometabolic Health**		
**Congenital heart defects**	16/71	22.5%
Structural heart defects	11/70	15.7%
**Endocrine/metabolic issues**	58/81	71.6%
Obesity	39/74	52.7%
Short stature	33/69	47.8%
**Heart disease**	44/70	62.9%
Hypertension	23/65	35.4%
Heart murmur	20/60	29.0%
Hyperlipidemia	17/67	25.4%
Positive criteria for potential metabolic syndrome^*^	8/68	11.8%
**Kidney/Renal Issues**	39/76	51.3%
Recurrent urinary tract infections (UTIs)	15/73	20.5%
Vesicoureteral reflux (VUR)	7/71	9.9%
**Chronic kidney disease (CKD) features**	28/73	38.4%
Proteinuria	24/72	33.3%
Kidney failure	17/68	25.0%
Focal segmental glomerulosclerosis (FSGS)	14/72	19.4%
**Gastrointestinal (GI) Issues**	61/80	76.3%
Chronic constipation	41/78	52.6%
Feeding problems	38/79	48.1%
Gastroesophageal reflux disease (GERD)	27/75	36.0%
Chronic diarrhea	13/77	16.9%
Dysphagia	12/74	16.2%
**Respiratory Issues (Lung/Breathing)**	65/77	84.4%
Apnea	29/69	42.0%
Obstructive sleep apnea (OSA)	22/73	30.1%
Asthma	18/71	25.4%
Shallow breathing	16/69	23.2%
Shortness of breath	13/69	18.8%
Reactive airway syndrome/pre-asthma	7/66	10.6%
**Frequent illness** (colds and/or pneumonia)	45/71	63.4%
Respiratory tract infections requiring antibiotics	45/73	61.6%
Frequent colds (>7–8 per year)	41/70	58.6%
Pneumonia	37/74	50.0%
Frequent pneumonia (2 or more in a year)	22/73	30.1%
**Neurological Problems**	28/74	37.8%
Seizures	12/66	18.1%
Pseudobulbar affect	9/65	13.8%
Migraine	6/63	9.5%
**Abnormal Muscle Control/Tone**	53/77	68.8%
Hypotonia	38/68	55.9%
Spasticity	24/69	34.9%
Hypertonia	17/69	24.6%
Ataxia	15/62	24.2%
**Musculoskeletal Problems**	62/78	79.5%
Toe-walking	37/75	49.3%
Scoliosis	14/71	19.7%
**Hand/foot conditions**	40/76	52.6%
Flat feet	18/76	23.7%
Small feet/toes	10/76	13.2%

* *Participants considered positive criteria for potential metabolic syndrome if obesity was reported in addition to at least two adverse cardiometabolic features (hypertension, hyperlipidemia, and/or glucose intolerance)*.

#### Additional Issues Affecting the Eyes

All participants reported at least one issue within the ocular category and a variety of different issues were reported (Table 6). The most common issues included nystagmus, cataract(s), glaucoma, and/or myopia; a variety of other issues were reported (Table 6).

**Table 6:** Health issues affecting the eyes, ears, nose, throat, and mouth in the WAGR discovery cohort.

**Health category/issue**	**Participants affected**	**Frequency (%)**
**Eye Issues** (total affected)	85/85	100%
**Common eye issues**		
Nystagmus	77/82	93.9%
Cataract(s)	68/79	86.1%
Glaucoma	41/75	55.7%
Myopia	29/60	48.3%
Optic nerve hypoplasia	24/59	40.7%
Foveal/macular hypoplasia	22/58	37.9%
Amblyopia	21/74	28.4%
Strabismus	18/68	26.5%
Corneal keratopathy/pannus	17/70	24.3%
Hyperopia	14/60	23.3%
Peter's anomaly	13/67	19.4%
**Less common eye issues**		
Retinal detachment	8/74	10.8%
Aphakia	8/76	10.5%
Coloboma	7/69	10.1%
Anisocoria	3/68	4.4%
Heterochromia	3/76	3.9%
Microcornea	1/66	1.5%
Microphthalmos	1/68	1.5%
Anophthalmus	1/76	1.3%
**Allergy Problems**	50/84	59.5%
Atopic dermatitis/eczema	19/84	22.6%
Medication allergies	17/84	20.2%
Hay fever/allergic rhinitis	11/84	13.1%
Lactose intolerance	10/84	11.9%
Food allergies	9/84	11.7%
**Dental Conditions**	36/68	52.9%
High arched palate	18/58	31.0%
Crowded teeth	14/68	20.6%
Tooth/teeth extraction	14/68	20.6%
Small teeth	10/68	14.7%
Large gap between top two front teeth	9/68	13.2%
Weak/soft tooth enamel	8/68	11.8%
Malocclusion	7/68	10.3%
**ENT Procedures (Ear, Nose Throat)**	52/81	64.2%
Adenoidectomy	41/80	51.2%
Tympanostomy tubes	35/78	44.9%
Tonsillectomy	35/79	44.3%
**Hearing Loss**	11/75	14.7%
Use of hearing aids	4/76	5.3%
**Sleep Problems**	47/75	62.7%
Less than usual for age	36/71	50.7%
More than usual for age	12/71	16.9%

### Health Issues in WAGR Discovery Cohort

**Figure 2:**
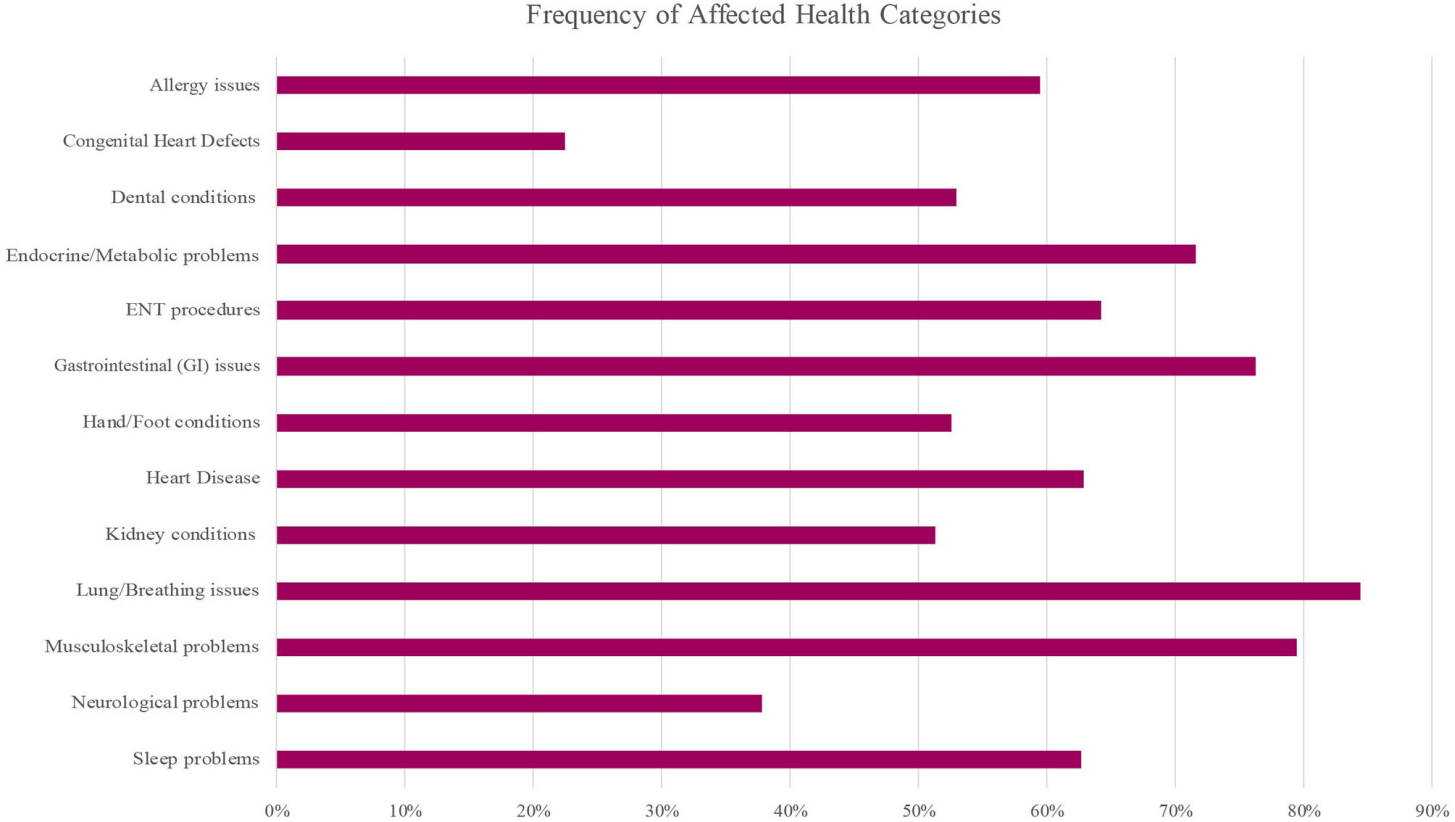
Frequency of health issue categories affected in the WAGR Discovery Cohort. A variety of health categories were frequently affected. Approximately 75% or more of participants were affected by at least one issue in the Gastrointestinal (GI), Respiratory (lung/breathing), and Musculoskeletal categories. More than half of participants were affected by at least one issue in the Allergy, Dental, Endocrine/Metabolic, Heart Disease, Kidney conditions, Hand/Foot conditions and Sleep problems categories; ENT procedures were frequent. ENT, ear, nose, and throat; GI, gastrointestinal.

Participants were frequently affected by the majority of the health categories (Figure 2). A number of common health issues were observed and those with ≥10% prevalence within the cohort are listed in Tables 5, 6, with additional health issues reported by participants available in Supplementary Tables S2, S3.

#### Common Health Issues

Respiratory issues (Lung/Breathing category) were frequent, and more than 60% of participants reported frequent illnesses; additional common issues included apnea, asthma, and obstructive sleep apnea (OSA) (Table 5). Musculoskeletal problems and conditions affecting the hands or feet were common (Table 5), with a variety of specific issues reported (Supplementary Table S3). More than 75% of the cohort was affected by at least one gastrointestinal (GI) condition, with approximately half reporting chronic constipation and/or feeding problems in addition to other common GI issues (Table 5) and less common issues (Supplementary Table S2). Although less common compared to some other clinical issues, we observed pancreatitis reported by 5 of 69 participants (7.2% of cohort).

Hearing loss was reported by close to 15% of participants, and sleep problems were common (Table 6). A number of problems affecting the ears, nose, throat (ENT) and/or mouth were frequently reported (Table 6), with a variety of additional specific issues that were less common (Supplementary Table S3). The most common problems included allergy issues, ENT procedures, and issues affecting the teeth; close to a third reported a high arched palate (Table 6).

#### Neurological Problems

Approximately two-thirds of the cohort was affected by abnormal muscle control or tone, and more than a third of the cohort was affected by at least one neurological problem (Table 5). Frequency of epilepsy was not asked, although one participant reported epilepsy within the free-text portion of the questionnaire.

Participants were asked which neurology tests had been performed and the overall result (normal/abnormal/unknown) for each test. Neurology testing was reported by 50 participants, and 27 (54%) reported at least one abnormal result. Brain CT (computed tomography) was abnormal in 50%; brain MRI (magnetic resonance imaging) was abnormal in 48%; and abnormal EEG (electroencephalography) was reported by ~39% of the cohort.

#### Cardiometabolic Health

High rates of cardiometabolic issues were reported by participants and hypertension was present in more than a third of the cohort (Table 5). Congenital heart defects were present in approximately 20% of participants and 15% were affected by a structural defect, however no specific type of cardiac defect was found to commonly occur (Supplementary Table S2). Among the 16 patients with a congenital heart defect, four were premature.

Obesity and short stature were the most common endocrine/metabolic issues reported (Table 5) and were commonly reported together. Among the 26 participants with a reported BDNF deletion, close to two-thirds reported obesity (*n* = 17) and close to a third (*n* = 7/22) reported obesity with short stature. A variety of other endocrine/metabolic issues were reported (Supplementary Table S2).

##### Criteria for Potential Metabolic Syndrome

Given the constellation of common cardiometabolic features, we hypothesized that participants may be affected by potential metabolic syndrome criteria. Those with reported obesity and at least two other features (hyperlipidemia, hypertension, and/or glucose intolerance) were considered positive; those with obesity and less than two features, and those without obesity, were considered as negative; participants with missing data related to criteria were excluded. An approximate rate of 12% of participants with potential metabolic syndrome was observed (Table 5).

#### Chronic Kidney Disease

**Figure 3:**
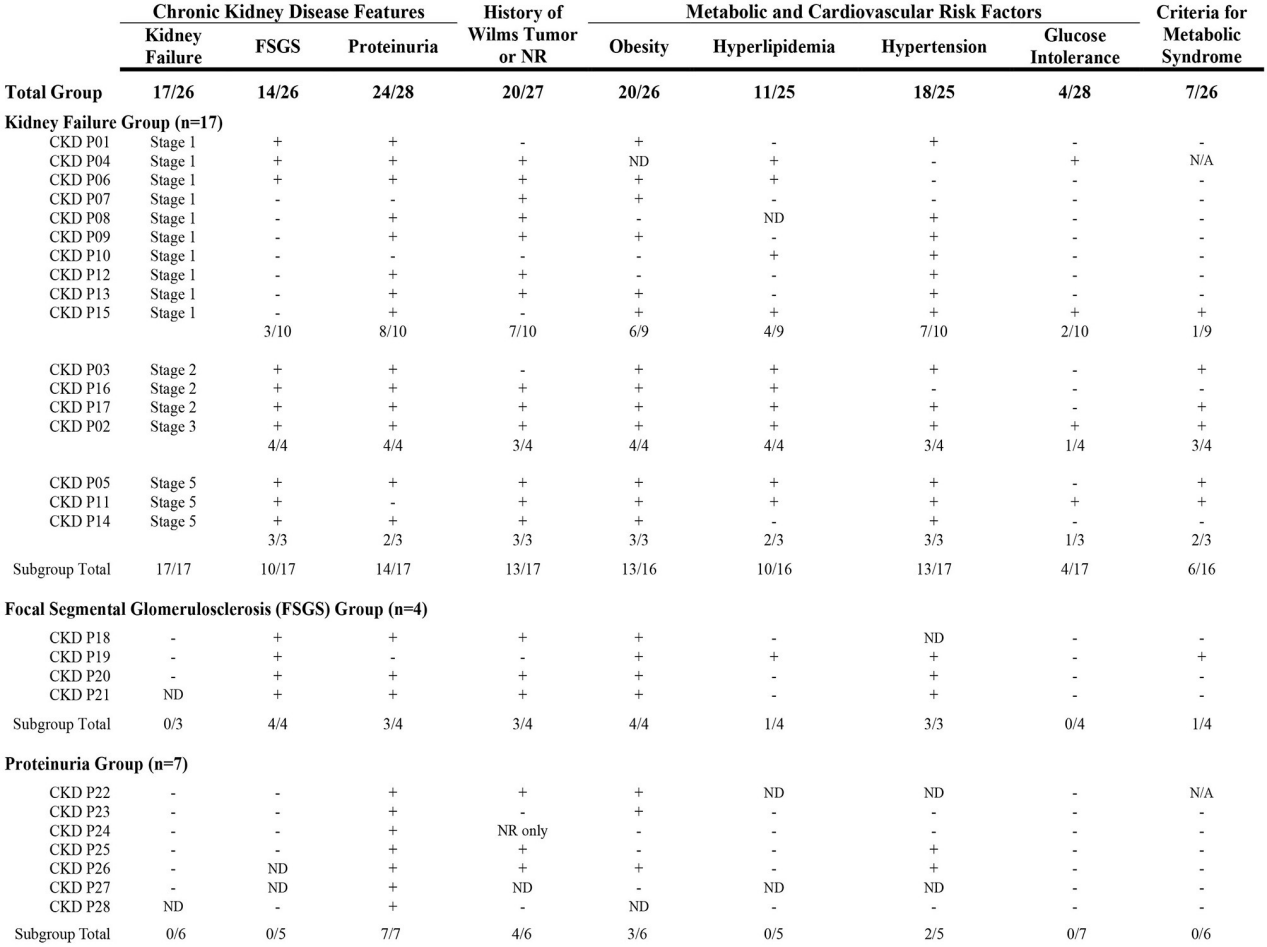
Clinical characteristics reported by participants affected by Chronic Kidney Disease (CKD). A spectrum in the degree of CKD features was observed and included kidney failure, focal segmental glomerulosclerosis (FSGS), and/or proteinuria. Participants are shown in subgroups based on CKD features reported. The presence of clinical characteristics in each participant are shown as: **+**(affected); –(not affected); or ND (no data). Seven participants did not have a history of Wilms tumor or nephrogenic rest (NR). Cardiometabolic features were common and ~75% of the entire group was affected by obesity and/or hypertension. More than a third of participants in the kidney failure group were affected by criteria for metabolic syndrome [defined as obesity and at least two other features (hyperlipidemia, hypertension, and/or glucose intolerance)]. CKD, chronic kidney disease; FSGS, focal segmental glomerulosclerosis; ND, no data *(participant did not provide response to specific question)*; N/A, not applicable *(missing data)*; NR, nephrogenic rest(s).

Participants were frequently affected by features of chronic kidney disease (CKD), with rates of proteinuria, kidney failure, and/or focal segmental glomerulosclerosis (FSGS) reported between 20 and 33% in the total cohort (Table 5). No participants reported mesangial sclerosis. At least one feature of CKD was reported by 28 individual participants, with 42.9% of females (*n* = 18/42) and 32.3% of males (*n* = 10/31) affected. A range in degree of CKD features that affected each participant was observed: 17 participants reported kidney failure with or without FSGS (most in Stage 1 or 2); four reported FSGS without kidney failure; and seven reported isolated proteinuria (Figure 3). Cardiometabolic features were common, and history of WT or NR development was reported by 76% of participants with kidney failure and/or FSGS, and two thirds of participants with isolated proteinuria.

A gradient in the degree of CKD and presence of cardiometabolic features was observed (Figure 3). Participants with isolated proteinuria were observed to have lower rates of features compared to those with kidney failure and/or FSGS. Participants who reported kidney failure in Stages 2 to 5 also appeared to have higher frequencies of cardiometabolic features compared to the group with Stage 1 failure. More than 25% of the participants with CKD had positive criteria for metabolic syndrome (Figure 3). These results suggest that adverse cardiometabolic profiles are likely correlated to severity of CKD issues and kidney failure stage.

##### Chronic Kidney Disease Without History of Wilms Tumor

Among those with kidney failure and/or FSGS, five participants (24%) did not report history of WT or NR development *(CKD ID#* = *P01, P10, P15, P03, P19)*. Cardiometabolic issues were frequently reported by these participants (Figure 3): hypertension was present in all five, four reported obesity and/or hyperlipidemia, and one reported glucose intolerance; three of five (60%) had features suggestive of metabolic syndrome.

### Birth Characteristics

The majority of participants were naturally conceived, singletons, and were not premature; among the full-term participants, most reported normal birth length and weight (Table 7). A variety of checkboxes related to problems present at birth were selected by participants and/or provided by free-text responses. Craniofacial abnormalities were frequent and almost half of participants reported ear abnormalities (Table 7). A variety of other problems at birth were reported, but most were limited to one or two participants and did not fit into specific categories.

**Table 7:** Prenatal and birth characteristics in the WAGR discovery cohort.

	**Participants affected**	**Frequency (%)**
**Prenatal and birth characteristics**		
**Conception type**		
Natural	79/86	91.9%
Assisted (IVF/ART)	7/86	8.1%
**Multiple gestation pregnancy**	5/86	5.8%
Natural conception	2/79	2.5%
IVF/ART conception	3/7	42.9%
**Preterm birth (<37 weeks)**	11/81	13.6%
**Additional characteristics (full term cohort)**		
**Birth length**		
**Small** *(<18 inches or 46 cm)*	7/60	11.7%
**Average** *(between 18 and 22 inches or 46–56 cm)*	52/60	86.7%
**Large** *(more than 22 inches or 56 cm)*	1/60	1.7%
**Birth Weight** *[1 pound (lb) = 0.454 kg]*		
**4 lbs−4.9 lbs** *(or 1.8 kg−2.2 kg)*	5/69	7.2%
**5 lbs−5.9 lbs** *(or 2.3 kg−2.6 kg)*	13/69	18.8%
**6 lbs−6.9 lbs** *(or 2.7 kg−3.0 kg)*	21/69	30.4%
**7 lbs−7.9 lbs** *(or 3.1 kg−3.5 kg)*	22/69	31.9%
**8 lbs−8.9 lbs** *(or 3.6 kg−4.0 kg)*	8/69	11.6%
**Problems present at birth**		
Craniofacial abnormalities	39	72.2%
Palate abnormalities	10	18.5%
Ear abnormalities	26	48.1%
Abnormal pinna shape/fold	15	27.8%
Low-set ears	14	25.9%
Skull abnormalities	15	27.8%
Microcephaly	10	18.5%
Enlarged parietal foramina	5	9.3%
Brain abnormalities	8	14.8%
Agenesis of the corpus callosum	6	11.1%
Respiratory system underdevelopment	7	13.0%
Laryngomalacia	3	5.6%
Bronchomalacia	2	3.7%
Tracheomalacia	1	1.9%
Pharyngomalacia	1	1.9%

^*^Estimated frequency is proportion of number of participants reporting issue or category compared to total participants with valid completion of “birth problems” question (n = 54). cm, centimeter; in, inch; IVF/ART, use of in-vitro fertilization or other assisted reproduction technology for conception; lbs, pounds.

### Psychosocial and Well-Being Characteristics

Half of participants reported use of least one assistive device from the checkbox options, and almost all participants reported receiving at least one therapy type (Table 8). The majority of participants were reported as dependent and a student or unemployed (Table 8). Participants ≥18 years were asked to report the highest level of education achieved, and the majority of participants completed high school (Table 8). Four participants reported additional education after high school (three attending trade/business/vocational school; and one completing some college without graduating). One participant completed trade/business/vocational education as an alternative to high school.

**Table 8:** Psychosocial and well-being characteristics in the WAGR discovery cohort.

	**Participants affected**	**Frequency (%)**
**Supportive Devices**		
**Use of at least one assistive device**	39/76	51.3%
White cane for visually impaired	25/76	32.9%
Orthotics	17/76	22.4%
Manual wheelchair	8/76	10.5%
Continuous positive airway pressure/CPAP	6/76	7.9%
Hearing aids	4/76	5.3%
Walker	3/76	4.0%
Intelligent imaging device	1/76	1.3%
Service dog	1/76	1.3%
**Therapies**		
**Use of at least one therapy type**	80/81	98.8%
Occupational Therapy (OT)	70/79	88.6%
Physical Therapy (PT)	66/80	82.5%
Speech therapy	67/77	87.0%
**Emerging trends in therapy use**		
Nutrition therapy	4/10	40.0%
Complementary therapy (vitamin, herbal, or nutrition supplements)	4/10	40.0%
Psychological therapy	2/7	28.6%
Rehabilitation therapy	1/6	16.7%
**Psychosocial/Well-Being**		
**Current living situation: dependent status**	80/82	97.6%
Living with parent/relative	73/82	89.0%
Group home or skilled care facility	7/82	8.5%
**Employment status**		
Student	40/70	57.1%
Employed (part-time)	5/70	7.1%
Employed (full-time)	2/70	2.9%
Unemployed	23/70	32.9%
**Educational achievement (>18 years)**		
High School (HS) diploma or equivalent	15/21	71.4%
Did not graduate HS	6/21	28.6%
**Completed additional education after HS**	4/21	19.1%

### Historical Review of the Evolution of the WAGR Phenotype–80 Years of Evidence

We compiled the evidence from cohorts of patients with WAGR starting with the information from the first description of aniridia-WT association published in 1964 by Miller et al. (6) through evidence recently published in 2021 by Hol et al. (7) and compared trends in clinical issues reported across selected studies to the frequencies reported by the WAGR Discovery Cohort. As the data collection for the 1964 study began in January 1940 (6) and the data collected from the WAGR Syndrome Patient Registry ended in May 2020, we have created an 80 year history of the evolution of the full spectrum of phenotypes and clinical issues that commonly affect this population. The full results of these comparisons are available in Supplementary Appendix, including a description of the selected studies, summaries of common clinical issues by system, and proposed application to the current WAGR patient population.

Many of the clinical issues characterized in the WAGR Discovery Cohort have been described in at least one or two patients previously, and we observed comparative rates or increased rates of features across the selected studies (Supplementary Appendix). The risk for internal GU anomalies, CKD development, and obesity in addition to the classic WAGR features was confirmed; additionally, it appears that the presence of CAKUT has been underappreciated in the past (Supplementary Appendix). The presence of various cardiac anomalies has been described in patients and it appears that structural defects may be more common than previously appreciated (Supplementary Appendix). Short stature appears frequent, and a substantial portion of patients may have lower birth weights and/or microcephaly. Emerging findings in the population included: craniofacial features; frequent illness and need for ENT procedures; pulmonary or respiratory issues; and neurological and/or musculoskeletal abnormalities (Supplemental Appendix). It does not appear that other studies evaluated or identified a risk for recurrent urinary tract infections (UTIs), which was observed at a high frequency in the WAGR Discovery Cohort. Early characterization suggested a male predominance, however more recent studies have included more females, providing evidence that both males and females can be affected by the features of WAGR (Supplementary Appendix).

#### The Gastrointestinal System as an Emerging Phenotype of WAGR Spectrum

From the historical literature evidence perspective, gastrologic findings are less well-characterized but also appear common and can range from milder issues such as GERD to more serious complications such as pancreatitis (Supplementary Appendix). A high rate of issues in the GI category were reported by the WAGR Discovery Cohort and we have summarized these findings on pages 19–20 of the Appendix. Although no comparative data are available to correlate findings, the International WAGR Syndrome Association (IWSA) has provided anecdotal evidence that GI findings have been a common concern within the WAGR community for decades and the GI characteristics observed within the present study are generalizable to the population. Further data and characterization of these issues should be prioritized for future studies in WAGR spectrum populations.

## Discussion

The results of the WAGR Discovery Cohort are consistent with those reported by Fischbach, Trout (1) and demonstrate that clinical issues far exceed the classic features that historically have characterized WAGR syndrome. We propose the concept of WAGR spectrum to acknowledge the wide range of clinical manifestations that can present in affected patients and discuss common issues that characterize this disorder. A comprehensive multidisciplinary team approach is needed to support patients affected by WAGR spectrum and we propose a care model with general recommendations for all patients, and additionally highlight specialists that may be needed for individualized care.

Standard criteria for WAGR diagnosis has included the presence of aniridia and at least one of the three other classic features (1). A variety of combinations of the classic W-A-G-R features and molecular abnormalities have been reported in the literature, leading to multiple different terms to classify patient subgroups. The AGR syndrome/triad has previously been used to describe patients without WT development (8, 9). To describe the association with obesity, WAGR plus (10) and WAGRO (11) have been proposed. WAG(r) syndrome has been proposed due to the lack of significant developmental delay in some patients (12). Earlier reports referred to patients with del 11p/aniridia complex and del 11p13 (13). Although some unique differences appear to exist within these subclassifications, patients share a common underlying genetic background of chromosome 11p13 haploinsufficiency that likely lead to the range of clinical manifestations observed.

Focusing on the main associations of the WAGR phenotype may have led to under-characterization of the disorder and our results suggest that a number of additional systems are involved. These features are common in addition to the classic features, and build upon the previously expanded phenotype reported by Fischbach, Trout (1). These observations prompt the reclassification of the disorder from a syndrome to a spectrum in an effort to describe the wide variety of clinical manifestations that may affect patients.

### WAGR Spectrum Disorder

The range of clinical issues caused by the same molecular region of chromosome 11p13 can be characterized within the concept of “**WAGR spectrum disorder**,” which serves as an umbrella term to describe the various clinical and molecular subgroups and their presentations. The concept of a spectrum disorder rather than syndrome disorder will hopefully allow for a broader view of this disorder and pave the way for future investigations beyond the classic W-A-G-R manifestations that have historically been evaluated. Two separate spectrums could be used to characterize patients: the clinical spectrum to describe the frequency and combinations of features; and the molecular spectrum to describe subgroups of patients affected by specific genes within the 11p13 region. In the future, these spectrums could serve to help establish genotype-phenotype associations within the full WAGR spectrum. For the purposes of consistency, we will now refer to all previously reported patients and Discovery Cohort participants as being affected by WAGR spectrum disorder.

#### Characterization of WAGR Spectrum Phenotype

In the initial conceptualization of WAGR spectrum, the clinical phenotype can be described by Classic features, Common features, and Isolated features. Clinical presentations could be considered as “classic WAGR” to describe those with the classic and common features and/or 11p13 deletions, as well as “atypical WAGR” to describe those with 11p13 deletions without the classic phenotype. As the phenotype of WAGR spectrum expands, further cohort studies could help delineate which features are more prevalent than others among patients to characterize specific features into groups (classic, common, isolated, etc.).

We utilize observations from the Discovery Cohort to initially characterize the phenotype of WAGR spectrum and describe the classic features of the disorder in addition to other considerations. This cohort represents the largest group of patients affected by WAGR ever reported.

##### WAGR Spectrum Criteria

The diagnosis of WAGR spectrum disorder can be considered the combination of clinical WAGR features and molecular abnormalities consistent with 11p13 deletion. We propose the initial spectrum as ranging from the presentation of isolated aniridia to classic WAGR features and/or molecular diagnosis of 11p13 deletion in the WAGR region.

##### Reconceptualization of Classic WAGR Features

The “classic” features of W-A-G-R can represent phenotypic spectrums within each respective letter, rather than the clinical issue that defines them. Our findings confirm the approximate 50% risk of WT within the WAGR spectrum population. We observed multiple participants with reported nephrogenic rest development but without development of WT, suggesting that the “W” may be better defined as “Wilms tumor or WT precursor risk” in order to highlight this important issue among all patients. Aniridia may be better defined as “Aniridia and other ocular issues” in order to highlight the wide range of common ocular issues in this population. Genitourinary anomalies (internal and external) are frequent, and we observed a number of participants affected by congenital anomalies of the kidney and urinary tract (CAKUT), which has not previously been associated with the disorder. As a result it may be more appropriate to conceptualize “G” as “Genital and nephro-urological anomalies” to describe the spectrum of issues that can present and highlight the potential specialists needed to manage care. The “R” that defines WAGR has previously been reconceptualized from mental retardation to “Range of developmental delays” and our findings support this designation; the term “range of neurodevelopmental issues” could also be considered.

##### Frequency of Classic WAGR Features in Syndrome and Spectrum

**Figure 4:**
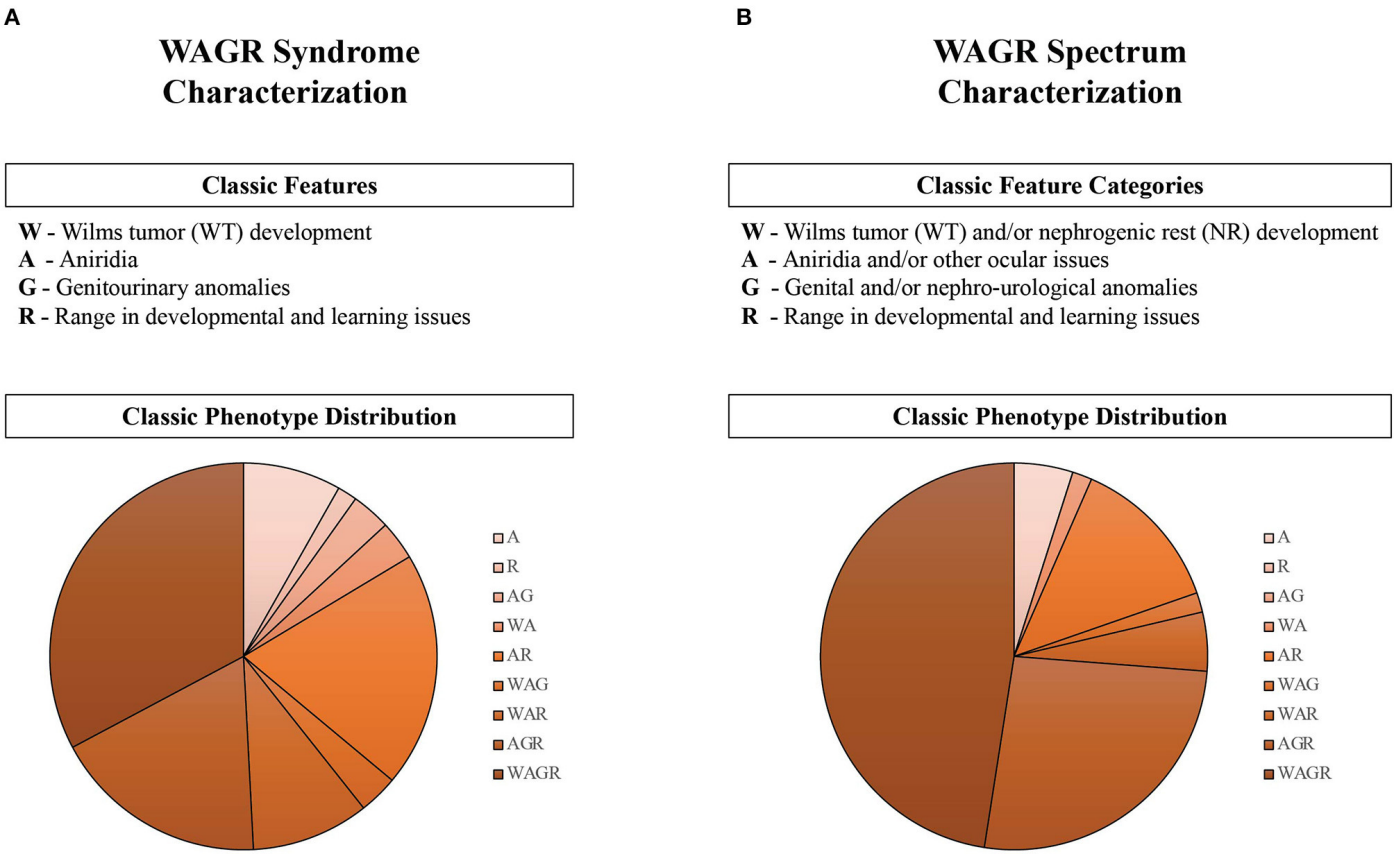
Characterization of classic features and phenotypes in the WAGR Discovery Cohort (*n* = 61). The criteria for classic features of the disorder are described for **(A)** WAGR Syndrome, as historically associated; and **(B)** WAGR Spectrum, as initially conceptualized. Phenotypes of participants are depicted in the pie charts, and describe the constellation of classic features reported by each individual (summarized by abbreviation subgroups). A spectrum of classic phenotype distributions were observed, and more phenotype subgroups were observed in WAGR Syndrome compared to WAGR Spectrum. Reclassification of “G” increased the number of participants affected by this classic feature by 18% between WAGR Syndrome (59.2%) and WAGR Spectrum (77.2%). Three main feature constellations were present in WAGR Spectrum: “WAGR,” “AGR,” and “AR” phenotypes. Close to half of participants had features consistent with the full WAGR phenotype *(all four features)* in the WAGR Spectrum characterization compared to approximately one third in the WAGR Syndrome characterization. Applying historical WAGR diagnostic criteria *(aniridia in the presence of one or more other features)*: WAGR Syndrome criteria was met in 90% of participants (*n* = 55), while WAGR Spectrum criteria was met in 95% of participants (*n* = 58).

We characterized WAGR syndrome and WAGR spectrum criteria in 61 participants with information available for all relevant data (Figure 4). Syndrome criteria included the standard definitions for the four classic features that define the disorder (WAGR) (Figure 4A). Spectrum criteria included a broadened concept of the four classic features to categories rather than the standard definition (Figure 4B). Higher rates for all classic features were observed through the spectrum characterization and 100% of participants were characterized as affected by the “Aniridia or ocular issues” category. The rate of participants affected by “G” increased by 18% when characterized through the spectrum (Figure 4). One participant was only affected by one classic syndrome feature (R- range of developmental delays), however was found to have three issues characterized as WAGR spectrum features (AGR phenotype: A-amblyopia(ocular); G-duplicate kidney; and R).

A variety of combinations of features and phenotypes were observed (Figure 4). One third of participants had full WAGR phenotypes (four features) based on syndrome criteria (Figure 4A), however close to half of participants had full WAGR phenotypes characterized through spectrum classifications (Figure 4B).

#### Considerations for WAGR Spectrum

WAGR spectrum criteria that includes isolated aniridia, or a combination of aniridia or ocular issues and other classic WAGR features allows for classification of 100% of the WAGR Discovery Cohort. Previous criteria for WAGR syndrome that includes aniridia and one or more other classic features classified between 90 and 95% of participants (Figure 4).

##### Atypical and Isolated Presentations

A small subset of participants were only affected by aniridia, which could be considered “isolated aniridia” or an “isolated feature” of the WAGR spectrum phenotype in the initial characterization. While aniridia is present in the large majority of patients, examples of patients without aniridia affected by WAGR have previously been reported (1) and we observed two participants without aniridia but affected by an ocular issue in our cohort. Lack of aniridia has been described in both female and male patients with WAGR (Supplementary Appendix). These patients may represent “atypical” presentations of WAGR spectrum; or could represent the full spectrum of ocular issues that appear to affect patients. Further characterization of WAGR spectrum could help delineate the frequencies of patients with WAGR who present with an isolated feature and those without aniridia to better describe these phenotypes.

##### Common Features in WAGR Spectrum

The classic features of WAGR spectrum can be considered those that characterize the disorder, however the results of the WAGR Discovery Cohort suggest that a number of other features and clinical issues frequently affect patients, termed “common features” for WAGR Spectrum disorder. We observed high rates of obesity, hypertension, and chronic kidney disease (CKD), in addition to a variety of cardiac, pulmonary, and other health issues. Given these associations, it can be considered that patients with WAGR spectrum share an increased risk for adverse cardiometabolic health and CKD in addition to WT development, and this notion is an important concept within the WAGR spectrum. Specific considerations for common health issues are discussed below.

As commented above, it is likely that this phenotype may change as more evidence becomes available regarding WAGR spectrum characteristics. Further phenotypic investigations into WAGR spectrum can help determine which features are more frequent than others to better define categorical features of the spectrum.

##### Common Craniofacial Characteristics

We observed a high rate of reported ear and other craniofacial abnormalities, suggesting that a craniofacial phenotype may exist within WAGR spectrum. It has previously been reported that no characteristic facial dysmorphism is associated with WAGR syndrome (13), and this has not been evaluated in more recent cohorts. Interestingly, in the first clinical association of WAGR, Miller et al. described children with aniridia, WT, and pinna abnormalities (6) that were similar to those reported by participants in our study. Microcephaly and/or head circumference ≤ 2 SDs below the mean was also previously reported in association with aniridia and WT (6) and we observed a number of participants with reported microcephaly at birth in the present study. More recent studies have described other jaw or dental issues that were also commonly reported by the WAGR Discovery Cohort (Supplementary Appendix). As the phenotype of WAGR spectrum is characterized, it is likely that a gradient in the degree of craniofacial dysmorphism exists between patients, however common features can be appreciated (Figure 1). These observations highlight the role of a genetics evaluation, as other specialties such as ophthalmology or GU specialists may not recognize some of these features due to the focus of their exams.

##### Future Considerations for Classification of WAGR Spectrum

In the present study, we reclassify the historical disorder “WAGR syndrome” to describe “WAGR spectrum” to broaden the characterization of phenotypic presentation and improve recognition of this complex rare genetic disorder. The genes involved in the 11p13 deletion and some of the phenotypes described in the WAGR population overlap with other disorders characterized by deletions or mutations of *PAX6* (aniridia syndrome/spectrum, OMIM #106210) and *WT1* (Denys-Drash syndrome/spectrum, OMIM# 194080; and Frasier syndrome/spectrum, OMIM# 136680); among others. More recently a disorder distinct from the other characterized 11p13 region spectrum disorders has been termed “chromosome 11p13 deletion syndrome” (OMIM# 616902). It is possible that these disorders could be considered separate entities within an umbrella term of “11p13 deletion/mutation spectrum disorder” (or similar terminology) due to shared clinical issues and/or phenotypes; however, the emerging evidence is too novel to establish this terminology, and this may create more confusion than clarity in the medical and research community. Determination of specific nomenclature, diagnostic criteria, and potential overlapping management strategies will require collaboration between various patient advocacy organizations, researchers, and physicians to determine whether these similar genetic abnormalities and/or phenotypic and clinical consequences may represent a wider spectrum that can be characterized in the future.

### Care Management Model for WAGR Spectrum

**Figure 5:**
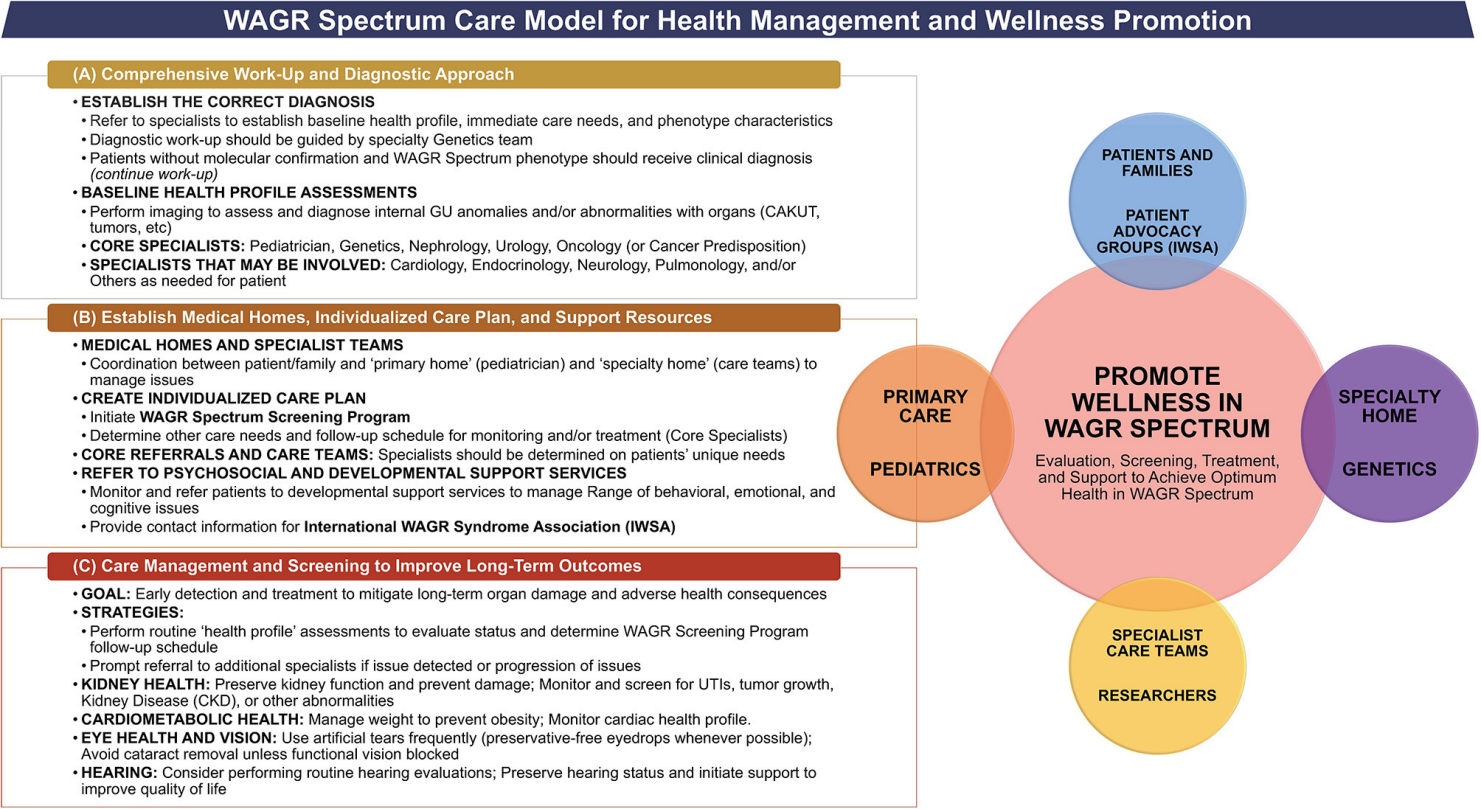
Care management model for WAGR spectrum. Model describes the primary goals, core specialists, and key considerations regarding **(A)** Initial Work-Up for suspected WAGR Spectrum diagnosis; and **(B)** Establishment of Care Team and Medical Homes once a diagnosis of WAGR Spectrum is made through molecular and/or clinical evaluation. A multi-disciplinary care management team is needed with additional specialists depending on individual clinical issues with focus on **(C)** Screening to Improve Long-Term Outcomes for patients with WAGR Spectrum. The goal of this model is to promote wellness within the entire WAGR community, which includes the involvement of care teams, patients, and their families. Psychosocial support should be emphasized.

An individualized care management approach is warranted in all patients with WAGR spectrum, as a variety of clinical issues can occur. A number of common issues observed in the population suggest that a team of core specialists is needed for a multi-disciplinary care team approach to patient care. We propose a care management model for WAGR spectrum (Figure 5), and discuss considerations that are common to all patients, as well as other considerations that may be unique to certain subgroups of patients within the spectrum. This model was developed utilizing the available previous evidence for WAGR syndrome available in correlation with the results derived from the WAGR Discovery Cohort. Information was synthesized and discussed between the research team and representatives from the International WAGR Syndrome Association (IWSA) to develop guidelines applicable for clinical practice and for patient-family wellness. A similar methodology to development of the WAGR Spectrum Care Model was previously utilized by some of the authors to develop a care management model for BWSp, or Beckwith-Wiedemann Spectrum (14, 15), which affects imprinting genes on chromosome 11p15 and represents a distinct heterogenous (epi)genetic disorder from the 11p13 deletion in WAGR, but shares some common characteristics with the WAGR population such as complex care management needs and tumor predisposition requiring cancer screening programs.

#### Initial Work-Up Considerations

Any patient presenting with aniridia should prompt referrals to a core team of specialists (Figure 5A), with additional specialists as warranted by individual clinical issues. All patients should receive a full abdominal ultrasound and a pelvic ultrasound in order to detect any anomalies; additional imaging or other studies may be required for individual issues as determined by the care team. It may be helpful to have at least some, or all evaluations performed prior to the genetics visit, as this information could aid in the diagnostic considerations. We recommend that geneticists and genetic counselors order genetic testing when possible, as they can determine the best molecular studies required for diagnostic confirmation. Patients suspected for WAGR spectrum should be considered at risk for WT development and receive renal ultrasounds every 3 months while the diagnostic work-up occurs.

#### Genetics Referral and Role

We recommend a genetics referral in patients presenting with aniridia or those suspected of having WAGR spectrum. A geneticist can determine the most appropriate molecular analyses to be performed, as a variety of molecular tests have been suggested for these patients and often a combination of multiple methods is needed for an accurate diagnosis.

For newborns with aniridia, first tier genetic testing includes Multiplex Ligation-dependent Probe Amplification (MLPA) and karyotyping to detect 11p13 rearrangements, however these methods do not always allow for a complete picture of the high complexity of chromosomal deletions and breakpoints that can occur (16). Previous recommended molecular testing for WAGR included a combination of high-resolution chromosome study and molecular cytogenetic fluorescence *in-situ* hybridization (1). More recently, the use of customized array-based comparative genomic hybridization (aCGH) with high density probe coverage of the 11p13 region to study chromosomal rearrangements in the WAGR locus has been reported as a useful method to refine molecular analysis and allow for individualized patient follow-up (16).

Once the diagnosis of WAGR spectrum is established, geneticists and genetic counselors can serve as the “medical home” for patients and help determine appropriate referrals as well as provide anticipatory guidance based on the patients” specific molecular diagnosis to support an individualized care management approach. Genetic counselors can also be considered part of the psychosocial and anticipatory care team approach. An increased focus on educating families about the specific diagnosis is recommended, as we observed a high frequency of participants in the WAGR Discovery Cohort who were unsure of their child's specific genetic results (Supplementary Table S1).

Routine follow-up with genetics is recommended, with specific frequency determined by individual patient issues. Annual follow-up can be considered for all patients, with more or less frequent visits depending on clinical history and family preferences.

#### Role of the Pediatrician or Primary Specialist

The pediatrician can assist in routine monitoring of development and growth and should serve as the initial point of contact for non-urgent medical issues. Pediatricians caring for patients with WAGR spectrum should be prepared to refer patients to specialists for management at the initial suspicion of potential clinical issues. Pediatricians can work with other specialist teams to help monitor and order health screening, and other clinical laboratory or imaging testing needed such as Wilms tumor screening. Increased attention to cardiometabolic health is suggested, with prompt referral to appropriate specialists if abnormalities arise. Transitioning to specialized care teams should also be a focus as patients age throughout childhood and into adulthood—the pediatrician, care teams, and family should work together identify the specialists that are involved throughout the patient's life.

#### Medical Home

The need to establish a medical home and involve families in the care of patients with WAGR spectrum has been previously highlighted (1) and we agree this is essential for care management. The medical home can be considered the combination of the pediatrician (or primary care specialist) and geneticist (or cancer predisposition specialist), depending on specific patient issues and family preferences. This team can help coordinate referrals to other members of the care team and participate in on-going monitoring and care management for each patient. The genetics team can help establish the core team of specialists required for the individualized management plan at the “specialized medical home” and additionally work to coordinate with the primary medical home for referrals and care coordination aspects. For patients with an established genetic diagnosis, the geneticist may involve other core specialists as available at the institution—such as cancer predisposition specialist teams. If available at the patient's “medical homes” we suggest involvement of social workers, nurse navigators, and/or complex care coordinators as possible to help alleviate the burden of care coordination from providers. These services will also provide a direct source for families to routinely engage with in addition to their medical providers and can help provide psychosocial resources for families.

#### Core Referrals and Multi-Disciplinary Care Team Model

Once a diagnosis of WAGR spectrum is established, referrals to a core multi-disciplinary team of specialists should occur to identify the clinical issues present and determine an individualized care management approach. Core specialists and considerations are summarized in Figure 5B and additional specialists may be warranted based on each patient's clinical issues (Table 9). Common considerations in all patients include management of aniridia and/or other eye issues, genital and nephro-urological anomalies (kidney screening); range of behavioral and neurodevelopmental delays, and potential cardiac, endocrine or pulmonary abnormalities.

**Table 9:** Additional health care considerations for patients with WAGR spectrum.

**Primary issue**	**Potential specialists**	**Considerations**
Feeding issues	• Feeding Team • Gastroenterology • Pulmonology	• Identify potential underlying issues to determine best management approach • Nutritionist can help if dietary modifications are needed
GU Abnormalities *(genital, kidney, and urinary tract)*	• Urology • Nephrology • Gynecology • Specialist in Disorders of Sexual Development	• Identify potential kidney or urinary tract anomalies • Screen for early detection and treatment of UTIs • Identify any internal genitourinary (GU) anomalies such as streak ovaries in females • Consider periodic evaluation as patients age
Sleep issues	• Pulmonology • Sleep Medicine Specialists • Consider sleep study	• Identify whether obstructive sleep apnea (OSA) is present and contributing to abnormal sleep • Sleep medicine specialists may provide further guidance
Hearing and frequent infections	• ENT • Allergist • Pulmonology • Hearing Specialist	• Respiratory tract infections, including pneumonia, are frequently reported • Ear, nose, and throat (ENT) procedures are common • Hearing preservation is critical to patients due to compromised vision • Consider performing routine hearing screens
Musculoskeletal issues	• Orthopedics • Neurology	• Hypertonia, hypotonia, and ataxia can occur • Scoliosis is a common issue • Use of orthotics may be necessary
Congenital cardiac anomalies	• Cardiologist	• Variety of cardiac defects can occur • Routine follow-up and/or interventions may be needed • Cardiac evaluation prior to surgical procedures is recommended

Ocular issues beyond aniridia were previously reported by Fischbach, Trout (1) and we observed a variety of common issues (Table 6). Recent evidence among those affected by PAX6 mutations have demonstrated genotype-phenotype between mutations and macular morphology (17). Further study into PAX6 deletions could yield specific genotype-phenotype within WAGR spectrum. Ophthalmologists should be prepared for the possibility of multiple ocular issues to affect patients and develop appropriate follow-up and screening. Preservation of vision is essential (Figure 5C).

#### Anticipatory Guidance and Psychosocial Support

Caring for patients affected by WAGR extends beyond childhood, as evidenced by almost all the Discovery Cohort adult population reported as dependent and living with parent/relative or at a skilled care facility. A variety of specialists and support services are needed for patients and their families; these should be initiated at the same time as suspected/confirmed diagnosis and modified for the patient and family's needs throughout their lives.

Collaborative teamwork is essential to create a supportive environment to facilitate individualized patient care and management approaches. A recent cross-sectional study among patients living with 81 different rare diseases found common unmet needs related to health system information and patient care, and patients additionally felt lack of adequate psychological support and education (18). Psychosocial support for caregivers is also critical as parents of children with a rare disease face a variety of challenges that have been described as a “jungle gym under construction” due to lack of education and support (19); parents of children with neurodevelopmental disorders have additionally reported feeling social isolation and exclusion (20). A rare disease diagnosis and the complex health care issues that present can also create the additional burden of navigating health insurance costs and approvals within the United States (21).

##### Developmental and Behavioral Support Services

Referral to services such as Early Intervention, developmental and behavioral specialists, and special education services will help support children as they age (Figure 5B). More than 70% of participants older than 18 years in the WAGR Discovery Cohort had completed some form of high school or additional education, and some participants reported part-time or full-time employment, which highlights the beneficial role these services can provide in achieving successful outcomes.

The results of this study and previous studies confirm that a wide range of cognitive and behavioral/emotional issues can affect patients. Consistent rates of ADD/ADHD have now been reported across three studies, and it is estimated that ~25% of patients with WAGR may be affected by attention issues (Tables 3, 4). A variety of potential mechanisms leading to the range of neurodevelopmental issues seen in patients with WAGR spectrum have been suggested, and the underlying association is likely multi-factorial. Candidate genes such as *BDNF, ELP4, PRRG4, and SLC1A2* have been identified in relation to autism, cognitive/developmental delays, and behavioral problems (5, 22–24). Other than underlying genetic mechanisms, several other factors in WAGR may predispose patients to the higher rates of neurodevelopmental and behavior issues observed. From a psychosocial perspective, the impact of chemotherapy treatment and repeated hospitalizations leading to behavioral issues in patients with WAGR was previously highlighted (12). Involvement of psychosocial support resources can help manage this burden.

Other supportive services such as physical therapy (PT), occupational therapy (OT) and speech therapy may benefit patients, as a high rate of these services were used by the WAGR Discovery Cohort participants. We observed a high rate of speech disorders in the present study, which has not been highlighted in previous WAGR cohorts. Deletions in the *ELP4* gene have been shown to lead to language impairment and may represent a candidate gene (24). The range of speech issues/disorders suggest that at least some patients within WAGR spectrum may face challenges with communication, and the pediatrician and parents can work to help evaluate the child's unique developmental progression based on clinical history (i.e., delay due to prematurity or clinical treatment versus consequences of WAGR deletion). Many participants reported use of at least one assistive device for support (Table 8), and it may be possible to develop assistive devices that can support communication for patients with visual impairment such as the WAGR population in the future to improve well-being.

##### Psychosocial Adjustment and Support

At time of diagnosis, referral to psychosocial services is encouraged for all patient families as the diagnosis of a rare disease not only affects patients, but their families as well. Genetic counselors can assist in long-term monitoring and counseling. Other providers such as psychologists, psychiatrists, social workers, etc. can also assist in supporting patients and families. Patient advocacy organizations such as the International WAGR Syndrome Association (IWSA) can play a key role in helping families adjust to the diagnosis, share their concerns and experiences, and obtain appropriate care. Families should be given contact information for organizations such as the IWSA at time of diagnosis, or soon after.

##### Contact Information for the International WAGR Syndrome Association

Website: https://wagr.org/

Email: reachingout@wagr.org.

### Key Considerations for Monitoring Health

The primary goals and key considerations for monitoring health in patients with WAGR spectrum are described in Figure 5C. All patients should receive an individualized care management approach. A focus on routine kidney surveillance and cardiometabolic profile should be emphasized to maintain optimum patient health and improve long-term outcomes.

#### Preservation of Hearing

We observed higher rates than previously reported of hearing issues within the WAGR Discovery Cohort (~15% affected). Consistent rates of tympanostomy tube placement have been reported between 2005 and the present study (Supplementary Appendix), and it is possible that history of frequent ear infections may be contributing to adverse hearing status. This clinical issue was not included in the Registry to assess the frequency; future studies should evaluate this issue to further explore potential associations.

As patients with WAGR spectrum are typically affected by compromised vision, preservation of hearing is crucial for quality of life. We suggest careful monitoring of hearing function in patients, with prompt treatment of ear infections and other illnesses that could affect hearing status.

#### Evaluation for Internal Genital, Kidney, and Urinary Tract Anomalies

It appears that participants are frequently affected by both GU and kidney abnormalities; however genital or urinary tract anomalies were observed to occur without kidney abnormalities and vice versa. As discussed above, it may be more appropriate to define the “G” in WAGR spectrum as “genital or nephro-urological anomalies” to properly characterize the disorder. This reclassification may aid in the initial diagnosis work-up, as GU anomalies are typically less frequent among females compared to male patients. This reclassification additionally highlights the need to include nephrology specialists in addition to urologists and potentially gynecologists within the core care team.

##### Internal Genital Anomalies

Patients may not present with any obvious external genital anomalies; however, our results suggest a number of patients are affected by internal genital anomalies and may benefit from referral to gynecology or specialist for disorders of sexual differentiation in addition to the core referral to urology.

**Figure 6:**
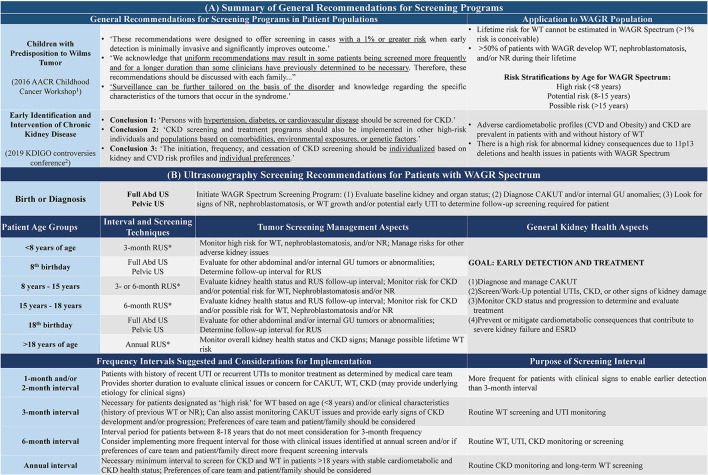
The WAGR spectrum screening program. All patients with WAGR Spectrum should receive routine renal ultrasonography (RUS) to monitor for kidney health throughout their lifetime. The principles underlying the recommendations developed for monitoring Wilms Tumor (WT) and Chronic Kidney Disease (CKD) with application to patients with WAGR are described in **(A)**; recommendation groups included the ^1^American Association for Childhood Cancer Research recommendations (3) and ^2^Kidney Disease: Improving Global Outcomes (KDIGO) Work Group (39). **(B)** Provides recommendations for the age groups and suggestions for specific interval periods that could be applied for the RUS screening program. These intervals are based on the patient's age and potential risk—below the age of 8 years, all patients should be considered at risk for WT development and receive appropriate monitoring through RUS every 3 months (*unless more frequent indicated by detection or suspicion for renal abnormality). After the age of 8 years, consideration for frequency of follow-up should be determined in combination with clinical history, current status, and discussion between care team and patient/family to determine the best individualized course. All patients should at least receive annual RUS to monitor for CKD risk. Additional imaging such as full abdominal U/S and pelvic U/S are warranted at certain periods as the patient ages to monitor the status of other internal organs and GU anomalies.

The proportion of female patients affected by internal GU anomalies may have been underappreciated in the past, as streak ovaries were reported by ~36% of female participants in our study, and close to 15% were affected by bicornuate uterus. The overall rate of internal GU anomalies reported by females in the WAGR Discovery cohort (34.1%) was double the rate of 17% previously reported in 2005 by Fischbach et al. (1); this may reflect wider recognition of these issues and highlights the need to evaluate for their presence in patients. As ovaries may be difficult to visualize in young patients at time of initial diagnostic work-up, it may be considered that additional pelvic imaging may periodically be performed to detect these anomalies that may initially be missed. We suggest performing this imaging in conjunction with kidney and other imaging as warranted by the patient's age and clinical history/status, as outlined in The WAGR Spectrum Screening Program (Figure 6).

##### Nephro-Urological Anomalies

Although the GU system includes the kidney, the presence of kidney abnormalities appears to be underrepresented in previous WAGR cohorts, with the focus primarily on genital and urinary tract abnormalities. In 1984, it was reported that renal malformations were rare in patients with WAGR, with four cases affected among 37 literature cases at the time (13). In more recent cohorts, a variety of kidney issues such as renal cysts, horseshoe kidney, hypoplastic kidney, and unilateral renal agenesis have been reported, however these issues were reported in single patients and were not common (1, 7). In the WAGR Discovery Cohort, close to 40% of participants reported a kidney issue that was consistent with a congenital anomaly of the kidney and/or urinary tract (CAKUT), which suggests an association within WAGR spectrum. A table summary of common issues affecting the kidneys, genitals, and/or urinary tract reported across WAGR populations is available in the Supplementary Appendix.

##### Potential mechanisms leading to CAKUT in WAGR spectrum

A potential mechanism leading to the association between WAGR and CAKUT may include interactions between the PAX2 gene and genes in the WAGR region (*WT1* and/or *PAX6*). It has previously been reported that PAX2 is one of the most common mutations found in patients affected by CAKUT (25). *PAX2* plays a key role in kidney development, as it activates downstream targets that ultimately lead to podocyte formation and it has been hypothesized that dysregulation of *PAX2* targets, such as *WT1*, can disrupt the development and/or function of the podocyte which in turn leads to FSGS (26). Evidence suggests *PAX2* strongly represses the expression of *WT1* and conversely, that *WT1* is a *PAX2* repressor (26). The role of *PAX6* mutations on altered *PAX2* function and phenotypic manifestations of optic-nerve malformations has also been suggested (27). Further evidence is needed to establish the underlying mechanisms leading to the observed association between CAKUT and WAGR spectrum.

### Managing Health Risks (Routine Surveillance)

Patients with WAGR spectrum have an increased risk for developing WT, kidney disease, and obesity and should receive routine monitoring to manage these risks. Guidelines published by the World Health Organization in 1968 advocated for population-based screening programs that optimize early detection that allows for less severe disease stages and more effective treatments (28). While some of the clinical issues that affect patients with WAGR cannot be avoided, the awareness of the potential for abnormal kidney and cardiometabolic profiles and routine surveillance can help provide appropriate management specific to the WAGR population.

In these sections, we provide an overview of common issues within the WAGR spectrum population with considerations for care management and surveillance strategies. Additional evidence is needed in order to evaluate additional surveillance strategies that may provide a benefit to patients with WAGR spectrum to guide care recommendations, however extended renal ultrasound (RUS) surveillance seems as an appropriate strategy to manage risk in the interim. We discuss considerations for development of a screening program specific to patients with WAGR spectrum in the sections below.

#### Wilms Tumor Surveillance in WAGR Spectrum

All patients with predisposition for Wilms Tumor (WT) development should be referred to cancer predisposition or oncology clinic as these specialists can provide up-to-date information and work with the patient's general pediatrician to manage surveillance. Current recommendations for WT screening in all predisposition syndromes include renal ultrasounds every 3 months from birth or diagnosis until 7 years of age (3). These recommendations were provided as general uniform guidelines by the American Association for Cancer Research (AACR), although it was commented that in the future, guidelines should be tailored to specific syndromes and genetic etiologies (3). Specific to WAGR spectrum, the recommended length of WT screening has ranged from beginning at time of birth or diagnosis until 5 years to 8 years of age (7). In the below sections, we review current available evidence regarding WT in the WAGR population and provide suggestions related to potential modifications for surveillance recommendations.

##### Age at Presentation of Wilms Tumor

Patients with WAGR spectrum tend to experience an earlier age of initial WT development compared to patients with non-syndromic WT (4). We observed the median age at initial development of WT or isolated nephrogenic rests (NR) reported in our cohort was 19 months with an interquartile range of 11–28 months, which is consistent with the median ages previously reported in WAGR cohorts (1, 4, 7). In the present study, all patients with reported WT developed their initial tumor by 8 years of age and 95% of patients developed the initial WT by 5 years of age (Table 2). Recently, Hol et al. suggested WT surveillance until age 5 years in WAGR, as 100% of patients in their study experienced the initial WT diagnosis by this age (7). These frequencies differ compared to earlier WAGR cohorts: Fischbach et al. described four of 19 patients with WT (21.1%) were diagnosed after the age of 5 years and Breslow et al. previously reported 10 of 64 patients (16%) in their cohort with development after the age of 4 years (1, 4).

Multiple patients have been described with development of WT past the age of 7–8 years and/or relapse occurring years after initial diagnosis. The oldest reported age of WT development in WAGR appears to be 25 years (1). Breslow et al. reported one patient who was diagnosed with *de novo* WT in the contralateral kidney that occurred 12.1 years after the initial diagnosis (4). Hol et al. reported a patient with a stage 1 unilateral tumor diagnosed at 21 months that later developed a contralateral tumor at age of 9 years (7 years after original diagnosis), and another patient with bilateral nephroblastomatosis which progressed to WT first detected 13 months after initial diagnosis, and then again 4.6 years after the initial diagnosis (7).

Breslow et al. previously reported that two patients had been incorrectly labeled as “relapse” when in fact the WT was *de novo* disease in the contralateral kidney (4). In the present study, three participants reported “relapse” although we are unsure whether these represented true relapses or second primary tumors due to the structure of question and data provided. In two participants, the “relapse” occurred 11 months and 25 months after the original diagnosis. Late presentation occurred in one participant at 19 years, 7 months of age which was more than 17 years after the first WT diagnosis and represented the third occurrence; favorable histology was reported for all instances. In this participant, given the length of time between tumor detection, we believe this represents a new occurrence of WT; alternatively, this patient could represent an additional case of extremely late relapse in patients with WAGR. Specific classifications of “relapse” versus *de novo* in this population will require more thorough pathological and molecular studies, however these observations demonstrate that WT development beyond the age of 7-8 years has occurred in multiple patients with WAGR spectrum, and long-term risk may have been underrepresented in previous WAGR syndrome cohorts.

##### Wilms Tumor Risk Classification and Age Stratification in WAGR Spectrum

The risk for WT until at least the age of 8 years was confirmed in the present study. While it appears that patients with WAGR spectrum tend to develop tumors earlier than “non-syndromic” patients, it also appears that WAGR may not represent a “childhood cancer predisposition disorder”—we observed one participant with third WT diagnosis at 19 years of age with history of first WT diagnosed in early childhood. To recognize the lifelong potential for WT development within this population, we designate the following WT risk classifications with stratification by age group:

- Birth−8 years: High risk (~*50% prevalence established)*
- 8 years−15 years: Potential risk *(multiple patients reported)*
- >15 years: Possible risk *(up to 25 years of age reported)*

Risk for development of precursors, including nephrogenic rest and nephroblastomatosis, should be considered in addition to risk for WT, and early detection of these precursors may aide in facilitating intervention to prevent full WT progression, as previously reported (7).

##### Effectiveness of Wilms Tumor Screening

The benefit of WT surveillance in the WAGR population was recently highlighted by Hol et al., who reported a significant decrease in tumor volume in patients diagnosed through screening compared to symptomatic individuals which enabled a high rate of nephron-sparing surgery (7). The reduced specimen weight in patients with WAGR diagnosed through screening was also previously reported by Breslow, Norris (4). Additionally, WAGR patients diagnosed through screening have been shown to have a more favorable stage distribution compared to non-syndromic WT patients and compared to WAGR patients that had not been screened (4). Similar results have been reported for other WT predisposition populations (29), further supporting the role of cancer screening in at-risk populations.

The primary purpose of screening is to enable earlier detection and reduce treatment burden to improve outcomes, which is essential within the WAGR population due to the association of chronic kidney disease (CKD) and end-stage renal disease (ESRD) in patients. In the study by Hol et al., the decreased tumor volume enabled a high rate of nephron-sparing surgery (7), which is recommended for all individuals with cancer predisposition (30). To facilitate nephron-sparing surgery, preoperative chemotherapy is suggested (30) and has been reported to decrease tumor size in 50% of patients with WAGR (7).

##### Other Kidney Growths and Abnormalities

In addition to WT and precursor development risk, it also appears that some patients may develop non-malignant growths in their kidney(s). Renal cysts were reported at a rate of 5.6% by the WAGR Discovery Cohort, which have been reported previously (1, 7). We also observed five participants with kidney stones (7.0% of cohort), which were not described previously (Supplementary Appendix). These observations highlight the range of growths that can occur in the kidneys of patients with WAGR Spectrum and the need to perform comprehensive work-up to identify the correct type of kidney abnormality to prevent unnecessary surgery or treatment.

##### Considerations for Duration of Wilms Tumor Screening in WAGR Spectrum

Due to the rarity of WAGR spectrum and lack of consistent outcome data regarding length of risk, monitoring past the age of 7–8 years can be considered. The potential for lifelong WT surveillance was previously suggested (1). More recently, it has been suggested that extended surveillance in WAGR patients with a previous diagnosis of WT or nephroblastomatosis is warranted, as data suggest NR carry a long-lasting risk of WT progression in patients with WAGR (7).

Although the evidence provided by Hol et al. supports the role of tumor screening within the WAGR population, this study also reported an alarming rate (30.8%) of patients with WAGR and WT or nephroblastomatosis who presented with a palpable mass or symptoms (hematuria) rather than diagnosed through screening (14). In two patients, the diagnosis of WAGR was not suspected until after the WT diagnosis (14); highlighting the need to identify patients at risk to initiate tumor screening programs early. There were also three patients with a diagnosis of WAGR established prior to the tumor detection (14); it is unknown whether these patients were not receiving the standard screening protocol suggested, or it is possible that these patients were beyond the age of 8 years.

The guidelines for WT screening programs are to detect tumors in 90–95% of the population at risk, with routine surveillance performed within the age range of this risk (29, 31). Within WAGR Spectrum, it is not currently possible to determine the age or type of first WT manifestation that may develop within the population; furthermore, the risk for additional WT development in the context of history of NR, etc. cannot be estimated. Therefore, given the high frequency of WT overall, we recommend WT screening beyond age of 8 years for patients with a diagnosis of WAGR Spectrum.

At the age of 8 years, a discussion between the patient's family and multidisciplinary care team including at least the geneticist, oncologist, and nephrologist can help determine the appropriate follow-up schedule for WT monitoring. Specialists should consider the patient's previous medical history, as well as current issues that could help inform an appropriate screening interval. Recommendations for screening intervals for patients older than 8 years of age are discussed below in The WAGR Spectrum Screening Program (*section The WAGR Spectrum Screening Program*). As more evidence specific to the WAGR population emerges, specific WT screening recommendations are likely to change; however extended renal ultrasound surveillance will also help monitor the kidneys for other non-malignant issues and detect any signs of kidney damage.

#### Chronic Kidney Disease in WAGR Spectrum

Development of kidney failure in WAGR spectrum is a primary concern for long-term outcomes and health management and determining the underlying reasons for this association is crucial to guide care management approaches. In the present study, we observed a quarter of participants with some degree of reported kidney failure, and additional participants affected by FSGS and/or proteinuria without kidney failure. Fischbach et al. first reported a higher incidence of glomerular disease in WAGR syndrome, with FSGS the most common type found (1) and the results of the WAGR Discovery Cohort support this association. We observed close to 20% of participants affected by FSGS, with three participants reportedly affected by FSGS in the absence of kidney failure. Some participants reported isolated proteinuria, suggesting potential early signs of chronic kidney disease and renal failure.

Historical progression of WAGR syndrome/spectrum suggests that chronic kidney disease (CKD) has been prevalent within this population from early reports but was not widely recognized until the early 2000s when patients presented with severe kidney failure and unfavorable long-term outcomes (Supplementary Appendix). More recent studies provide evidence that the currently living WAGR population has been diagnosed with less severe forms of kidney disease (Supplementary Appendix), which provides an opportunity to prevent the adverse outcomes that occurred in previous generations of individuals living with WAGR. In the below sections, we summarize the available evidence that supports the formal association between WAGR Spectrum and predisposition for development of chronic kidney disease (CKD).

##### Prevalence of Kidney Failure in WAGR Population

In patients affected by WAGR and WT, it was initially estimated that the risk of chronic renal failure ranges from 38 to 53% in patients at 20 years from diagnosis of WT, and that risk is high compared to WT survivors without WAGR (4). A follow-up study confirmed that patients affected by WAGR and other WT1 malformation syndromes have the highest risk for ESRD, and revised the 20-year risk estimates to 36% incidence for unilateral WT and 90% incidence for bilateral WT in patients affected by WAGR (32). The study additionally commented that ESRD in WAGR tended to occur relatively late, often during or after adolescence (32), which highlights the need for long-term renal health screening in this population. A more recent report found that 25% of patients affected by WAGR and WT were affected by kidney disease, with time to onset between 2 and 13 years after WT diagnosis; it was commented that the lower risk observed in this study may have been a result of lack of extended long-term outcomes and that risk may be higher (7). In the present study, we observed that 36% of patients with a history of WT reported some degree of kidney failure; age from WT diagnosis was not available. As we were unable to evaluate the age of patients and time from WT diagnosis, it is likely that longitudinal follow-up of the Discovery Cohort would yield a higher proportion, as evidence suggests that the observed prevalence in our population is on the lower end of the previous incidence rates reported.

One previous study evaluated the overall proportion of kidney disease in the overall WAGR population, reporting 60% of patients with WAGR over the age of 12 years had evidence of renal failure and two patients did not have a history of WT (1). This suggests the causative factor is likely multifactorial. We observed an overall rate between 20 and 33% of participants reportedly affected by at least one CKD feature. As commented above, the age of our participants was unknown and based on the evidence from Fischbach et al., it is possible that the true rate within WAGR spectrum is underestimated through the Discovery Cohort. We additionally observed multiple participants with CKD features without WT history, which provide observations to support other potential mechanisms leading to CKD risk in this population.

##### Potential Mechanisms Contributing to Risk

The role of *WT1* abnormalities in kidney and renal failure development has previously been described (1, 33, 34). Limited information on kidney pathology in patients with WAGR are available, however it appears some differences in the kidney are present that may predispose patients to developing FSGS and renal failure. It was previously found that non-tumor kidney biopsies from seven patients with WAGR had significantly smaller glomeruli sizes compared to control samples, similar to the small glomeruli seen in Denys-Drash syndrome (OMIM#194080), which supports a molecular predisposition and the role of *WT1* (33). Patients with WAGR and WT have an additional risk for CKD development, as therapies such as nephrectomy, chemotherapy, and radiation can cause decreased renal mass leading to proteinuria, FSGS, and kidney failure (1, 33). Fischbach et al. previously suggested that multifactorial mechanisms exist, as some WAGR patients without WT develop CKD and not all patients with WT will develop CKD (1).

Evidence for potential non-WT related causes of renal failure in patients with WAGR are available through case reports and series. Iijima et al. described three patients affected by FSGS and two did not have a history of WT (34). In the patient affected by FSGS with a WT history, histologic examination of the kidney after chemotherapy but before radiotherapy treatment had shown a few glomeruli with segmental sclerosis without evidence of tubulointerstitial lesions (associated with chemotherapy treatment), suggesting that the WT treatment was not the primary cause of the FSGS (34). Previously, Merta et al. described a patient diagnosed with FSGS that was thought to be secondary to clinical factors (obesity with arterial hypertension), as the kidney histology was not consistent with those expected from WT treatment effects (35). Adverse kidney health has also been reported to occur before the development of WT, as a patient was described with hypertension and nephrotic syndrome diagnosed by 7 months of age, which was 11 months prior to WT diagnosis (33), and suggests an underlying predisposition rather than consequence of WT or treatment. Le Caignec et al. reported a patient with some areas of renal subcapsular focal and segmental glomerulosclerosis (FSGS) observed on autopsy at 14 months of age, and the patient did not have a history of WT (36). A recent case report described a 37-year-old man affected by classic WAGR syndrome and obesity who experienced gradual deterioration of renal function over a 2-year period despite never having a WT (37). Renal biopsy was consistent with FSGS and urinalysis showed proteinuria without microscopic hematuria and no evidence of WT (37). This patient highlights the notion that WT history is not the exclusive cause for adverse renal health in patients with WAGR spectrum, and that a lifelong risk of renal failure likely exists in at least some patients.

##### Cardiometabolic Issues Contributing to Chronic Kidney Disease

Obesity and metabolic abnormalities have been suggested as a primary factor leading to increased renal failure risk in WAGR syndrome (33), and our observations support these factors as potential mechanisms leading to the association of increased CKD risk in WAGR spectrum. We evaluated certain clinical characteristics of the 28 participants affected by a CKD feature: proteinuria, kidney failure, and/or FSGS and found high rates of cardiovascular disease and obesity (Figure 3). This led to the hypothesis that participants may be affected by metabolic syndrome, and we applied common criteria to assess the potential rate in the WAGR Discovery Cohort. We found that more than 25% of participants affected by CKD were possibly affected by metabolic syndrome. Furthermore, positive metabolic syndrome criteria were demonstrated in 60% of participants affected by CKD without WT history, suggesting the combination of *WT1* abnormalities and metabolic issues are likely the main contributing factors to the CKD development in these patients. We additionally observed higher kidney failure stages in patients affected by WT and multiple cardiometabolic factors, which suggests that degree and risk of renal failure development may occur in a gradient as a result of the cumulative effects of the clinical issues present in each patient. These observations highlight the multifactorial mechanisms and need to screen WAGR patients for these issues to prevent or delay future chronic kidney disease.

##### WAGR Spectrum Is a Population at Risk for Chronic Kidney Disease

Our results suggest that chronic kidney disease (CKD) is associated with WAGR spectrum and patients should be considered at risk for its development. Chronic kidney disease (CKD) is defined as “*abnormalities of kidney structure or function, present for* >*3 months, with implications for health”* (38). The Kidney Disease: Improving Global Outcomes (KDIGO) conference consensus recently suggested that CKD screening and treatment programs should be implemented in “*high-risk individuals and populations based on comorbidities, environmental exposures, or genetic risk factors”* (39). A variety of risk factors have been associated with CKD, and we have summarized the rates of these features observed within the WAGR Discovery Cohort in Supplementary Table S4.

All patients with WAGR Spectrum could be considered to have a predisposition for CKD development at birth due to the 11p13 deletion and affected genes such as *WT1*; and some patients may have additional risk identified early through history of low birth weight and/or detection of abnormal kidney(s) at birth with a diagnosis of CAKUT. As patients age, additional common clinical issues such as recurrent urinary tract infections, history of nephrogenic rest or full WT development, and adverse cardiometabolic profiles can create additional harm to the kidneys, contributing to the progression of CKD within this population.

##### Emerging Information on Chronic Kidney Disease in WAGR Spectrum

Although statistical exploration was outside the scope of the present study, we recently presented evidence at the 53rd Congress of the International Society of Pediatric Oncology (SIOP 2021) evaluating risk for CKD within the WAGR Discovery Cohort population (40). We found that CKD is prevalent in patients with and without history of WT development and cardiometabolic features such as hypertension and obesity appear to provide a higher risk by univariate analysis than history of WT development; highlighting the multifactorial nature contributing renal disease progression and need to perform routine CKD monitoring in patients with WAGR (40). Tracy et al. also presented evidence at SIOP 2021 from the results of a statistical analysis evaluating renal failure rates between patients with and without history of WT and WAGR using data collected through the IWSA WAGR Syndrome Patient Registry; finding that renal failure was significantly associated with WT history (53.1% renal failure rate in WAGR-WT) compared to children without WT (6.2% rate), with five patients experiencing renal failure without WT history (41). Chemotherapy was significant by univariate analysis; however, multivariate analysis demonstrated nephrectomy as the only significant factor associated with renal failure, highlighting the need to consider nephron-sparing surgery (NSS) when possible (41).

These results suggest that WT development may contribute to CKD risk, and nephrectomy appears to provide an increased risk for severe CKD manifestations such as renal failure. In patients without history of WT and/or nephrectomy, it is likely the burden of other clinical factors present contribute to the progressive development of CKD disease forms. All patients should be routinely monitored for these clinical issues, with specific follow-up and screening determined by the individual's unique clinical history and current clinical status.

#### Kidney Health Management Strategies for Patients With WAGR Spectrum

To manage CKD risk, we suggest a multi-disciplinary care team approach that consists of at least nephrology and endocrinology to monitor cardiometabolic and renal health. Cardiology may be involved and can be determined by the patient's unique needs. Routine monitoring of kidney function and cardiometabolic health is warranted, and specific monitoring schedules can be determined by the care team. As discussed in the WT section above, one could consider performing routine renal ultrasound (RUS) screening to detect non-tumor kidney abnormalities that may be present. Evidence suggests that kidney failure typically presents during or after adolescence in patients with WAGR (1) which raises the notion of extended renal ultrasound surveillance in the WAGR spectrum population. Obesity and hypertension management may also help reduce kidney disease risk. In patients with isolated proteinuria, prompt treatment may help reduce progression to more advanced CKD degree states as previously suggested (1). We provide suggestions and recommendations to monitor kidney health status for patients with WAGR phenotypes and/or 11p13 deletions consistent with a diagnosis of WAGR Spectrum.

##### Monitoring for Urinary Tract Infections

Patients with WAGR should be considered at high risk for development of urinary tract infection (UTI) and may be affected by multiple or recurrent infections throughout their lifetime. It does not appear this clinical issue has been described in association with WAGR in previous cohort studies. Approximately 20% of participants in the Discovery Cohort reported ‘recurrent UTIs’ within the kidney/renal category, which provides a concerning observation in relation to preserving kidney health for this population as the risk for renal damage after childhood UTI has been estimated at ~15% (42). These rates are much higher than reported among the general pediatric population: prevalence among infants and young children up to 2 years of age in males is estimated at 1.9–3.3% and in females at 6.5–8.1%; during adolescence, females are more commonly affected and the prevalence of UTIs in adolescent males has been reported as “very low” (42). While we did not have data to provide age-stratification within the present study, the rates observed suggest patients with WAGR spectrum likely experience a higher risk for UTIs compared to the general population.

The increased UTI risk presents a challenge to preserving kidney health, as there is risk for permanent kidney damage that can lead to long term consequences such as hypertension and impaired renal function (42). The frequent UTIs in the WAGR population are likely a consequence of the presence of CAKUT and/or GU anomalies the patient is born with. This provides evidence that patients with WAGR spectrum should be considered at high risk for UTI development and receive appropriate monitoring to manage this apparent predisposition. Several approaches to performing imaging studies have been suggested to monitor for UTIs and include the “bottom-up” and “top-down” approaches, with common applications between both methods including the use of RUS as imaging modality during the first line approach or work-up (42). Additional considerations for UTI work-up and management can be determined by the patient's pediatrician/primary care physician and/or specialists such as urologists or nephrologists.

##### Challenges in Common Diagnostic Approaches to Potential Kidney Issues

Infants and young children have been shown to present with non-specific signs and symptoms of UTI, while after ~5 years of age the “classic urinary tract symptoms” are usually present (42). Furthermore, it has been commented that older children are “better able to verbalize symptoms and, for this reason, specific symptoms of UTI are more commonly identified” (42). In the context of patients with WAGR, the “R” phenotype can range from severe cognitive delays or intellectual disability to no cognitive/developmental issues reported. We additionally identified a variety of speech issues reported in the population, which presents evidence that basic expectations for children or adults to understand and/or communicate symptoms cannot be applied to patients with WAGR spectrum. As the goal of screening programs is early detection, routine RUS could serve to monitor for potential UTI before the “classic” clinical signs appear, prompting earlier intervention to preserve kidney function as suggested for pediatric patients with UTI risk (42). An additional challenge for UTI diagnostic work-up includes that clinical signs can be non-specific and overlap with other kidney abnormalities *(i.e., flank pain with hematuria could suggest UTI or potential WT development)*, the routine performance of RUS could help provide evidence to correlate with other clinical values such as urine and serum screening for CKD, or additional imaging studies if WT and/or NR is suspected.

##### Monitoring for Signs of Chronic Kidney Disease

A variety of structural or other imaging abnormalities detectable by RUS that suggest potential CKD risk or etiology have been established (38, 43), and we have provided a summary in Supplementary Table S4 with correlation to the rates observed in the WAGR Discovery Cohort to justify an association between CKD risk and WAGR spectrum. In addition to RUS screening, a variety of other clinical and biochemical markers have been established to monitor CKD and kidney failure risk, development, and staging criteria in addition to new CKD classification system criteria (38, 39, 43). Specific recommendations for these aspects for WAGR management are outside of the scope of the present study, however the proposed multi-disciplinary care approach model would enable tracking of cardiometabolic and kidney health profile status through the pediatrician or primary care provider in combination with a team of specialists (such as endocrine/weight management, cardiovascular, nephrology); the geneticist could serve to help to establish the specialists required for each individual patient based on their unique genetic and phenotypic presentation.

##### Considerations for Implementation of Multi-Disciplinary Approach to Kidney Health

There are a variety of underlying congenital anomalies and clinical issues that commonly affect patients with WAGR that likely contribute to the multifactorial progression of unfavorable kidney health and outcomes previously associated with the disorder (Figure 3, Supplementary Table S4). While some factors such as the underlying 11p13 deletion and CAKUT and/or GU anomalies that develop as a result cannot be mediated, careful monitoring and early detection of patients with signs of kidney disease progression can help achieve early treatment and intervention approaches to slow the progression of kidney damage. All patients with WAGR should receive routine biometric screening at all visits from their pediatrician and/or primary care provider to monitor biophysical profile, with additional urine and/or serum screening as indicated by the individual care plan developed for the patient. This “primary medical home representative” should coordinate with the patient's specialty care team that may consist of cardiologists, endocrinologists/weight management, nephrologists, urologists, and/or others as required to determine the frequency and specific screening program based on clinical status, history, and age.

#### The WAGR Spectrum Screening Program

We propose the initial recommendations that will comprise “The WAGR Spectrum Screening Program.” The goal of this program is to promote optimum health within the WAGR spectrum population to improve overall health status and long-term patient outcomes. We anticipate the specific recommendations that comprise this program will likely evolve over time as the WAGR spectrum population is better characterized, however we provide the initial framework and propose revisions to the duration of renal ultrasound (RUS) screening for all patients with a diagnosis of WAGR spectrum disorder. This program has been endorsed by representatives of the International WAGR Syndrome Association (IWSA).

##### Renal Ultrasonography Screening Recommendations for WAGR Spectrum

Lifelong kidney surveillance is warranted in the WAGR Spectrum patient population. The composite observations collected from the WAGR Discovery cohort and previous literature evidence suggest that patients with WAGR can be considered to have congenital kidney abnormalities and/or other adverse health profiles that can establish risk for kidney damage warranting routine lifelong imaging to assess kidney health. As discussed above, RUS has been shown to provide benefits for diagnostic work-up of common issues in the WAGR population that include CAKUT, CKD, UTI, and/or WT; aspects related to screening programs designed for these issues were taken into consideration when developing the suggested intervals for RUS screening in the WAGR population.

The following intervals to perform RUS could be considered until additional evidence is available:

- **3-month interval:** required until age 8 years for WT screening *(minimum frequency)*
  ◦ Consider more frequent interval if the patient has CAKUT, UTIs, or WT recently diagnosed *(or at clinical suspicion for)*.
- **6-month interval:** consider between ages 8 and 18 years *(if no recent UTIs, stable kidney appearance, no WT history, etc.)*
  ◦ Consider moving to 3-month intervals to monitor clinical status if abnormalities present or are detected by imaging.
- **Annual:** required in patients >18 years for CKD screening *(minimum frequency for RUS in patients with stable clinical status, childhood screening, and cardiometabolic profiles)*.
  ◦ Consider moving to 3- or 6-month intervals to monitor clinical status if abnormalities present or are detected by imaging.

Care teams are encouraged to consider the aspects of this program in the context of each individual patient with WAGR spectrum to determine the best care approach. The preference of the patient and/or family should also be taken into consideration when developing the screening interval for each individual with WAGR, as recommended by KDIGO for CKD screening programs (39) and the AACR for WT screening programs (3); performing 3-month screening intervals for WT risk in patients age 8 or older could be initiated to help provide reassurance of negative tumor growth at the family preferences.

#### Additional Ultrasonography Imaging Recommendations for WAGR Spectrum

Patients experience risk for other organ and internal GU anomalies that may not be initially detected during infancy or early childhood. At time of the scheduled RUS follow-up, additional ultrasonography (U/S) imaging of the abdomen or pelvis could be considered to enhance the surveillance process for clinical issues beyond the kidneys. Full abdominal U/S (rather than targeted RUS) could be performed in patients with clinical issues that warrant internal imaging to aide in the diagnostic work-up or to monitor the effectiveness of current treatment approaches. A pelvic U/S could also be performed to assess for internal GU anomalies that may present as patients enter puberty and adulthood.

We recommend performing these additional ultrasonography studies in conjunction with the RUS to allow for “same-time” data collection on the internal status of patients and to reduce the burden of medical visits and imaging on patients/families.

##### Additional Considerations for the WAGR Spectrum Screening Program

Adverse cardiometabolic profiles appear associated with more severe CKD and kidney failure within the WAGR population and have been established with high risk for CKD within the general population. The KDIGO conference consensus also concluded that “*The initiation, frequency, and cessation of CKD screening should be individualized based on kidney and cardiovascular risk profiles and individual preferences”* (39).

To monitor cardiometabolic health status, all patients with WAGR should receive routine biometric screening at all visits from their pediatrician and/or primary care provider to monitor biophysical profile, with additional urine and/or serum screening as indicated by the individual care plan developed for the patient. Any abnormalities, or rapid changes in growth curve tracking should prompt referral to specialists as indicated. The “primary medical home representative” should coordinate with the patient's specialty care team that may consist of cardiologists, endocrinologists/weight management, nephrologists, urologists, etc. to determine the frequency and specific screening program based on clinical status, history, and age.

##### Perceived Benefits of the WAGR Spectrum Screening Program

We anticipate that the **WAGR Spectrum Screening Program** will lead to earlier detection of UTIs and help assist in monitoring CKD development and treatment response. Further intervention studies can help clarify the appropriate screening tools specific to the WAGR population, however specific kidney surveillance beyond WT screening was previously proposed by (1). As discussed above, it is likely that the previous recommendations established for WAGR syndrome may not apply to WAGR spectrum, and further evidence will be needed to clarify guidelines specific to serum and urine kidney screening.

An additional benefit of this program includes continued surveillance for potential WT, nephroblastomatosis, or NR development beyond the age characterized with established risk. As proposed in *section Wilms Tumor Risk Classification and Age Stratification in WAGR Spectrum (above)*, we suggest that there is a potential for WT development after age 8 years, with the possibility of lifetime WT risk for patients with WAGR spectrum. This stratification may lead to parental worry; however, it was designed to increase awareness of the necessity for routine surveillance in this population beyond the first 8 years of life. We anticipate the increased duration of surveillance will likely contribute a psychosocial benefit to parents in the WAGR community, as parents in other cancer predisposition populations have reported decreased worry and overall benefit provided by tumor screening programs (44). Additional survey research within the WAGR community could help shed light on perspectives specific to this population.

This program will likely contribute a cost-effective strategy for health prevention in the WAGR population in addition to early detection for kidney preservation and the psychosocial benefits. Health care spending for patients with CKD represent at least 20% of all Medicare costs (43). Additionally, the severity of CKD exponentially increases Medicare expenses, with costs reported at $1,700 per-patient for stage 2 and $12,700 for stage 4 between 2013 and 2017 and dramatically increasing if ESRD develops (43). Other considerations for cost-effectiveness have been discussed by KDIGO, with suggestion to consider the $/quality-adjusted life year threshold that screening programs may provide with the consensus concluding that (#12): “*CKD screening in high-risk groups is likely to be cost-effective”* (39). Although not cost-effective for general populations, the use of RUS in conjunction with other biochemical markers has been shown to help monitor and detect CKD in both the pediatric and adult populations, and RUS can serve as a parameter for renal function (45, 46). As the prevalence of WAGR in the population is estimated at less than one in 500,000 births, it is likely the considerations for large populations do not apply to this unique patient group, and we suggest the low-cost and routine use of RUS represents a feasible approach for kidney health screening for patients with WAGR spectrum. Future studies could help validate the use of RUS and other screening tools for patients with WAGR through longitudinal follow-up of the WAGR Spectrum Screening Program.

#### Obesity Association in WAGR Spectrum

A high rate of obesity was observed among our cohort, with slightly more than half of participants affected. While some previous WAGR cohorts have reported prevalence between 13.3% (7) and 18.5% (1), a specific study evaluating obesity in WAGR found that 16 of 24 patients (66.7%) had developed obesity by the age of 10 years (2) which is comparable to the rate reported in this study. The first association between obesity and WAGR was suggested in 1994 by a case report describing severe obesity in a female affected by WAGR (10). An additional male patient with WAGR and obesity was reported in 2000, and the authors confirmed that obesity should be added to the WAGR spectrum, suggesting that a putative gene for obesity could be located within the 11p13 band (47). The third patient (male) affected by WAGR and severe obesity was reported in 2001 (11) and suggested the acronym WAGRO for the association of WAGR plus obesity. The first patient affected by hyperphagia, binge eating, and lack of satiety associated with WAGR and obesity was reported by Amor (48). This male patient had a history of rapid weight gain after birth and development of morbid obesity after the age of 17 years, with a BMI of 45 kgm^−2^ at 33 years of age (48). At this time, it was commented that further evidence was needed to establish an association between WAGR and obesity or if this observation was related to the increased risk of obesity with intellectual disabilities (48).

Gul et al. hypothesized that a gene in the 11p12p14 region was responsible for obesity due to the molecular findings in their patient and the first patient reported (10, 11). The role of *BDNF* gene in relation to WAGR and obesity was supported in a study evaluating 33 patients affected by WAGR syndrome (2). In this study, patients affected by *BDNF* deletions had significantly higher BMI scores throughout childhood and was evident by 2 years of age compared to patients without *BDNF* affected (2). This study also suggests that patients affected by *BDNF* deletions have an increased risk for obesity, as 100% were obese by age 10 years compared to 20% without the *BDNF* deletion (2), although it also implies that a *BDNF* deletion alone does not explain the obesity association.

Later onset of obesity has also been reported in some patients, suggesting that long-term monitoring of weight status is necessary and lack of early onset does not preclude risk of future development. Bremond-Gignac et al. reported a patient affected by large 11p deletion consistent with both WAGR and Potocki-Shaffer syndromes in which the onset of obesity did not present until after puberty around 17 years of age; hyperphagia was additionally reported (49). As described above, a male patient with morbid obesity developing around 17 years has also been reported (48).

##### Prevention and Management of Obesity

A staged approach to weight management for children and adolescents for the treatment of obesity has been proposed by Spear, Barlow (50). Initial prevention includes routine monitoring of BMI and healthy diet encouragement by the primary care office with referral to a dietitian for structured weight management if BMI increases (50). The later stages of this model include referral to a comprehensive multidisciplinary obesity care team comprised of a behavioral counselor, registered dietitian/nutrition, and exercise specialist with increased intensity of behavioral change strategies and greater frequency of patient-provider contact compared to earlier intervention stages (50).

Pediatricians can assist in standard weight monitoring in this population and should be aware of the likelihood of increasing BMI percentiles in order to promptly refer patients to specialty services if issues arise. Spear, Barlow (50) additionally recommended that obese children and adolescents with significant comorbidities be immediately enrolled in more advanced stages of treatment (such as a multidisciplinary care team) and this approach seems warranted in the WAGR population. Although growth chart percentiles for weight and height may be within normal range for patients, due to the associated short stature, BMI percentiles may be still abnormal. This has previously been described in a female patient who had normal weight for age (22%ile) but poor linear growth (height−3.1SD), which resulted in an abnormal weight-for-height ratio (94%ile) at 1 year of age and was considered at risk for development of true obesity (51). Multiple patients with WAGR have been described with rapid onset of childhood obesity in the literature, which suggests that more routine monitoring of anthropometric measurements may be warranted to identify patients who would benefit from referral to multidisciplinary care teams to help manage weight.

##### Potential Challenges to Weight Management Within WAGR Spectrum

Standard prevention and treatment for obesity and metabolic syndrome include a healthy diet/caloric intake and exercise, although several factors associated with WAGR syndrome can present challenges to these standard therapies, supporting the need for a multidisciplinary care team approach. Approximately a quarter of participants in the WAGR Discovery Cohort reported ataxia, which can lead to a variety of functional difficulties that complicate normal exercise, such as balance and walking issues and a recent systematic review found that evidence is lacking related to the effectiveness of physical therapy and exercise interventions for children with ataxia (52). Additionally, a substantial portion of participants reported issues with hypo- or hypertonia, toe-walking, spasticity, and scoliosis which highlights the need for neurological and orthopedic evaluations to determine whether any issues are present that could affect the ability to exercise. Orthopedics could help determine the best treatment interventions to provide support for safe activities and reduce further issues. Physical therapists and/or other exercise specialists can also help provide individualized intervention strategies.

The presence of intellectual disabilities and hyperphagia can complicate dietary management, which supports the role of a behavioral counselor in addition to a nutrition specialist within the care team. Parental supervision is also likely necessary in patients affected by hyperphagia. A case report described a patient with binge eating that was related to an increased opportunity for unsupervised eating (48). He was reported to steal food and appeared oblivious to satiety sensations (48). The use of food diaries in the WAGR population has also been cautioned for inaccuracy, as patients may seek food without parental knowledge due to hyperphagia (2).

##### Additional Considerations for Obesity

Despite these challenges, weight stabilization in patients with WAGR has been reported. In the first reported patient with WAGR and obesity, increased weight gain and severe obesity persisted despite hypocaloric diet intervention (10). A follow-up report of this same patient reported that weight stabilization was achieved through strict dietary and psychological management even with hyperphagia present, and her BMI had been reduced from the severe obesity range to the overweight range at 14 years of age (49).

Further evidence is needed to evaluate intervention strategies in patients with WAGR in order to determine whether an effective strategy for this patient population may exist. Theoretically, the effectiveness of treatment strategies for obesity in the WAGR population may be genotype-dependent. Increased circulating levels of *BDNF* with acute exercise have been well-established (53). As patients with WAGR affected by *BDNF* deletions have been found to have reduced serum *BDNF* concentrations compared to patients with WAGR and *BDNF* intact (2), exercise may provide a greater benefit to the latter subgroup of patients whereas those with *BDNF* deletions may benefit more from dietary interventions or other strategies as exercise may not be effective as a result of the compromised role that *BDNF* can provide.

##### Emerging Evidence for Altered Metabolism in WAGR Spectrum

Despite the established associations between obesity and CKD/ESRD in the WAGR population, it does not appear that the other adverse cardiometabolic characteristics such as hypertension and metabolic syndrome criteria have been evaluated (Supplementary Appendix). The characteristics reported by the WAGR Discovery Cohort participants suggest that at least 12% of the population have profiles consistent with metabolic syndrome/spectrum. While the role of *BDNF* deletion in obesity and other metabolic characteristics has been explored, it is also possible that the *PAX6* deletion may contribute to some of the metabolic abnormalities that can affect patients with WAGR Spectrum. Recently in families with congenital aniridia it has been suggested that *PAX6* mutations and glucose metabolism may be associated; with case-control suggesting that the production of proinsulin to insulin may include participation from the *PAX6* gene (54). Furthermore, this study identified signs of early subclinical beta-cell dysfunction and it was suggested that the participation of *PAX6* abnormalities may eventually lead to abnormal glucose metabolism in patients (54). Interestingly, it has been suggested in mice that PAX6 may directly or indirectly bind to promoter sequences for essential genes related to energy metabolism and immunological surveillance in the brain that alter during aging (55). While further evidence is needed to understand the role of the *PAX6* deletion in human models, these emerging observations suggest that patients with WAGR, aniridia, and possibly other chromosome 11p13 related disorders may experience abnormal metabolism as they age.

### Additional Health Care Considerations

Several other primary health issues are common in patients with WAGR spectrum, however specific recommendations for issues are less clear and these additional considerations are summarized in Table 8. An initial cardiac evaluation may be beneficial, as a variety of congenital cardiac anomalies can occur and a cardiology work-up prior to surgical procedures is recommended. A variety of feeding and other GI issues were reported by participants, which suggests that referral to gastroenterology may be beneficial. Musculoskeletal issues are common in patients and neurology and/or orthopedics can help manage specific issues.

#### Neurological Issues

Neurology evaluations are warranted in patients with WAGR spectrum, as a variety of issues can occur. The neurology team can help determine the appropriate testing and management for patients based on individual issues. The range of neurological issues is emerging in the population and a summary of common features reported in WAGR is available in the Supplementary Appendix.

##### Brain Abnormalities

In the present study, we observed six participants with reported agenesis of the corpus callosum as well as other brain abnormalities, which suggests a potential association. Support for this association is available through previous evidence. Fischbach et al. reported two patients affected by agenesis of the corpus callosum as well as a variety of structural brain abnormalities in their cohort (1). The only case report of prenatally-detected WAGR was affected by absent corpus callosum in addition to absent cavum pellucidum (56). In a case series of three patients with WAGR, two (monozygotic twins) were affected by partial or complete agenesis of the corpus callosum (57). In a previous cohort, hypoplastic corpus callosum has also been reported to occur in 30.8% of patients with WAGR (58). Abnormalities of the corpus callosum may contribute to a range of developmental issues such as language and emotion processing (58), and symptoms of autism have been associated (23).

##### Seizures

In the WAGR Discovery Cohort, 12 patients reported seizures for a frequency of 18.2% affected. Previously, Xu et al. reported three of 31 patients (9.7%) with WAGR affected by seizures; Dahan et al. reported one patient among seven (14.3%) with epilepsy; and Fischbach et al. reported epilepsy was present in four of 54 patients (7.4%) within their cohort (1, 5, 33). Taken together from these four studies, an estimated frequency of 12.7% of patients may experience seizures or epilepsy.

Patients with WAGR may have undiagnosed epilepsy or unrecognized seizures, as a patient affected by WAGR with obesity was described with signs of rolandic epilepsy of childhood demonstrated by electroencephalogram, despite the absence of seizures (59). Further research is needed to explore potential mechanisms leading to seizures in patients with WAGR syndrome, as a variety involving the common WAGR genes and *SLC1A2* and *ELP4* genes have been suggested (24, 60, 61).

#### Pulmonology Considerations

Patients with WAGR appear to be frequently affected by respiratory tract infections, pneumonia, and other lung/breathing issues; and close to a third of the cohort reported obstructive sleep apnea (OSA). Many of these issues were previously reported by Fischbach et al. (1), with a comparison summary provided in the Supplementary Appendix. In a previous case series, it was reported that all three patients affected by WAGR were found to have tracheomalacia after their parents noticed unusual stridor (62). In a more recent cohort of patients with WAGR and WT, there was one with reported “lung hypoplasia” (7). We found that seven participants reported abnormalities at birth that included underdevelopment of part of the respiratory system for an estimated overall proportion of 13% in the cohort. Airway malacia, which includes laryngomalacia, tracheomalacia, and bronchomalacia can increase the risk for issues such as OSA, recurrent respiratory infections, and pneumonia (63); issues that were all commonly reported in our cohort. It is possible that the presence of laryngomalacia is responsible for the high frequency of some of the GI issues reported by the cohort, as gastroesophageal reflux disease (GERD) represents the most common comorbidity with airway malacia and other non-respiratory issues associated include feeding difficulty and dysphagia (63).

As a result of these observations, it is suggested that patients with breathing concerns and/or frequent infections/pneumonia receive referral to a pulmonologist or other respiratory specialist to determine the presence of underlying causes. Referrals can also be considered for patients affected by feeding issues, dysphagia, and/or GERD without response to standard treatments to determine whether an underlying airway issue is the cause for the GI issues. A sleep study should also be considered for patients with abnormal sleeping habits or breathing concerns.

##### Potential Mechanisms Contributing to Pulmonary Issues

The *WT1* gene deletion may represent the mechanistic cause for the lung issues observed. Animal models have shown that *Wt1* is rapidly downregulated in mesothelial-derived cells, which contribute to pulmonary mesodermal tissues that include vascular and bronchial smooth muscle and tracheal cartilage (64). Additionally, *WT1* expression in the fetal lung has been demonstrated and it has been suggested that *WT1* may have a major role in human pleura and lung development (65).

#### Sleep Issues

Sleep issues are common in patients with WAGR spectrum, with abnormal sleep length the most common issue reported by participants. Mutations in *PAX6* have previously been associated with problems initiating and maintaining sleep (66). Smaller pineal size, lower melatonin secretion, and greater sleep disturbances has also been found in patients with WAGR and isolated aniridia with *PAX6* deletions compared to healthy control patients, suggesting that *PAX6* is involved in pineal development and circadian regulation (67).

##### Considerations for Sleep Management

In addition to the underlying *PAX6* deletion, a variety of other clinical issues associated with WAGR can affect sleep: beta-blockers for hypertension can suppress melatonin suppression; renal insufficiency can affect urinary melatonin clearance; and lack of light perception can affect circadian patterns (67). Melatonin replacement for improving sleep quality has been suggested in this population (67). Further evidence is needed to determine the best treatment strategies.

Obstructive sleep apnea (OSA) was reported by 30% of participants in the present study. Although it is likely that OSA alone is not responsible for sleep issues that can affect patients, the presence of OSA can contribute to abnormal sleep. As a result, in all patients with WAGR spectrum affected by sleep issues, a referral to pulmonology or a sleep medicine specialist is recommended to help determine the need for a sleep study to detect OSA and determine the best individual treatment approach.

#### Gastrologic Issues in WAGR Spectrum

The gastrointestinal (GI) system appears commonly affected in patients with WAGR with 76.3% of participants reporting at least one issue within the GI system category of the questionnaire. Fischbach et al. previously reported an association between GERD and WAGR (1), however many of the other GI problems we observed have not been described or well-characterized in previous cohorts (Supplementary Appendix). It appears that while the GI system is commonly affected there is a range in the types and severity of issues that may present. Abnormal bowel movement appears to be the most common manifestation with 52.6% reporting chronic constipation and 16.9% reporting chronic diarrhea. Some participants additionally reported irritable bowel syndrome (IBS), inflammatory bowel disease, and one of five participants reported gastroparesis (Supplementary Appendix).

Feeding issues were reported by 48.1% of participants and dysphagia was also reported by 16.2% of the Discovery Cohort suggesting that patients may experience problems related to food intake. Gastroenterologists and feeding specialists are required to identify the underlying reason for the GI problems that present in patients and determine the appropriate management strategy. It is possible that mismanagement of nutrition intake may contribute to development of obesity and the biometry of patients receiving Supplementary feeding should be carefully monitored to detect differences in growth chart trends indicative of increasing weight and/or BMI percentiles.

Proper nutrient intake is required to help patients maintain a healthy cardiometabolic profile and the combination of gastroenterologists, feeding specialists, and a weight management team can help assist in determining a patient's unique nutritional needs to support their health.

##### Potential Risk for Pancreatitis or Other Severe Gastrologic Issues

Pancreatitis was reported by 7.2% of the WAGR Discovery Cohort, with five participants affected (unknown whether acute or chronic). Previously, chronic pancreatitis was found in three of 54 patients (5.6%) with WAGR (1). The *PAX6* gene appears to play an important role in pancreas development (1) therefore, the *PAX6* deletion may represent an underlying risk for development of pancreatitis in patients with WAGR spectrum. Other gastrologic issues in WAGR have not been well-described in large cohorts, but evidence suggests that some patients may experience other severe gastrologic issues (Supplementary Appendix). In the WAGR Discovery Cohort, participants reported other serious issues such as diaphragmatic hernia (6.7%), intestinal malrotation (5.4%), and gallstones (5.3%); peptic ulcers were reported by one participant. Although we were unable to quantify the rate of all issues, we observed several GI problems reported in the free-text response by some participants which included: anal stenosis (*n* = 1), anorectal malformation (*n* = 1), and colon polyps (*n* = 1).

Further evidence is needed to establish risk—care teams should be aware of the possibility of these issues developing in patients with WAGR until further information is available. Performing an abdominal ultrasound instead of renal ultrasound could help provide surveillance and detection in patients who have clinical issues that may suggest an underlying GI abnormality is present.

#### Anesthesia Considerations

Patients with WAGR spectrum will likely undergo surgical procedures as a result of their disorder. Prior to anesthetic procedures, patients should receive a thorough evaluation, as a number of clinical issues can complicate management. In patients with these issues, procedures should be performed at a specialized center in order to properly manage patient care.

##### Potential Issues Complicating Management

In a previous case series, two of three patients with tracheomalacia experienced respiratory distress after general anesthesia and it was suggested that anesthesiologists caring for patients affected by WAGR should be alerted to this possibility (62). In the single WAGR Discovery Cohort participant who was not alive, the parent reported the cause of death was acute respiratory distress syndrome (ARDS) that occurred within 3 days after surgery for the third WT occurrence. Although she was affected by OSA, she had undergone “dozens” of surgical and anesthesia procedures in the past without any complications or major issues; additionally there was no history of airway malacia or other related abnormalities that may have contributed to the ARDS. Additional common issues in WAGR spectrum, such as cardiac defects, scoliosis, and hypotonia, may also complicate anesthesia management (68). Care teams should be aware of these potential issues when performing the pre-operative assessment, as well as when planning for operative and post-operative care management.

##### Potential Risk of Pancreatitis With Propofol

Acute pancreatitis after administration of propofol has been reported in two patients with WAGR and hypertriglyceridemia (69, 70). Although this does not establish a risk, until further evidence exists the possibility of this complication could be considered in patients with WAGR spectrum. The care management team can help determine whether use of propofol is appropriate with information collected during the pre-anesthesia evaluation. Further evidence is needed to establish whether risk is present in this population and to guide recommendations.

### Study Limitations

The results of this study can be considered preliminary evidence, as all information collected was self-reported and medical records were not evaluated to confirm the health issues reported, representing a limitation in our results. Another limitation is selection bias, as participants more affected by health issues may be more likely to complete the questionnaire than those with less health issues. A recent cancer registry-based study commented that involving parents of patients can provide a more complete picture of the “phenotypic spectrum of WAGR syndrome” (7), which provides strength in our results. Our results suggest a high number of common health conditions are present in this patient population and further evidence is needed to confirm these findings. The dataset provided did not include the participant ages at time of survey completion, and future studies should prioritize investigation into the age that common issues present in patients to help guide clinical care and follow-up.

### Future Considerations for WAGR Spectrum and Rare Disease Research

Demographic information regarding diversity groups and geographic regions represented in WAGR cohorts have been rarely reported in the literature (Supplementary Appendix). In the WAGR Discovery Cohort, although 93% of participants identified with “white” as their racial group ~21% of the cohort identified as Hispanic or Latin American. Previously, Han et al. reported 91% as “non-Hispanic white” among 33 patients in their cohort (2). Geographic regions of patients with WAGR have not previously been reported and we identified 16 unique countries/regions, with all continents besides Antarctica represented in the WAGR Discovery Cohort. These observations suggest that WAGR spectrum can occur in all populations across the world.

It is unclear whether there is a racial or ethnic predominance associated with WAGR Spectrum. In other rare disease syndrome/spectrum disorders, subtle differences in the phenotypic presentation and clinical features between patients of diverse populations have been described, including another cancer predisposition disorder caused by chromosome 11p15 abnormalities (71). The potential influence of social health determinants on the “genetic diagnostic odyssey” has also recently been considered (72). It is possible that differences between diverse populations may occur within the WAGR spectrum population, leading to underdiagnosis of other diversity groups; however, the rarity of the disorder and severe clinical complications may also lead to geographic differences in survival outcomes based on medical care and awareness of this complex rare disorder, creating a bias in the populations of patients and families available to learn from. Inclusion of demographic data is essential for follow-up studies, as further exploration could help clarify whether a difference between diversity groups exists within the WAGR population to improve the diagnostic approach and individualized care management.

## Conclusion

In the present study we compiled self-reported health information from 91 individuals affected by WAGR syndrome to create the WAGR Discovery Cohort, which represents the largest cohort evaluated to date. Our results build upon previous findings in the WAGR patient population and suggest that a spectrum of clinical features and health issues can affect patients, prompting reclassification of this genetic disorder from WAGR syndrome to WAGR spectrum. A multi-disciplinary care team model is needed to address the variety of common health issues in this population. Considerations common to all patients include the need for routine surveillance to manage cardiometabolic health and monitor for chronic kidney disease and Wilms tumor (WT) development.

## Data Availability Statement

The data that support the findings of this study are available from Coordination of Rare Diseases at Sanford (CoRDS). Restrictions apply to the availability of these data, which were used under agreement for this study. Requests to access the datasets should be directed to https://research.sanfordhealth.org/rare-disease-registry/researchers.

## Ethics Statement

Written informed consent was obtained from the individual(s), and minor(s)' legal guardian/next of kin, for the publication of any potentially identifiable images or data included in this article.

## Author Contributions

JG, JM, KT, and SK contributed to the initial conception and design of the WAGR Syndrome Registry. JK and KD contributed to the conception and design of the study. KD organized the study database, performed the data analysis, and wrote the first draft of the manuscript. JK and KT wrote sections of the manuscript. All authors contributed to interpretation of results, manuscript revision, read, and approved the submitted version.

## Funding

This study was funded by Alex's Lemonade Stand Foundation and Miranda's Mission. The Coordination of Rare Diseases at Sanford (CoRDS) is a rare disease registry wholly funded by Sanford Health, a hospital system headquartered in South Dakota.

## Conflict of Interest

The authors declare that the research was conducted in the absence of any commercial or financial relationships that could be construed as a potential conflict of interest.

## Publisher's Note

All claims expressed in this article are solely those of the authors and do not necessarily represent those of their affiliated organizations, or those of the publisher, the editors and the reviewers. Any product that may be evaluated in this article, or claim that may be made by its manufacturer, is not guaranteed or endorsed by the publisher.
