# Supplementary material for: Results From the WAGR Syndrome Patient Registry: Characterization of WAGR Spectrum and Recommendations for Care Management
Source: Front Pediatr. 2021 Dec 14;9:733018. doi: 10.3389/fped.2021.733018 (PMC8712693; doi:10.3389/fped.2021.733018)
Supplement: Supplementary file 1 [file Table_1.PDF]

frontiers

Supplemental Table S1. **Frequency of Genetic Abnormalities Reported by the WAGR Discovery Cohort (n=91).**

Type of Genetic Abnormality		Number of Participants
**INCLUDED**		**54**
**Single Gene Selected**		**9**
	WT1 deletion	3
	WT1 mutation	1
	PAX6 deletion	4
	BDNF deletion	1
**WT1 deletion + PAX6 deletion**		**19**
	Mosaic	1
	+ ELP4 deletion	1
	+ FSHB, DCDC1, ELP4, RCN1, LMO2, HIKP3[a]	1
**PAX6 deletion + BDNF deletion**		**5**
**WT1 deletion + PAX6 deletion + BDNF deletion**		**19**[b]
	Mosaic	2
	Translocation	1
	+ 3p22.3 deletion	1
**Other**		**2**
	Karyotype 46, XX, del (11) (p11.12 p 14.1)	1
	unknown	1
**UNDETERMINED**[c]		**4**
**EXCLUDED**		**33**
	NULL (no entry)	14
	Don't Know	19[d]

[a] Genes listed are deletions.
[b] Includes two participants grouped within category: one selected "PAX6 mutation" instead of deletion; one selected both "deletion" and "mutation" for WT1 and PAX6 genes.
[c] Inconsistent abnormalities were provided by participant between completed questionnaires.
[d] Includes two participants grouped within category who both selected "don't know" and additionally reported "PAX6 deletion"(n=1) and "PAX6 mutation" (n=1).
