# Supplementary material for: Results From the WAGR Syndrome Patient Registry: Characterization of WAGR Spectrum and Recommendations for Care Management
Source: Front Pediatr. 2021 Dec 14;9:733018. doi: 10.3389/fped.2021.733018 (PMC8712693; doi:10.3389/fped.2021.733018)
Supplement: Supplementary file 2 [file Table_2.PDF]

***Supplemental Table S2.* Additional Health Issues Reported by the WAGR Discovery Cohort.**
Table lists the number of participants reporting each issue within the health categories for the health status questions. Frequencies shown are the number of participants who were affected by the issue compared to those who were not affected by the issue.

HEALTH CATEGORY / ISSUE	PARTICIPANTS AFFECTED	FREQUENCY (%)
**CONGENITAL HEART DEFECTS**		
**Structural Defects**		
Atrial septal defect (ASD)	4/69	6%
Patent foramen ovale (PFO)	4/69	6%
Ventricular septal defect (VSD)	4/68	6%
**Other Cardiac Defects**		
Coarctation of the aorta	2/69	3%
Pulmonary valve stenosis	2/68	3%
Interrupted aortic arch	2/70	3%
Pulmonary atresia	1/68	2%
Pulmonary valve regurgitation	1/68	2%
Patent ductus arteriosus (PDA)	1/69	1%
**ENDOCRINE/METABOLIC ISSUES**		
Diabetes	6/74	8%
Precocious / Early puberty	5/70	7%
Glucose intolerance	4/71	6%
Low Testosterone	3/62	5%
Osteopenia	3/66	5%
Hyperglycemia	3/70	4%
Hypoglycemia	2/71	3%
Early menopause (females)	1/38	3%
Delayed puberty	1/65	2%
Tall stature	1/67	2%
Hyperthyroid	1/69	1%
Hypothyroid	1/69	1%
**GASTROINTESTINAL (GI) ISSUES**		
Diaphragmatic hernia	5/75	7%
Umbilical hernia	4/72	6%
Gallstones	4/75	5%
Intestinal malrotation	4/74	5%
Irritable bowel syndrome (IBS)	4/74	5%
Pyloric stenosis	2/74	3%
Inflammatory bowel disease	1/74	1%
Peptic ulcers	1/73	1%
Gastroparesis	1/5	-
**KIDNEY/RENAL ISSUES**		
Kidney stones	5/71	7%
Polycystic / Cystic kidney	4/72	6%
Malformed kidney	4/72	6%
Duplicate kidney	2/72	3%
Horseshoe kidney	2/71	3%
Renal agenesis	2/73	3%
Dilated renal pelvis	1/72	1%
**OTHER CONDITIONS**		
Pancreatitis	5/69	7%
Multiple hereditary exostoses (MHE)	4/69	6%
Anosmia	3/58	5%
Bronchiectasis	3/70	4%
Hemihyperplasia	3/67	4%
Intracranial hypertension / pseudotumor cerebri	2/67	3%
Chronic obstructive pulmonary disease (COPD)	2/71	3%
Cholecystectomy	2/67	3%
