# Supplementary material for: Results From the WAGR Syndrome Patient Registry: Characterization of WAGR Spectrum and Recommendations for Care Management
Source: Front Pediatr. 2021 Dec 14;9:733018. doi: 10.3389/fped.2021.733018 (PMC8712693; doi:10.3389/fped.2021.733018)
Supplement: Supplementary file 3 [file Table_3.PDF]

***Supplemental Table S3.* Additional Health Issues Reported by the WAGR Discovery Cohort: Allergy Problems, Dental Conditions, and Hand/Foot Conditions.**

Table lists the number of participants selecting each issue within the checkbox questions for health categories. Estimated frequencies are shown, and represent the number of participants who selected the checkbox (affected) compared to the number of participants who completed the question and did not select the checkbox (assumed not affected).

HEALTH CATEGORY / ISSUE	PARTICIPANTS AFFECTED	FREQUENCY (%)
**ALLERGY PROBLEMS**		
Animal dander / Dust allergy	6/84	7%
Candidiasis / Thrush	5/84	6%
Latex / Skin irritant allergy	3/84	4%
**DENTAL CONDITIONS**		
High number of cavities	6/68	9%
Missing teeth	5/68	7%
Extra teeth	4/68	6%
Widely spaced teeth	4/68	6%
Enamel hypoplasia	2/68	3%
Large teeth	2/68	3%
Tooth in palate/roof of mouth	1/68	1%
**HAND/FOOT CONDITIONS**		
Small hands/fingers	6/78	8%
Feet turned in	5/78	6%
Syndactyly	5/78	6%
Recurring ingrown toenails	4/78	5%
Fifth finger clinodactyly	4/78	5%
Small nails	4/78	5%
Polydactyly	4/78	5%
Dysplastic or unusual nails	3/78	4%
Contractures of fingers/toes	2/78	3%
**ADDITIONAL ISSUES REPORTED IN FREE-TEXT**		
Anal stenosis	1	-
Anorectal malformation	1	-
Situs inversus	1	-
Mesocardia	1	-
Colon polyps	1	-
Skin cancer	1	-
Sporadic diplegic cerebral palsy	1	-
