# Supplementary material for: Results From the WAGR Syndrome Patient Registry: Characterization of WAGR Spectrum and Recommendations for Care Management
Source: Front Pediatr. 2021 Dec 14;9:733018. doi: 10.3389/fped.2021.733018 (PMC8712693; doi:10.3389/fped.2021.733018)
Supplement: Supplementary file 4 [file Table_4.PDF]

frontiers

Supplemental Table S4. Summary of Established Risk Factors, Clinical Signs, and Diagnostic Work-Up for Chronic Kidney Disease (CKD) with Application to the WAGR Spectrum Population.

At-Risk Populations for CKD that Warrant Surveillance[1-3]		
Established Risk Factors for CKD and Application to WAGR Spectrum		
**Risk/Screening Category**	**Clinical Issue or Feature**	**WAGR Discovery Cohort Rate**
**At-Risk Populations: Meet Criteria for Annual CKD Screening**	Hypertension	35.4% (n=23/65)
	Diabetes	8.5% (n=6/71)
	Metabolic Syndrome/Spectrum	11.8% (n=8/68)
**Criteria that Warrant Consideration for Classification of At-Risk Populations**<br><br>(Annual screening should be considered)	Cardiovascular Disease (CVD)	62.9% (n=44/70)
	Obesity	52.7% (n=39/74)
	History of low birth weight	26.1% (n=18/69) < 2.6kg at birth
	Older age	**23% >18 years in cohort (n=21)**<br>66.7% develop obesity by 10 years [4]<br>CKD onset 2-13 years post-WT [5]<br>ESRD occurs >12 years of age [6]; Cumulative risk 52.8% for ESRD by 20 years of age [7]

**Other High-Risk Individuals for CKD Screening and Treatment Program Implementation**[2]	**Comorbidities**	High rate of clinical issues associated with CKD risk in WAGR population
	**Environmental exposures**	Treatment for WT and/or other medical consequences of issues
	**Genetic risk factors**	Chromosome 11p13 deletion<br>Abnormal *WT1* gene

Initial Diagnostic Evaluation in Patients with Suspected CKD [1]

'Clinical and Diagnostic Clues' Applied to WAGR Spectrum

**Findings Suggestive of CKD Risks and Etiology**	**Review of Systems (ROS)**	**WAGR Discovery Cohort Rate**
	**Recent Infections**	
Recent infection Glomerulonephritis	Frequent Illnesses	63.4% (n=45/71)
	Frequent colds	58.6% (n=41/70)
	Respiratory tract infections	61.6% (n=45/73)
	Pneumonia	50.0% (n=37/74)
	Frequent Pneumonia	30.1% (n=22/73)
	**Urination symptoms**	
UTIs, obstruction, or stone	Recurrent UTIs	20.5% (n=15/73)
	Difficulty emptying bladder	10.0% (n=7/70)
	Abnormal bladder size	4.4% (n=3/68)
	Kidney stones	7.0% (n=5/71)
	**Other Issues**	
Signs of autoimmune disease (skin rash, arthritis, etc)	Allergy problems	59.5% (n=50/84)
	Atopic dermatitis/eczema	22.6% (n=19/84)

Renal Ultrasonography Signs and Potential CKD Risk Etiologies [1]

'Clinical and Diagnostic Clues' Applied to WAGR Spectrum

Signs on Renal Ultrasonography (RUS)	Summary of Findings Suggestive of CKD Risks/ Etiology	WAGR Discovery Cohort Rate
General Findings	**MAY SHOW:** nephrocalcinosis, discrete stones, hydronephrosis, complex cysts or masses, etc	CAKUT: 38.5% (n=30/78)
Increased echogenicity	**MAY INDICATE:** cystic and/or renal disease	Polycystic/cystic kidneys: 5.6% (n=4/72)
Large kidneys	**GENERALLY INDICATES:** tumors, infiltrating diseases, and/or nephrotic syndrome	WT and/or NR: 54.5% (n=42/77)
Size disparities and scarring	**SUGGESTS:** vascular, urologic, or tubulointerstitial diseases due to stones or infection	FSGS: 19.4% (n=14/72)<br>Kidney stones: 7.0% (n=5/71)
Small hyperechoic kidneys	**GENERALLY INDICATES:** Long-standing CKD	Rate of Kidney Failure: 25.0% (n=17/68)<br>Short stature (47.8%, n=33/69) could be appreciated to contribute to the potential for smaller kidney sizes

Supporting References:

1. Gaitonde DY, Cook DL, Rivera IM. Chronic Kidney Disease: Detection and Evaluation. *Am Fam Physician* (2017) 96(12):776-83. Epub 2018/02/13. PubMed PMID: 29431364.
2. Shlipak MG, Tummalapalli SL, Boulware LE, Grams ME, Ix JH, Jha V, et al. The case for early identification and intervention of chronic kidney disease: conclusions from a Kidney Disease: Improving Global Outcomes (KDIGO) Controversies Conference. *Kidney Int* (2021) 99(1):34-47. Epub 2020/11/01. doi: 10.1016/j.kint.2020.10.012. PubMed PMID: 33127436.
3. Stevens PE, Levin A, Kidney Disease: Improving Global Outcomes Chronic Kidney Disease Guideline Development Work Group M. Evaluation and management of chronic kidney disease: synopsis of the kidney disease: improving global outcomes 2012 clinical practice guideline. *Ann Intern Med* (2013) 158(11):825-30. Epub 2013/06/05. doi: 10.7326/0003-4819-158-11-201306040-00007. PubMed PMID: 23732715.
4. Han JC, Liu QR, Jones M, Levinn RL, Menzie CM, Jefferson-George KS, et al. Brain-derived neurotrophic factor and obesity in the WAGR syndrome. *N Engl J Med* (2008) 359(9):918-27. Epub 2008/08/30. doi: 10.1056/NEJMoa0801119. PubMed PMID: 18753648; PubMed Central PMCID: PMCPMC2553704.
5. Hol JA, Jongmans MCJ, Sudour-Bonnange H, Ramirez-Villar GL, Chowdhury T, Rechnitzer C, et al. Clinical characteristics and outcomes of children with WAGR syndrome and Wilms tumor and/or nephroblastomatosis: The 30-year SIOP-RTSG experience. *Cancer* (2021) 127(4):628-38. Epub 2020/11/05. doi: 10.1002/cncr.33304. PubMed PMID: 33146894; PubMed Central PMCID: PMCPMC7894534.
6. Breslow NE, Collins AJ, Ritchey ML, Grigoriev YA, Peterson SM, Green DM. End stage renal disease in patients with Wilms tumor: results from the National Wilms Tumor Study Group and the United States Renal Data System. *J Urol* (2005) 174(5):1972-5. Epub 2005/10/12. doi: 10.1097/01.ju.0000176800.00994.3a. PubMed PMID: 16217371; PubMed Central PMCID: PMCPMC1483840.
7. Breslow NE, Norris R, Norkool PA, Kang T, Beckwith JB, Perlman EJ, et al. Characteristics and outcomes of children with the Wilms tumor-Aniridia syndrome: a report from the National Wilms Tumor Study Group. *J Clin Oncol* (2003) 21(24):4579-85. Epub 2003/12/16. doi: 10.1200/JCO.2003.06.096. PubMed PMID: 14673045.
