# Supplementary material for: Results From the WAGR Syndrome Patient Registry: Characterization of WAGR Spectrum and Recommendations for Care Management
Source: Front Pediatr. 2021 Dec 14;9:733018. doi: 10.3389/fped.2021.733018 (PMC8712693; doi:10.3389/fped.2021.733018)
Supplement: Supplementary file 5 [file Data_Sheet_1.PDF]

Appendix – Comparison of Studies and Historical Evolution of the WAGR Phenotype

Background:

A challenge in understanding the full phenotypic spectrum and clinical consequences within this rare disease population includes the primary focus of studies performed in patients with WAGR, including cohorts and specific data variables included in reports. It is likely that patients are represented in multiple cohorts, providing an 'overlap' bias that complicates the ability to understand generalizability to the full clinical spectrum of WAGR. The specific data variables and reporting style also vary between these studies, which presents an additional challenge to correlating findings or performing meta-analysis type studies. Despite these limitations, the population cohorts and observation periods of patients with WAGR previously reported can be combined to create a longitudinal progression of clinical issues that support the findings derived from the WAGR Syndrome Patient Registry observation period through May 2020.

The constellations of observations of population rates reported between 1964 and currently emerging between 2020-2021 suggest that the common clinical issues characterized for WAGR Spectrum have likely been present in patients from early descriptions, but these 'nonclassical clinical findings' (as termed by Fischbach et al.) were underappreciated in the past due to the research and medical community focusing on the 'classic' features of W-A-G-R that were initially described. This document contains details regarding the 80-year historical evolution of WAGR syndrome/spectrum.

Appendix Content Summary:

Section Description and Categories Evaluated	Page(s)
Studies Selected for Comparison	2
Demographic Population Characteristics Previously Reported	3
Rates of Common Features in Males and Females with WAGR	4
Kidney and Genitourinary (GU) Issues	5-10
Wilms Tumor and Precursor Development Characteristics	5
Wilms Tumor Screening and Outcome Characteristics	6-7
Kidney and Urinary Tract Anomalies and Risk for Chronic Kidney Disease	8-9
Common Genital Anomalies (Internal and External)	10
Table Summary of Common Issues Affecting the Kidneys, Genitals, and/or Urinary Tract in WAGR Spectrum Described Across Study Populations	11
Cardiac and Metabolic Health Characteristics	12-14
Cardiac Anomalies and Defects	12
Cardiometabolic Health Characteristics	13
Low Birth Weight and Small Stature	14
Craniofacial Characteristics	15
Eye Health - Common Issues other than Aniridia	16
Other Common Health Issues	17-20
Respiratory Issues and Health	17
Neurologic and Musculoskeletal	18
Gastrologic Findings	19-20
Supporting Reference List	20

Summary of Studies Selected for Historical Comparison:

- **1964 – First Description of WT and congenital anomalies (1):**
  - Study Purpose: Describe congenital anomalies associated with WT – first description of aniridia and GU association
  - Observation Period: January 1940 – July 1963
  - Cohort: anomalies described for 66 patients identified out of 440 reviewed
    - 6 patients had aniridia, representing 1.4% overall (n=6/440)
    - 15% of all patients with WT had at least one congenital anomaly (n=66/440)

- **1984 – First Observations of 'del 11p/aniridia complex' (2):**
  - Study Purpose: Provided description of three additional patients with 'dell 11p/aniridia complex' and summarized evidence collected from 37 patients with the disorder
  - Observation Period: available literature evidence from first reported patient through 1984
    - Three patients described were born in 1966, 1979, and 1982, respectively
  - Cohort: 37 patients with 'del 11p/aniridia complex' (24 male(XY); 64.9% males)

- **2003 – Characteristics and Outcomes in Patients with WAGR and WT (3):**
  - Study Purpose: Compare characteristics and evaluate long-term outcomes between WT survivors with WAGR (WAGR-WT) and those without WAGR ('no-WAGR') with data collected through the National Wilms Tumor Study Group (NWTSG)
  - Observation Period: October 1969 – June 2002
  - Cohort: 64 patients with WAGR-WT (27 female reported; 57.8% males)

- **2005 – End Stage Renal Disease (ESRD) in Patients with WAGR and WT (4):**
  - Study Purpose: Evaluate ESRD for patients with history of WT using NWTSG data correlated with USRDS (U.S. Renal Data System)
  - Observation Period: October 1969-September 1994 (follow-up through June 30, 2001)
  - Cohort Groups: evaluated patients with malformation syndromes (including WAGR) and/or GU anomalies (those without malformation syndrome diagnosed)

- **2005 – WAGR Syndrome Clinical Review (5):**
  - Study Purpose: Evaluate 'nonclassical' issues or complications that have not been reported 'in conjunction with [WAGR] syndrome' and provide care recommendations specific to WAGR population
  - Observation Period: Lifetime of patient – age range: 7 months to 42 years at time of review
    - Average age = 9.2 years
  - Cohort: 54 patients with WAGR (31 males; 57.4% males)

- **2008 – Obesity and WAGR Syndrome Association (6):**
  - Study Purpose: Evaluate the rate of obesity and its association with *BDNF* gene deletion
  - Observation Period: Data collected between June 2006 and September 2007
  - Cohort: 33 patients recruited through the International WAGR Syndrome Association (IWSA)

- **2021 – WAGR Outcomes and Characteristics (30-year SIOP-RTSG Experience) (7):**
  - Study Purpose: Evaluate outcomes and clinical characteristics of patients with WAGR and WT through the SIOP-RTSG registries (International Society of Pediatric Oncology Renal Tumor Study Group) and the SIOP-RTSG network
  - Observation Period: between 1989 and up to 2019
  - Cohort: 43 patients with WAGR and WT and/or nephroblastomatosis
    - 30 patients with additional clinical data collected through investigator questionnaire
      - Congenital abnormalities reported in 13 patients (43.3%) which included those other than aniridia

Demographic Population Characteristics Previously Reported:

Note: Studies in which the full population of WAGR patients; those describing only WAGR-WT survivors were excluded due to inability to generalize to population.

- **1964 – First Description of WT and congenital anomalies (1):**
  - Reported that all but 20 patients among the 440 children were 'Caucasian'
    - 4.5% of total cohort would be classified as 'other race/ethnicity'

Diversity in WAGR – Race or Ethnic Groups:

- **2005 – WAGR Syndrome Clinical Review (5):**
  - 'Demographic data regarding race and geographic region were not available for the majority of patients.'
- **2008 – Obesity and WAGR Syndrome Association (6):**
  - Collected self-reported information on 'race or ethnic group'
  - Rate of non-Hispanic white Iwith 95% CIs) by *BDNF* deletion status reported:
    - 95% (74%-100%) with *BDNF* deletion
    - 86% (57%-98%) without *BDNF* deletion
  - Summary Characteristics of Population Derived:
    - 90.9% non-Hispanic white (n=30/33)
    - 10% Other race or ethnic group (n=3/33)
- **WAGR Discovery Cohort (present study):**
  - Geographic Regions: 85 participants reported 16 different countries/regions
  - Diversity Characteristics:
    - Racial Groups: reported by 85 participants
      - 92.9% identified as 'white' (n=79/85)
      - 7.1% identified as 'other' (n=6/85)
    - Ethnicity: 20.8% identified as Hispanic or Latin American (n=10/48)

Ratios of Males and Females in WAGR Cohort Populations:

- **1984 – First Observations of 'del 11p/aniridia complex' (2):**
  - The sex ratio is *'much in favor of males'*

	**Population Size**	**Males**	**Females**	**Ratio Male:Female**
**1964** (Turleau et al.)	37	64.9% (n=24)	35.1% (n=13)	1.85 (M:F)
**2005** (Fischbach et al.)	54	57.4% (n=31)	42.6% (n=23)	1.35 (M:F)
**2008** (Han et al.)	33	48.5% (n=16)	51.5% (n=17)	0.94 (M:F)
**WAGR Discovery Cohort**	89	41.6% (n=37)	58.4% (n=52)	0.71 (M:F)

Summary of Findings:
Demographic data are infrequently reported to determine whether any incidence or prevalence differences exist between diversity groups and a WAGR diagnosis. Evidence from the WAGR Discovery Cohort suggests that WAGR can occur in all geographic regions, as we observed 16 different countries/regions represented including all continents besides Antarctica. Early characterization described a male predominance, however more recent cohorts suggest that there may be more female patients diagnosed with WAGR than male patients.

Application to WAGR Spectrum Population:
There is unclear evidence regarding whether there are any sex-related differences or geographic/diversity differences that may occur within the WAGR Spectrum population – future studies should include these data to provide additional information about these topics.

Rates of Common Features in Males and Females with WAGR:

Comparison of Classic W-A-G-R Phenotypic Features in Male and Female Populations:

	Male Patients with WAGR		Female Patients with WAGR	
	**Fischbach et al. (2005)**	**WAGR Discovery Cohort**	**Fischbach et al. (2005)**	**WAGR Discovery Cohort**
**WT Development (full WT)**	61.3% (n=19/31)	43.3% (n=13/30)	52.2% (n=12/23)	51.0% (n=24/47)
**Aniridia**	100% (n=31/31)	93.9% (n=31/33)	95.7% (n=22/23)	100% (n=50/50)
**GU anomalies**	90.3% (n=28/31)	84.8% (n=28/33)	34.8% (n=8/23)	39.5% (n=17/42)
**Cognitive Impairment**	77.4% (n=24/31)	69.0% (n=20/29)	65.2% (n=15/23)	51.0% (n=25/49)
**Chronic Kidney Disease Features**	29% (n=9/31)	32.3% (10/31)	21.7% (n=5/23)	42.9% (18/42)

Summary of Findings:

Although aniridia is the most constant feature, there has now been a female and two males without aniridia and a diagnosis of WAGR. Consistent rates of genital and urinary tract (GU) anomalies have been reported in cohorts for both males and females. Rates of full WT development appear constant for female patients, while a wider range has been observed for males with full WT development. It appears that the classic cognitive impairment severity of the 'R' phenotype may have been overrepresented in previous cohorts and patients may be affected by less severe cognitive delays; males consistently appear more affected by this feature than females.

Although CKD is not historically considered a 'classic feature' of WAGR, two studies have now evaluated the rates in males and females within the general WAGR population *(instead of focused on WAGR-WT survivor populations)*. Consistent rates in males have been reported between studies (approximately 30%); however, a range between 22-43% prevalence in females was observed.

Application to WAGR Spectrum Population:

Patients are commonly affected by the classic phenotypic features of the WAGR acronym, with consistent rates observed between the 2005 cohort and the WAGR Discovery Cohort. A less frequent, but important emerging phenotype of the WAGR population includes features of CKD, with both males and females commonly affected by at least one feature.

Wilms Tumor and Precursor Development Characteristics:

Supporting Evidence Collected from Selected Studies:

Wilms Tumor and WAGR Rates:

- **2005 – End Stage Renal Disease (ESRD) in Patients with WAGR and WT (4):**
  - Proportion of patients with WAGR within NWTSG (National Wilms Tumor Study Group) has remained constant at approx. 7-8 per 1,000
- **2005 – WAGR Syndrome Clinical Review (5):**
  - 61.3% Males with WAGR and WT development (n=19/31)
  - 52.2% Females with WAGR and WT development (n=12/23)
- **2021 – WAGR Outcomes and Characteristics (30-year SIOP-RTSG Experience) (7):**
  - Aniridia recorded in 47 of 7842 patients with WT (0.6% overall)
- **WAGR Discovery Cohort (present study):**
  - 43.3% Males with WAGR and full WT development (n=13/30)
  - 48.9% Females with WAGR and full WT development (n=23/47)

Nephrogenic Rests and/or Nephroblastomatosis:

- **2003 – Characteristics and Outcomes in Patients with WAGR and WT (3):**
  - *'The tumors of patients with and without WAGR syndrome differed remarkably in the nature and extent of their association with precursor lesions.'*
  - Most kidney specimens from patients with WAGR showed evidence of NR
    - 81% in WAGR compared to 42% in non-WAGR
- **2005 – WAGR Syndrome Clinical Review (5):**
  - Reported 2 patients with 'nephrogenic rests/nephroblastomatosis' in addition to 31 patients with WT development
- **2021 – WAGR Outcomes and Characteristics (30-year SIOP-RTSG Experience) (7):**
  - Histology available for 42 patients
    - 6 had nephroblastomatosis without further WT
    - 36 had WT and a variety of different histological types were reported
  - NR present in 78.9% of the 42 patients with histology
- **WAGR Discovery Cohort (present study):**
  - Nephrogenic rest without full WT reported by 7.8% of participants (n=6/77)
  - One additional patient with 'NR only' initially diagnosed was later diagnosed with WT

Adjusted Rates of Development of WT precursors and/or full WT development:

The number of patients with precursor and/or full WT development can be combined to evaluate the overall rate of development within the WAGR population cohorts:

- **2005 – WAGR Syndrome Clinical Review (5):**
  - 61.1% of all patients (n=33/54)
- **2021 – WAGR Outcomes and Characteristics (30-year SIOP-RTSG Experience) (7):**
  - 14.3% with nephroblastomatosis without further WT (n=6/42)
- **WAGR Discovery Cohort (present study):**
  - 54.5% Overall in WAGR with WT and/or NR (n=42/77)
  - 60.0% Males with WAGR and WT and/or development (n=18/30)
  - 51.0% Females with WAGR and WT and/or development (n=24/47)

Summary of Findings:

Patients with WAGR Spectrum experience risk for WT precursor development such as nephrogenic rests and/or nephroblastomatosis and supporting evidence for this is recently emerging. It is possible that the WT screening program may have contributed to early detection and intervention leading to these 'earlier WT disease forms' rather than full progression to WT at time of diagnosis.

Application to WAGR Spectrum Population:

WAGR Spectrum should be associated with risk for development of the spectrum of abnormalities leading to full WT. This risk includes precursor development such as nephrogenic rests (NR) to later manifestations such as nephroblastomatosis to full WT (unilateral or bilateral).

Wilms Tumor Screening and Outcome Characteristics

Supporting Evidence Collected from Selected Studies:

Role of WT Screening Program:

- **2003 – Characteristics and Outcomes in Patients with WAGR and WT (3):**
  - Patients with WAGR whose clinical record suggested that had not been screened had less favorable stage, age, and specimen weight compared to patients with WAGR diagnosed through screening *(specimen weight only with statistical difference)*
- **2021 – WAGR Outcomes and Characteristics (30-year SIOP-RTSG Experience) (7):**
  - 69.2% of patients diagnosed asymptomatic through screening (n=27/39)
  - 30.8% of patients presented with palpable abdominal mass or symptoms (n=12/39)
    - 3 patients with WAGR diagnosis prior to WT
    - 2 patients without WAGR diagnosed prior to WT

Outcomes in WT survivors with WAGR syndrome:

- **2003 – Characteristics and Outcomes in Patients with WAGR and WT (3):**
  - Initial clinical course for WAGR-WT was favorable
  - Longer-term outcomes less favorable
  - Starting at 9 years from diagnosis, the patients with WAGR *'began to die as a result of causes apparently unrelated to their cancer':*
    - There were 31 patients under long-term observation, and 15 experienced relapse and/or death:
      - 7 were affected by relapse, with two not surviving
        - One due to tumor; One due to congestive heart failure (CHF)
      - 10 in total did not survive (two with history of relapse) and eight without any relapse
        - ESRD (n=5); Infection (n=2); Respiratory arrest (n=1)

- **2021 – WAGR Outcomes and Characteristics (30-year SIOP-RTSG Experience) (7):**
  - 91.2% Overall Survival rate
  - 84.3% 5-year event-free survival rate
    - **Events (n=6) did not include relapse**
      - Contralateral tumor development (n=3) – occurring up to 7 years after initial diagnosis
      - Deaths (n=3) – one without exact cause but with obstructive ileus reported
        - Hepatotoxicity (n=2): both patients experienced hepatic failure prior to surgery because of sinusoidal obstruction syndrome during preoperative chemotherapy
  - Highlight value of surveillance for enabling NSS – there is a high rate of bilateral disease and risk of contralateral tumor development and comorbidity
  - Conclusion: *'Although they can be treated according to existing WT protocols, intensive monitoring of toxicity and surveillance of the remaining kidney(s) are advised.'*

Summary of Findings:

Patients with WAGR Spectrum appear to experience long-term risk for adverse health consequences such as tumor relapse and/or death due to other clinical factors present. The tumor screening program appears to provide a benefit for initial outcomes, however all patients with WAGR and WT appear to experience risk for CKD and ESRD, which may be related to the WT treatment and/or underlying 11p13 deletion.

Application to WAGR Spectrum Population:

Screening has been showed to provide the intended purpose within the WAGR population – with more favorable tumor characteristics identified in patients diagnosed through surveillance compared to symptomatic. Despite this benefit, a high rate of patients with WAGR and tumors who were not diagnosed through screening has recently been reported. This suggests that some patients may not be appreciated with WAGR phenotypes until after the classic 'WT phenotype' presents; additionally, some patients with WAGR may develop tumors at an age beyond the currently recommend screening interval. **Modifications to the WT screening program for the WAGR population are warranted**.

Kidney and Urinary Tract Anomalies and Risk for Chronic Kidney Disease (CKD)

Evidence Supporting Risk for CKD in WAGR Population:

- **1964 – First Description of WT and congenital anomalies (1):**
  - Variety of kidney/and or urinary tract anomalies other than genital observed (n=18)
    - Some of the anomalies of kidney and/or urinary tract observed not known to be associated with aniridia or hemihypertrophy (n=9 patients)

- **1984 – First Observations of 'del 11p/aniridia complex' (2):**
  - Renal malformations were reported in 4 patients among case series of 37 patients (estimated 23.5%)
    - Patient (case 2) with history of WT at 14 months and development of hypertension, cardiac failure and nephrotic syndrome at 11 years of age; Renal biopsy evidenced glomerular hyalinosis; she was alive at 17 years of age.

- **2003 – Characteristics and Outcomes in Patients with WAGR and WT (3):**
  - Adverse long-term outcomes in WAGR-WT survivors related to ESRD
  - Chronic renal failure detected in 14 patients among 64 patients
  - Cumulative risk of renal failure: 52.8% at 20 years (n=14/64)
    - 5 of 14 patients died as a result of ESRD
    - Revised from earlier report of 38% of 20 years (n=10/46)
  - Conclusions:
    - Little is (currently) known to explain the histopathology underlying WAGR-associated renal failure
      - Suggested that decreased *WT1* expression levels may be responsible for 'WAGR-associated renal disease'
    - Late mortality because of ESRD is significantly more frequent in WAGR-WT survivors.
      - Long-term surveillance of renal function in patients with WAGR should be performed to facilitate appropriate timing for intervention.

- **2005 – End Stage Renal Disease (ESRD) in Patients with WAGR and WT (4):**
  - Later development of ESRD is common in WAGR-WT survivors
    - Did not occur until at least 6 years from WT diagnosis (patient age >12 years)
  - In addition to WAGR, males with WT and hypospadias or cryptorchidism were also found to have a high risk for ESRD
    - The role of *WT1* gene was hypothesized as a potential influence
  - Only patients with WAGR and/or GU anomalies had substantial risk of ESRD beyond 10 years from WT diagnosis
    - ESRD tended to occur relatively late – during (or after) adolescence
  - **Recommendations**
    - Consider prospective study of renal structure/pathology by ultrasound in those who develop renal failure could help determine the extent of renal function decline
    - Long-term monitoring for kidney health is warranted through urine studies, hypertension management, and renal function level

- **2005 – WAGR Syndrome Clinical Review (5):**
  - 14 patients had 'some level of renal failure' for estimated 25.9% of cohort (n=14/54)
    - 29% in males (n=9)
    - 20% in females (n=5)
  - 4 males with transplant for ESRD (estimated rates: 12.9% of all males; 7.4% of cohort)
  - Features of CKD
    - 25.9% with Proteinuria (n=14/54)
    - 11.1% with FSGS (n=6/54)

- **2021 – WAGR Outcomes and Characteristics (30-year SIOP-RTSG Experience) (7):**
    - 25% had evidence of CKD (deceased eGFR and/or proteinuria)
        - Age of onset = 3-16 years old (post-WT diagnosis: 2-13 years)
    - Relationship between WT treatment history and CKD
        - Bilateral disease = 1 patient
        - Unilateral disease = 4 patients (n=1 with ESRD by age 16 years)

- **WAGR Discovery Cohort (present study):**
    - 51.3% of overall population affected by issue in the 'renal/kidney' category:
        - 38.4% with at least one feature of CKD (n=28/73)
        - 20.5% with recurrent urinary tract infections (UTIs)
    - 38.5% of overall population with issue consistent with CAKUT
    - Rates of CKD Features Reported:
        - 33.3% with proteinuria (n=24/72)
        - 25.0% with kidney failure (n=17/68)
        - 19.4% with FSGS (n=14/72)

<u>Summary of Findings:</u>
There is a clear association between WAGR and kidney disease and the risk for CKD development appears multifactorial. Although ESRD and renal failure has been mostly studied in the context of WAGR-WT survivors, the later age at development suggests that an accumulation of adverse health consequences caused by the 11p13 deletion is the only 'shared characteristic' that can currently be established as the most likely independent causative factor in the renal failure progression that affects patients within this population.

Early characterizations associated poor longer-term outcomes/death due to ERSD within the WAGR-WT population; it is possible the lack of information about WAGR-CKD that was not available until 2003-2005 (3-5) led to the unfavorable outcomes in these 'earlier generations' of patients with WAGR, as patients may not have received appropriate monitoring for this potential issue. A more encouraging finding is that more recent studies (7) and the observations from the WAGR Discovery Cohort suggest the currently living WAGR population has been diagnosed with less severe CKD states – providing an opportunity for mitigation strategies to try to prevent further renal failure and overall health status decline in the living generations of patients with WAGR, and improve overall care for future generations of patients with WAGR.

<u>Application to WAGR Spectrum Population:</u>
The 11p13 deletion in WAGR Spectrum creates a predisposition for abnormal kidney health. Patients with GU anomalies may have an additional risk compared to patients without GU anomalies; this feature should not be used to stratify potential 'risk groups' due to the high rate of internal GU anomalies within this population, and patients should be properly screened to detect potential internal GU anomalies that may contribute to kidney or urinary tract issues. There is long-term risk for CKD development/progression in patients with and without history of WT; all patients should be routinely monitored to evaluate their cardiometabolic and kidney structure profiles. Prompt detection of any abnormalities that can contribute to CKD should be thoroughly worked up and appropriately managed with the goal of preserving kidney function and maintaining a healthy cardiometabolic status.

Common Genital Anomalies (Internal and External)

<u>Common Descriptions in WAGR Populations:</u>

- **1964 – First Description of WT and congenital anomalies (1):**
  - Reported first association between aniridia, GU, and WT development
  - Variety of anomalies observed, with hypospadias and cryptorchidism common in males with WT development and congenital anomalies

- **1984 – First Observations of 'del 11p/aniridia complex' (2):**
  - Genital anomalies are *'practically constant in XY patients'*
  - Stressed importance of *'systematically searching for ambiguity of internal genitalia'*

- **2005 – WAGR Syndrome Clinical Review (5):**
  - Characterized a variety of male and female related genital anomalies
  - Reported 17% of females had an internal GU anomaly

- **2021 – WAGR Outcomes and Characteristics (30-year SIOP-RTSG Experience) (7):**
  - Male genital anomalies described
  - Ovarian cyst described in one patient

- **WAGR Discovery Cohort (present study):**
  - High rate of genital anomalies in both males and females
  - Internal female anomalies characterized in 34.1% overall *(double rate in Fischbach et al.)*

<u>Summary of Findings:</u>

There is a range of genital anomalies that can present in patients with WAGR, both external and/or internal. The rates of female internal GU anomalies have been increasing, which may reflect wider recognition of this classic phenotypic feature within the population. Genital anomalies affect both males and females.

<u>Application to WAGR Spectrum Population:</u>

Internal genital anomalies are common in males and females with WAGR Spectrum. Careful monitoring for detection of these anomalies is warranted, as some may not be easily recognized during infancy or early childhood.

Table Summary of Common Issues Affecting the Kidneys, Genitals, and/or Urinary Tract in WAGR Spectrum

	**Turleau et al. (1984)**	**Fischbach et al. (2005)**	**Hol et al. (2021)**	**WAGR Discovery Cohort**
**Population Size**	N=37	N=54 WAGR	N=30 WAGR-WT	N=91 WAGR
**Kidney and Urinary Tract Abnormalities**				38.5% (n=30/78) with CAKUT
Renal cysts	N=4 with renal malformations *(10.8% estimated frequency)*	1.9% (n=1)	3.3% (n=1)	5.6% (n=4/72)
Kidney stones		-	-	7.0% (n=5/71)
Renal agenesis or renal tissue disorganization		1.9% (n=1)	3.3% (n=1)	2.7% (n=2/73)
Ureteric reflux / VUR		-	3.3% (n=1)	9.9% (n=7/71)
Horseshoe kidney		-	3.3% (n=1)	2.8% (n=2/71)
Malformed or hypoplastic kidney		1.9% (n=1)	-	5.6% (n=4/72)
Ureteral duplication		1.9% (n=1)	-	6.1% (n=4/66)
**Ambiguous Genitalia**	25.0% (5/20)	9.3% (n=5)	-	10.8% (n=8/74)
Male Phenotype	-	6.5% (n=2)	-	22.6% (n=7/31)
Female Phenotype	-	13.0% (n=3)	-	2.3% (n=1/43)
**Male Genitalia**		N=31 Males	N=? Males	
Cryptorchidism	80.0% (20/25)	61.3% (n=19)	n=5	67.7% (n=21/31)
Hypospadius	6.0% (12/20)	12.9% (n=4)	n=2	35.5% (n=11/31)
**Female Genitalia**		N=23 Females	N=? Females	
Internal Anomalies		17% (n=4)	-	34.1% (n=14/41)
Streak ovaries	6.7% (1/15)	8.7% (n=2)	-	35.5% (n=11/31)
Bicornuate uterus	-	4.4% (n=1)	-	14.7% (n=5/34)
Small or hypoplastic uterus	-	4.4% (n=1)	-	6.3% (n=2/32)
Ovarian cysts or PCOS	-	-	n=1	7.7% (n=3/39)
**Gonadoblastoma**	N=2 (1 male, 1 female)	N=2 (1 male, 1 female)	None	Not thoroughly assessed (n=0/5)
**Chronic Kidney Disease**	Case 2	25.9% (n=14)	25.0% (n=5/20)	38.4% (n=28/73)
Proteinuria		25.9% (n=14)	≤25%	33.3% (n=24/72)
FSGS		11.1% (n=6)	-	19.4% (n=14/72)
**Emerging Issues**				
Recurrent UTIs	-	-	-	20.5% (n=15/73)
Difficulty emptying bladder	-	-	-	10.0% (n=7/70)
Abnormal bladder size	-	-	-	4.4% (n=3/68)

Cardiac Anomalies and Defects

Early Descriptions:

- **1964 – First Description of WT and congenital anomalies (1):**
  - Various cardiac anomalies described in patients presented
  - Additional anomalies reported from autopsy findings *(not diagnosed when alive)*
- **1984 – First Observations of 'del 11p/aniridia complex' (2):**
  - Reported 2 cardiac anomalies (cardiomyopathy; Fallot's tetralogy)
- **2005 – WAGR Syndrome Clinical Review (5):**
  - Reported one patient with Tetralogy of Fallot
- **2021 – WAGR Outcomes and Characteristics (30-year SIOP-RTSG Experience) (7):**
  - Reported mild pulmonary artery stenosis (n=1)

Cardiac Abnormality Type	Fischbach et al. (2005)	Hol et al. (2021)	WAGR Discovery Cohort
**Population Size**	N=54 WAGR	N=30 WAGR-WT	N=91 WAGR
**Structural Defects**			
ASD (atrial septal defect)	1.9% (n=1)	6.7% (n=2)	5.8% (n=4/69)
PFO (Patent foramen ovale)	3.7% (n=2)	-	5.8% (n=4/69)
VSD (ventricular septal defect)	3.7% (n=2)	-	5.9% (n=4/68)
Overall Rate in Category	<10% ($n \leq 5$)	-	15.7% (n=11/70)
**Other Defects**			
PDA (patent ductus arteriosus)	1.9% (n=1)	-	1.4% (n=1/69)
**Congenital Heart Defects**	-	-	22.5% (n=16/71)

Summary of Findings:

Individual patients or small numbers of patients with various cardiac anomalies have been reported in previous cohorts, however this feature has not been formally categorized. A variety of different types of anomalies can occur in patients. It appears that structural defects may represent the most common type of anomalies in patients.

Application to WAGR Spectrum Population:

Congenital heart defects may be more common in patients with WAGR Spectrum than previously appreciated, and structural defects appear most common. At time of diagnosis, patients should receive cardiac evaluation to monitor for potential abnormalities and determine appropriate care plan.

Cardiometabolic and Endocrine Health Characteristics

- **2008 – Obesity and WAGR Syndrome Association (6):**
  - 66.7% of children with WAGR developed obesity by 10 years of age (n=16/24)
    - 16 patients obese
    - 8 patients with normal weight
    - 9 patients <10 years
  - *BDNF* deletion found in 58% of all WAGR (n=19/33)
    - Patients with deletion had significantly higher BMI z-scores by 2 years of age compared to patients without *BDNF* deletion
    - Hyperphagia may be associated with *BDNF* deletion

**Cardiometabolic**	**Fischbach et al. (2005)**	**Hol et al. (2021)**	**WAGR Discovery Cohort (Present Study)**
**Population Size**	N=54 WAGR	N=30 WAGR-WT	N=91 WAGR
**Cardiometabolic**			
Hypertension	2 (3.7%)	-	35.4% (n=23/65)
Hyperlipidemia	3 (5.6%)	-	25.4% (n=17/67)
Obesity	10 (18.5%)	4 (13.3%)	52.7% (n=39/74)
**Endocrine**			
Diabetes or glucose intolerance	2 (3.7%)	1 (3.3%)	8.5% (n=6/71)
Hypothyroidism	-	3.3% (n=1)	1.4% (n=1/69)
Hyperthyroidism	-	-	1.4% (n=1/69)

<u>Summary of Findings:</u>
Obesity is common in patients with WAGR and tends to develop by 10 years of age, with an estimated 53-67% of all patients with WAGR and obesity. Adverse cardiovascular profiles also appear common. Although less common, some patients may be diagnosed with diabetes/glucose intolerance or issues with normal thyroid metabolism.

<u>Application to WAGR Spectrum Population:</u>
Patients experience risk for adverse cardiometabolic profiles and should receive appropriate monitoring and intervention to mitigate severity of issues. Other abnormal endocrine metabolism issues may develop in some patients.

Low Birth Weight and Small Stature:

Previous Descriptions in WAGR Populations:

- **1984 – First Observations of ‘del 11p/aniridia complex’ (2):**
  - Short stature or growth delay is ‘frequent’ with 9/14 patients <25%ile on growth charts (64.3% overall)
- **2003 – Characteristics and Outcomes in Patients with WAGR and WT (3):**
  - Patients with WAGR weighed <500 grams less at birth compared to non-WAGR
    - Weights at time of WT diagnosis were similar in WAGR to non-WAGR
  - WAGR was associated with shorter height at time of WT diagnosis
    - Height was 4.5 cm shorter on average for WAGR compared to non-WAGR
- **2021 – WAGR Outcomes and Characteristics (30-year SIOP-RTSG Experience) (7):**
  - Birth weight available for 15 patients:
    - 20% with birth weight < 10th percentile for gestational age (n=3/15)
    - ‘Normal range’ birthweight for other 12 patients
- **WAGR Discovery Cohort (present study):**
  - Short stature was reported by 47.8% of all participants (n=33/69)
  - Lower birthweight was reported by 26.1% of all participants (n=18/69)
    - 7.2% with birthweight between 1.8kg – 2.2kg (n=5/69)
    - 18.8% with birthweight between 2.3kg – 2.6kg (n=13/69)

	**Turleau et al. (1984)**	**Hol et al. (2021)**	**WAGR Discovery Cohort**
**Low Birth Weight**	-	20.0% (n=3/15)	26.1% (n=18/69)
**Short Stature**	64.3% (n=9/14)	-	47.8% (n=33/69)

Summary of Findings:
Short stature was associated with ‘del 11p/aniridia complex’ early on, however this characteristic has not been described across studies to the extent of other features. It appears that both low birth weight and/or short stature are common in patients with WAGR Spectrum.

Application to WAGR Spectrum:
Patients with WAGR may experience delayed growth and have a lower height (or shorter stature).

Craniofacial Characteristics:

Common Descriptions in WAGR Populations:

- **1964 – First Description of WT and congenital anomalies (1):**
  - Aniridia with: microcephaly (50%, n=3/6); with pinna or other ear deformities (33.3%, n=2/6)

- **1984 del 11p/aniridia complex) (2):**
  - Microcephaly reported in 2 patients
  - Described 'slight craniofacial dysmorphism' in Case 3, with a variety of features noted, including: *'a high arched palate'* and *'abnormal ears with a prominent antihelix and lobules on a plane perpendicular to the cranium (console-like lobules)*' in addition to other craniofacial abnormalities
  - *'There is no characteristic facial dysmorphism associated with the syndrome'* was stated in the discussion

- **2005 – WAGR Syndrome Clinical Review (5)**
  - Micrognathia (3.7%, n=2/54)
  - Palate abnormalities (5.6%, n=3/54)
    - Narrow palate (3.7%, n=2/54); Cleft palate (1.9%, n=1/54)
  - Microcephaly (1.9%, n=1/54)

- **2021 – WAGR Outcomes and Characteristics (30-year SIOP-RTSG Experience) (7):**
  - Macrocephaly reported in one patient (n=1)
  - Pierre-Robin sequence reported in one patient (n=1)

- **WAGR Discovery Cohort (present study):**
  - Craniofacial abnormalities at birth reported by an estimated 72.2% (n=39/54)

Comparison of Available Recent Craniofacial Characteristics:

	**Miller et al. (1964)**	**Turleau et al. (1984)**	**Fischbach et al. (2005)**	**WAGR Discovery Cohort**
**Population Size**	n=6	n=37	n=54	n=91
**Microcephaly**	50% (n=3)	5.4% (n=2)	1.9% (n=1)	18.5% (n=10/54)
**Ear abnormalities**	33.3% (n=2)	Case 3	-	48.1% (n=26/54)
**Mouth**				
Narrow or high-arched palate	-	Case 3	3.7% (n=2)	31.0% (n=18/58)
Malocclusion	-	-	16.7% (n=9)	10.3% (n=7/68)

Summary of Findings:
It is likely that some patients may share common craniofacial characteristics, and this may not have been recognized in the past. Evidence suggests that ear abnormalities and jaw/mouth abnormalities are likely part of the characteristic phenotype in WAGR syndrome/spectrum. There is likely a range of severity and specific features that led to these characteristics not appreciated in the past.

Application to WAGR Spectrum Population:
Further research is needed to characterize whether a facial phenotype can be associated with WAGR Spectrum to aid in clinical recognition and early diagnosis to begin necessary health screenings. Patients should be evaluated for craniofacial issues that may contribute to airway issues, adverse respiratory health status, or complications with feeding.

Eye Health - Common Issues other than Aniridia

Early Reports of Atypical Aniridia Characteristics and WAGR:

- **1964 – First Description of WT and congenital anomalies (1):**
    - 6 patients with congenital aniridia and WT development
    - 2/6 patients with *'incomplete aniridia'*
- **1984 – First Observations of 'del 11p/aniridia complex' (2):**
    - Aniridia reported as *'only constant clinical feature'*
    - Provided addendum reporting a patient with 11p deletion without aniridia

Early Descriptions of other Eye Issues:

- **1964 – First Description of WT and congenital anomalies (1):**
    - Cataracts reported in 4/6 patients with WT and aniridia (66.7%)
    - Glaucoma reported in 1/6 patients with WT and aniridia (16.7%)
- **1984 – First Observations of 'del 11p/aniridia complex' (2):**
    - Reported that 'practically all' patients with aniridia also have nystagmus, cataract, and/or glaucoma

Emerging Evidence for Eyes Issues in WAGR Spectrum Population:

Table. Comparison of Common Eye Issues reported in WAGR populations.

	**Fischbach et al. (2005)**	**Hol et al. (2021)**	**WAGR Discovery Cohort (Present Study)**
Population Size	n=54 WAGR	n=30 WAGR-WT	n=91 WAGR
No Aniridia	1.9% (n=1)	-	2.4% (n=2/83)
Nystagmus	40.7% (n=22)	16.7% (n=5)	93.9% (n=77/82)
Cataract(s)	66.7% (n=36)	33.3% (n=10)	86.1% (n=68/79)
Glaucoma	44.4% (n=24)	-	55.7% (n=41/75)
Optic nerve hypoplasia	14.8% (n=8)	3.3% (n=1)	40.7% (n=24/59)
Foveal/macular hypoplasia	13.0% (n=7)	-	28.4% (n=22/58)
Amblyopia	-	-	28.4% (n=21/74)
Strabismus	7.4% (n=4)	-	26.5% (n=18/68)
Corneal keratopathy/pannus	3.7% (n=2)	-	24.3% (n=17/70)
Peter's Anomaly	-	6.7% (n=2)	19.4% (n=13/67)
Retinal detachment	9.3% (n=5)	6.7% (n=2)	10.8% (n=8/74)

Summary of Findings:

Aniridia does not occur in every patient with WAGR Spectrum. Eye issues are common in addition to aniridia, and it appears that early focus was on issues such as cataract(s), glaucoma, and nystagmus; with more recent data supporting consistent or increasing rates within the WAGR population. We observed higher rates for most of the additional ocular issues described in cohorts, suggesting these may have been under-characterized previously. Retinal detachment has been reported consistently between 6-11% of patients.

Application to WAGR Spectrum Population:

Aniridia is not necessary for a diagnosis of WAGR Spectrum, and lack of aniridia does not exclude a diagnosis of WAGR Spectrum. Careful monitoring, prevention, and treatment of eye health should be prioritized to preserve vision and function.

Additional Common Health Issues

Other health characteristics are less described in WAGR populations, however the data emerging since 2005 suggests that some clinical issues and characteristics may be associated with WAGR. More evidence is needed to establish formal associations. In the interim, all patients with WAGR Spectrum should be considered and evaluated for the potential of these commonly observed issues. Identification of these issues will help provide information to develop the individualized care plan.

Pulmonary Issues, Frequent Illness, Hearing Loss, and ENT Procedures:

These features are emerging in recent cohorts and are less well characterized compared to some other features of WAGR, however common trends can be observed:

- **2021 – WAGR Outcomes and Characteristics (30-year SIOP-RTSG Experience) (7):**
    - Reported lung hypoplasia (n=1)

	**Fischbach et al. (2005)**	**WAGR Discovery Cohort**
**Population Size**	N=54	N=91
**Respiratory**		
Asthma	14.8% (n=8)	25.4% (n=18/71)
Obstructive Sleep Apnea (OSA)	20.4% (n=11)	30.1% (n=22/73)
**Respiratory Illnesses**		
Recurrent pneumonia	11.1% (n=6)	30.1% (n=22/73)
Recurrent sinusitis	27.8% (n=15)	-
Respiratory tract infections requiring antibiotics	-	61.6% (n=45/73)
**Hearing Loss**	3.7% (n=2)	14.7% (n=11/75)
**ENT Procedures**		
Tonsillectomy	40.7% (n=22)	44.3% (n=35/79)
Adenoidectomy		51.2% (n=41/80)
Tympanostomy tube placement	35.2% (n=19)	44.9% (n=35/78)

<u>Summary of Findings:</u>
Respiratory issues affecting breathing and recurrent infections/illness are common in patients with WAGR. Approximately 40-50% of the population appears to require an ENT procedure as part of their care management. The frequencies of tympanostomy tube placement procedure and hearing loss were higher in the present study than previously reported.

<u>Application to WAGR Spectrum:</u>
It appears that common respiratory issues occur in patients with WAGR and careful monitoring for development of these problems is necessary to facilitate proper health status. Although frequency of ear infections has not been evaluated, it is likely this may contribute to the high rate of tympanostomy tube placement reported by patients. These factors may contribute to the higher rate of hearing loss observed and routine hearing evaluations may be warranted.

Common Neurological and Musculoskeletal Issues:

Early Descriptions:

- **1984 del 11p/aniridia complex) (2):**
  - One patient with 'bilateral fibular polydactyly' reported among 37 (estimated 2.7%)
- **2005 – WAGR Syndrome Clinical Review (5)**
  - Reported variety of neurologic conditions in one or two patients

Recent Descriptions:

	**Fischbach et al. (2005)**	**Hol et al. (2021)**	**WAGR Discovery Cohort**
**Population Size**	N=54 WAGR	N=30 WAGR-WT	N=91 WAGR
**Neurological Issues**			
Hypotonia	13.0% (n=7)	3.3% (n=1)	55.9% (n=38/68)
Hypertonia		3.3% (n=1)	24.6% (n=17/69)
Epilepsy	7.4% (n=4)	3.3% (n=1)	Seizures reported by 18.1% (n=12/66)
Agenesis of the corpus callosum	3.7% (n=2)	-	11.1% (n=6/54)
Cerebral palsy	3.7% (n=2)	-	'sporadic diplegic cerebral palsy' reported in free text by n=1
**Musculoskeletal Issues**			
Polydactyly	-	6.7% (n=2)	5.2% (n=4/77)
Syndactyly	3.7% (n=2)	-	6.5% (n=5/77)
Clinodactyly		-	5.2% (n=4/77)
Scoliosis	14.8% (n=8)	-	19.7% (n=14/71)
Hemihypertrophy	5.6% (n=3)	-	4.5% (n=3/67)
Multiple hereditary exostoses (MHE)	3.7% (n=2)	-	5.8% (n=4/69)

Summary of Findings:
Neurological and/or musculoskeletal issues are common in the WAGR population, and it appears that rates of issues may have under-characterized in the past. Consistent rates of scoliosis have been reported (15-20% of patients).

Application to WAGR Spectrum Population:
Tone issues, including hypo- or hypertonia, appear common in patients. Some patients may experience seizures or be diagnosed with epilepsy. An emerging characteristic may include agenesis of the corpus callosum.

Musculoskeletal issues are common, and patients can present with a variety of abnormalities – patients should be monitored for scoliosis.

Common Gastrologic Findings:

- **2021 – WAGR Outcomes and Characteristics (30-year SIOP-RTSG Experience) (7):**
  - Three deaths in patients with WAGR-WT were related to GI abnormalities:
    - Hepatotoxicity (n=2)
    - Obstructive ileus (n=1)

	**Fischbach et al. (2005)**	**Hol et al. (2021)**	**WAGR Discovery Cohort**
Population Size	N=54 WAGR	N=30 WAGR-WT	N=91 WAGR
Pyloric stenosis	1.9% (n=1)	3.3% (n=1)	2.7% (n=2/74)
GERD	1.9% (n=1)	-	36.0% (n=27/75)
Pancreatitis	5.6% (n=3)	-	7.2% (n=5/69)

<u>Summary of Findings:</u>
Issues affecting the GI system are not well described in WAGR, however it appears that at least some patients may experience severe issues or complications.

<u>Application to WAGR Spectrum Population:</u>
Further characterization of the GI system within WAGR is needed – patients should be considered and worked up for potential GI-related issues depending on clinical status and previous history.

Emerging Characterization of Gastrologic Abnormalities in WAGR Spectrum:

An overall rate of 76.3% was observed in the WAGR Discovery Cohort for the presence of at least one gastrointestinal (GI) problem that was asked within the WAGR Syndrome Patient Registry questionnaire suggesting that GI abnormalities may represent a common phenotype. We summarized the rates of GI issues reported by the participants in **Table 5** and **Supplemental Tables S2-S3** to evaluate whether common categories of GI problems appear to exist within the WAGR Spectrum population.

	**WAGR Discovery Cohort**
**Affected by Gastrointestinal (GI) Problem**	76.3% (n=61/80)
**Food Intake Characteristics**	
Feeding problems	48.1% (n=38/79)
Gastroesophageal reflux disease (GERD)	36.0% (n=27/75)
Dysphagia	16.2% (n=12/74)
**Bowel Movement and Motility Issues**	
Chronic constipation	52.6% (n=41/78)
Chronic diarrhea	16.9% (n=13/77)
Irritable bowel syndrome (IBS)	5.4% (n=4/74)
Inflammatory bowel disease	1.4% (n=1/74)
**Other Complications**	
Pancreatitis	7.2% (n=5/69)
Intestinal malrotation	5.4% (n=4/74)
Gallstones	5.3% (n=4/75)
Gallbladder removal (cholecystectomy)	3.0% (n=2/67)
Pyloric stenosis	2.7% (n=2/74)
Peptic ulcers	1.4% (n=1/73)

**Abnormal Development of GI System**	
Diaphragmatic hernia	6.7% (n=5/75)
Umbilical hernia	5.6% (n=4/72)
**Emerging GI Characteristics**	
Gastroparesis	1 of 5 participants reported *(newer survey question)*
Anal stenosis	(n=1) reported in free text questionnaire section
Anorectal malformation	(n=1) reported in free text questionnaire section
Colon polyps	(n=1) reported in free text questionnaire section

Summary of Findings and Anecdotal Evidence from the International WAGR Syndrome Association (IWSA):
It does not appear that GI manifestations have been explored in previous WAGR cohort studies, or widely recognized in association with WAGR syndrome within the medical or research communities. The longitudinal observations and anecdotal information collected by the International WAGR Syndrome Association (IWSA) provide additional historical evidence that these issues have been a common concern among patients and families within the WAGR community for decades. This evidence suggests the results of the WAGR Discovery Cohort are likely not biased to the current population evaluated and represent an emerging characteristic of the full WAGR Spectrum.

Application to WAGR Spectrum Population:
It appears that abnormalities of the GI system represent a common phenotypic presentation within WAGR Spectrum. Common GI issues in the WAGR population include problems with feeding or food intake and issues with abnormal bowel movements and some patients may experience abnormal development of the GI system leading to hernias. More serious issues seem to affect a subset of the population.

Supporting Reference List:

1. Miller RW, Fraumeni JF, Jr., Manning MD. Association of Wilms's Tumor with Aniridia, Hemihypertrophy and Other Congenital Malformations. *N Engl J Med* (1964) 270:922-7. Epub 1964/04/30. doi: 10.1056/NEJM196404302701802. PubMed PMID: 14114111.
2. Turleau C, de Grouchy J, Tournade MF, Gagnadoux MF, Junien C. Del 11p/aniridia complex. Report of three patients and review of 37 observations from the literature. *Clin Genet* (1984) 26(4):356-62. Epub 1984/10/01. doi: 10.1111/j.1399-0004.1984.tb01071.x. PubMed PMID: 6094051.
3. Breslow NE, Norris R, Norkool PA, Kang T, Beckwith JB, Perlman EJ, et al. Characteristics and outcomes of children with the Wilms tumor-Aniridia syndrome: a report from the National Wilms Tumor Study Group. *J Clin Oncol* (2003) 21(24):4579-85. Epub 2003/12/16. doi: 10.1200/JCO.2003.06.096. PubMed PMID: 14673045.
4. Breslow NE, Collins AJ, Ritchey ML, Grigoriev YA, Peterson SM, Green DM. End stage renal disease in patients with Wilms tumor: results from the National Wilms Tumor Study Group and the United States Renal Data System. *J Urol* (2005) 174(5):1972-5. Epub 2005/10/12. doi: 10.1097/01.ju.0000176800.00994.3a. PubMed PMID: 16217371; PubMed Central PMCID: PMCPMC1483840.
5. Fischbach BV, Trout KL, Lewis J, Luis CA, Sika M. WAGR syndrome: a clinical review of 54 cases. *Pediatrics* (2005) 116(4):984-8. Epub 2005/10/04. doi: 10.1542/peds.2004-0467. PubMed PMID: 16199712.
6. Han JC, Liu QR, Jones M, Levinn RL, Menzie CM, Jefferson-George KS, et al. Brain-derived neurotrophic factor and obesity in the WAGR syndrome. *N Engl J Med* (2008) 359(9):918-27. Epub 2008/08/30. doi: 10.1056/NEJMoa0801119. PubMed PMID: 18753648; PubMed Central PMCID: PMCPMC2553704.
7. Hol JA, Jongmans MCJ, Sudour-Bonnange H, Ramirez-Villar GL, Chowdhury T, Rechnitzer C, et al. Clinical characteristics and outcomes of children with WAGR syndrome and Wilms tumor and/or nephroblastomatosis: The 30-year SIOP-RTSG experience. *Cancer* (2021) 127(4):628-38. Epub 2020/11/05. doi: 10.1002/cncr.33304. PubMed PMID: 33146894; PubMed Central PMCID: PMCPMC7894534.
